# Tacrolimus (FK506) Prevents Early Stages of Ethanol Induced Hepatic Fibrosis by Targeting LARP6 Dependent Mechanism of Collagen Synthesis

**DOI:** 10.1371/journal.pone.0065897

**Published:** 2013-06-03

**Authors:** Zarko Manojlovic, John Blackmon, Branko Stefanovic

**Affiliations:** Department of Biomedical Sciences, College of Medicine, Florida State University, Tallahassee, Florida, United States of America; Northwestern University, United States of America

## Abstract

Tacrolimus (FK506) is a widely used immunosuppressive drug. Its effects on hepatic fibrosis have been controversial and attributed to immunosuppression. We show that in vitro FK506, inhibited synthesis of type I collagen polypeptides, without affecting expression of collagen mRNAs. In vivo, administration of FK506 at a dose of 4 mg/kg completely prevented development of alcohol/carbon tetrachloride induced liver fibrosis in rats. Activation of hepatic stellate cells (HSCs) was absent in the FK506 treated livers and expression of collagen α2(I) mRNA was at normal levels. Collagen α1(I) mRNA was increased in the FK506 treated livers, but this mRNA was not translated into α1(I) polypeptide. No significant inflammation was associated with the fibrosis model used. FK506 binding protein 3 (FKBP3) is one of cellular proteins which binds FK506 with high affinity. We discovered that FKBP3 interacts with LARP6 and LARP6 is the major regulator of translation and stability of collagen mRNAs. In the presence of FK506 the interaction between FKBP3 and LARP6 is weakened and so is the pull down of collagen mRNAs with FKBP3. We postulate that FK506 inactivates FKBP3 and that lack of interaction of LARP6 and FKBP3 results in aberrant translation of collagen mRNAs and prevention of fibrosis. This is the first report of such activity of FK506 and may renew the interest in using this drug to alleviate hepatic fibrosis.

## Introduction

Fibroprolifirative disorders can target any organ system, ultimately leading to organ failure, and are a major contributor to significant morbidity and mortality worldwide [1], [2]. Despite all the medical advances in the last several years, there are only supportive therapies, with no approved, effective and specific antifibrotic treatments available [3]. Normal wound healing is initiated by tissue injury and inflammation with release of fibrogenic cytokines, resulting in proliferative activation of fibroblasts and deposition of extracellular matrix (ECM) proteins [4]. Liver fibrosis is an out of control wound healing response that is usually irreversible [5]. During liver injury, quiescent hepatic stellate cells (HSC), which normally store vitamin A, activate and differentiate into myofibroblast-like cells [6]. Activated HSC undergo proliferation and are the major cell type responsible for hepatic fibrogenesis [7].

Pathogenesis of alcoholic liver disease is mediated by free radicals and suppression of innate immunity [8], [9]. Alcohol metabolism in hepatocytes results in production of reactive oxygen species and acetaldehyde that can directly activate HSCs. In addition, alcohol results in increased uptake of lipopolysaccharides (LPS) from the gut flora. Increased levels of LPS trigger Kupffer cells to release pro-inflammatory cytokines, such as tumor necrosis factor-alpha (TNF-α), interleukins (IL-6 and IL-1), as well as pro-fibrotic cytokines, such as TGF-β [10], [11]. Although several collagen types are secreted by activated HSC, type I collagen is the most abundant and responsible for clinical manifestations of liver fibrosis, as well as manifestations of other fibroprolifirative disorders [4], [12].

The excessive collagen deposition in hepatic fibrosis is primarily due to the dramatic up-regulation of type I collagen synthesis at the post-transcriptional level [13]. This includes stabilization and more efficient translation of collagen mRNAs [13]–[16]. Type I collagen is a heterotrimer composed of two α1(I) and one α2(I) polypeptides and is the most abundant protein in the human body [17]. mRNAs encoding for type I collagen have an unique 5′ stem loop structure (5′SL) in their 5′ untranslated regions that contains the start codon [16]. Our lab has cloned and characterized La ribonucleoprotein domain family member 6 (LARP6) as the protein which binds 5′SL and regulates translation of type I collagen mRNAs [18]. LARP6 binds the 5′SL of collagen mRNAs with high affinity and specificity and is the central component of a ribonucleoprotein complex that assembles on the 5′SL [18]. LARP6 associates collagen mRNAs with two types of cytoskeletal filaments: with intermediate filaments composed of vimentin that prolonged the half-life of collagen mRNAs and with nonmuscle myosin filaments required for synthesis of natural heterotrimer of type I collagen [19], [20]. Disulfide bonding and post-translational modifications of collagen polypeptides take place during the translational elongation phase and before the heterotrimer is released into the lumen of the endoplasmic reticulum (ER) [21]. We postulated that nonmuscle myosin filaments facilitate translation of collagen α1(I) and α2(I) mRNAs within the sub-compartments of the ER to allow coordinated synthesis and folding of the heterotrimeric type I collagen [19].

In the past years great efforts have been made to understand the molecular mechanism of collagen synthesis [18]–[20], [22]. However, there has been no report on involvement of the FK506 binding proteins (FKBPs) in this process. FKBPs represent a superfamily of proteins that are implicated in T-Cell activation, ribosome biogenesis, tumor suppression, and transcription regulation [23]–[26]. All FKBPs have cis-trans prolyl isomerase (PPIase) activity and bind multiple immunosuppressant drugs, like rapamycin, FK506 (tacrolimus) and cyclosporin A [24], [27].

Tacrolimus (FK506), a macrolide antibiotic with potent immunosuppressive effects was isolated from *Streptomyces tsukubaensis* and has been previously used to prevent allograft and for treatment of autoimmune disorders in humans [28]–[30]. Animal studies have indicated that FK506 can inhibit neutrophil infiltration, reduce free radicals, decrease generation of reactive oxygen species and suppress pro-inflammatory cascade [31]–[35], thus, potentially affecting the factors which mediate alcoholic liver injury. A study on pulmonary fibrosis in mice suggested that FK506 can be a potent antifibrotic agent [36]. However, studies on liver fibrosis induced by bile duct ligation or by carbon tetrachloride (CCL_4_) administration gave conflicting results [37], [38].

Here we show that FK506 can prevent the development of alcohol induced liver fibrosis in rats by directly targeting collagen synthesis. We provide evidence that FK506 affects the LARP6 dependent mechanism of collagen synthesis, resulting in absence of fibrosis and minimal activation of HSCs. These results suggest a novel mechanism of action of FK506 in alcoholic liver fibrosis and may renew the interest of using FK506 as a potential antifibrotic drug.

## Materials and Methods

### Chemicals

FK506 (LC Laboratories) was dissolved in DMSO at 5 mM and stored in −20°C for in-vitro studies. For animal injections FK506 was dissolved in 10% Chremaphor (Sigma-Aldrich) and sterilized by filtration. Pure ethanol used in drinking water was purchased by Pharmaco-AAPER. Carbon tetrachloride (HPLC grade) was from Sigma-Aldrich.

### Cells and Transfections

Human HSCs were described before [39]. Rat HSCs were isolated by perfusion of rat livers with pronase and collagenase, followed by centrifugation on Nykodenz gradient, as described [40]. HEK293 cells and HLFs were also described before [22], [41]. The cells were grown under the standard conditions [18]. HEK293 cells were transfected with 1 µg of LARP6 constructs per 35 mm culture dish using 293TransIT reagent (Mirus). The cells were harvested 48 to 72 hours after the transfection. LARP6 constructs were cloned into pCDNA3 vector (Stratagene) having the N-terminal HA tag and were described previously [18], [22]. For FK506 treatment, cells were incubated with the indicated concentrations of FK506 for 24 h. The cells were then washed 3 times with PBS and incubated for 3 h in serum free medium to accumulate secreted collagen. The medium and cells were collected for western blot or RT-PCR analysis.

### Precision cut liver slices

Precision cut liver slices were cut from normal rat livers using the well-established method [42], [43] and Krumdieck microtome. The slices were cultured in DMEM supplemented with 10% FBS, 0.4 µg/ml dexamethasone, 50 µg/ml ascorbic acid, 0.5 µg/ml insulin and the medium was changed daily.

### Reverse Transcription-Polymerase Chain Reaction (RT-PCR) analysis

Total RNA was extracted using RNA isolation kit (Sigma-Aldrich). The extraction of rat liver RNA was done using TRI-Reagent (Sigma), as per manufacturer protocol. The RNA was treated with DNaseI to remove contaminating DNA. For semi-quantitative RT-PCR, 100 ng of total RNA was reverse transcribed using rTth polymerase (Boca Scientific) and the gene specific primer (Table 1). The PCR amplification was done in presence of [α^32^P]-dATP and radiolabeled PCR products were resolved on a sequencing gel and visualized by autoradiography, as previously described [18], [20]. The identity of the PCR products was confirmed by expected size or by sequencing.

**Table 1:** Primers used for RT-PCR analysis.

h-collagen α1 (I)	F: AGAGGCGAAGGCAACAGTCG R: GCAGGGCCAATGTCTAGTCC
h-collagen α2 (I)	F: CTTCGTGCCTAGCAACATGC R: TCAACACCATCTCTGCCTCG
r-collagen α1 (I)	F: TGAGCCAGCAGATTGAGAAC R: TGATGGCATCCAGGTTGCAG
r-collagen α2 (I)	F: CTCACTCCTGAAGGCTCTAG R: CTCCTAACCAGACATGCTTG
h-actin	F: GTGCGTGACATTAAGGAGAAG R: GAAGGTAGTTTCGTGGATGCC
r-actin	F: CGTGCGTGACATTAAAGAGAAGC R: TGCATGCCACAGGATTCCATACC
r-αSMA	F: ACAGAGAGAAGATGACGCAG R: GGAAGATGATGCAGCAGTAG
r-CD64	F: GGATCATACTGGTGCGAGGT R: TTGCTTTCTTCCCCTTCTCA
r-CD68	F: CAAAAAGGCTGCCACTCTTC R: GTGGGAGAAACTGTGGCATT
r-TNF-α	F: AGATGTGGAACTGGCAGAGG R: CCCATTTGGGAACTTCTCCT
r-interleukin 1β	F: CTGTGACTCGTGGGATGATG R: GGGATTTTGTCGTTGCTTG
r-interleukin 6	F: CCGGAGAGGAGACTTCACAG R: ACAGTGCATCATCGCTGTTC
r-lipopolysaccharide binding protein	F: AAGGCGCAAGTGAGACTGAT R: AGTCGAGGTCGTGGAGCTTA

F: forward primer, R: reverse primer.

For quantitative real time RT-PCR (qRT-PCR), equal amount of RNA (100 ng) was reverse transcribed using SuperScript II RT (Invitrogen). Five percent of the cDNA was used in qRT-PCR (BioRad-IQ5 Thermocycler) with the primers indicated in Table 1. The qRT-PCR was performed in duplicates and the threshold cycle (C_T_) and statistical analysis were computed using IQ-5 software (BioRad) and GraphPad Prism 3.02, as previously described [22].

### Western blotting

Cells and liver tissue were lysed in RIPA buffer (50 mM Tris pH 7.4, 150 mM NaCl, 0.5% sodium deoxycholate, 1% NP-40, 0.1% SDS and 1 mM EDTA), supplemented with protease inhibitor cocktail (Sigma). 50 µg of cellular extract or of whole liver extract was typically analyzed. For analysis of cellular medium, cells were placed in serum free medium and collagen accumulation was allowed to proceed for 3 h. After that, 45 µL of the medium was directly loaded onto the SDS-PAGE gel. Antibodies used were: anti-collagen α1(I) antibody from Rockland), anti-collagen α2 (I) from Santa Cruz Biotechnology, anti-fibronectin antibody from BD Transduction Laboratories, anti-tubulin antibody from Cell Signaling, anti-LARP6 antibody from Abnova, anti-HA antibody from Sigma-Aldrich, anti-FKBP3 antibody from Abcam, anti-actin antibody and anti-αSMA antibody from Abnova.

### Immunoprecipitations (IP)

Cells were lysed in isotonic buffer for 1 h at 4°C and 1 mg of total protein was incubated with 1 µg of the specific antibody, followed by incubation for 4 h with 20 µL of equilibrated protein A/G beads (Santa Cruz Biotechnology). The beads were washed three times with PBS supplemented with 0.5% NP-40 and analyzed by western blotting. Co-precipitation of collagen mRNAs was analyzed by extracting total RNA from the immunoprecipitation reactions and performing semi-quantitative RT-PCR and qRT-PCR reactions. All immunoprecipitations were repeated in two independent experiments.

### Animal Study

Male inbred alki Wistar rats weighing 150–200 grams and approximately 50 days of age were obtained from Charles River. The protocol for this study was approved by the Florida State University Animal Care and Use Committee; protocol number 1119. All animal procedures and experiments were performed under the NIH guidelines for animal care and use. This study has been specifically approved by the Florida State University Animal Care and Use Committee. The animals were acclimated for 5 days upon arrival by housing with 12 h dark-light cycle and receiving standard chow diet with ab libitum access to water and food. At the start of the study, drinking water was replaced by 5% ethanol as the only source of liquid and the rats were allowed to drink at will. Alcohol containing water was changed every 3 days to maintain the 5% ethanol level. The liquid intake and body weight was recorded daily. Carbon tetrachloride (CCl_4_) was administered intraperitoneally (i.p.) at 0.5 µl/g of CCl_4_ in mineral oil twice a week for 4 weeks [44], [45]. FK506 was dissolved in 200 µL of Cremaphor (Sigma) and injected at daily dose of 4 mg/kg for 4 weeks (28 days). Control animals received by i.p. injections the Cremaphor vehicle. Eight animals were used per condition (4 conditions total) to average for the biological variability and for statistical analysis. The groups were as follows: group 1∶ 5% ethanol liquid+CCl_4_ injections; group 2∶ 5% ethanol liquid+CCl_4_ injections+FK506 treatment; group 3: FK506 treatment only; group 4: vehicle only. Four weeks after the start of treatment and 24 h after the last injection the animals will be deeply anesthetized to avoid suffering with 80–140 mg/kg Ketamine+10 mg/kg Xylazine. The depth of anesthesia was measured by checking for the absence of the pedal/tail withdrawal reflexes. Blood was collected by puncture of the abdominal aorta. Animals were euthanized by exsanguination after blood collection, followed by liver removal. A part of the largest liver lobe was fixed in 10% formalin for histological analysis. Total RNA and protein were extracted from parts of this and other liver lobes and analyzed by RT-PCR and western blotting.

### Histology Analysis

Fixed liver tissue was embedded in paraffin and 10 µM thick slices were placed on microscope slides. Slices were prepared for H&E and Masson's trichrome staining. The degree of hepatic fibrosis was and other histological changes were evaluated by one of the authors (J.B.). Quantification of the degree of fibrosis was done using Image J software with Treshold-color plug-in logarithm. Percent fibrosis was calculated by dividing the area of fibrosis (blue regions) by the total area.

### ALT and AST Determination

ALT and AST were measured by standard kinetic protocol #2920 and #2930 using the Stanbio Laboratory reagents. Positive and negative controls were included in the analysis and all samples were analyzed in duplicates.

### Statistics

The equation for the determination of Standard Error was computed using biostatistics program GraphPad Prism 3.0 or Microsoft Excel. The statistical significance between groups for the in-vivo study was determined by analysis of variance (ANOVA). Type I error (α) and type II error (β) were set at 0.05 and 0.01 respectively. Student's t-test was used to assess the statistical significance between two groups. The statistical significance was determined to be at p<0.05 and the error bars shown in figures represent±1 SD.

### Abbreviations

The abbreviations used in this manuscript are listed in Table 2.

**Table 2:** Abbreviations.

**α1(I)** – alpha 1 chain of type I collagen
**α2(I)** – alpha 2 chain of type I collagen
**αSMA -** alpha smooth muscle actin
**CCL_4_** **-** carbon tetrachloride
**ECM** - extracellular matrix
**ER** - endoplasmic reticulum
**EtOH** – ethanol
**FKBP3** – FK506 binding protein 3
**HSC** - hepatic stellate cells
**HLF** - human lung fibroblasts
**i.p.** – intraperitoneal
**IP** – immunoprecipitation;
**LARP6** - La ribonucleoprotein domain family member 6
**LPS** – lipopolysaccharides
**LPS-BP** **-** lipopolysaccharide binding protein
**TNF-α** - tumor necrosis factor-alpha
**TGFβ** – transforming growth factor beta
**PPIase** – peptidyl-prolyl isomerase
**5**′**SL** - 5′ stem loop
**ALT** – alanine aminotransferase
**AST** – aspartate aminotransferase

## Results

### FK506 reduces synthesis of type I collagen protein without affecting expression of collagen mRNAs

**Figure 1:**
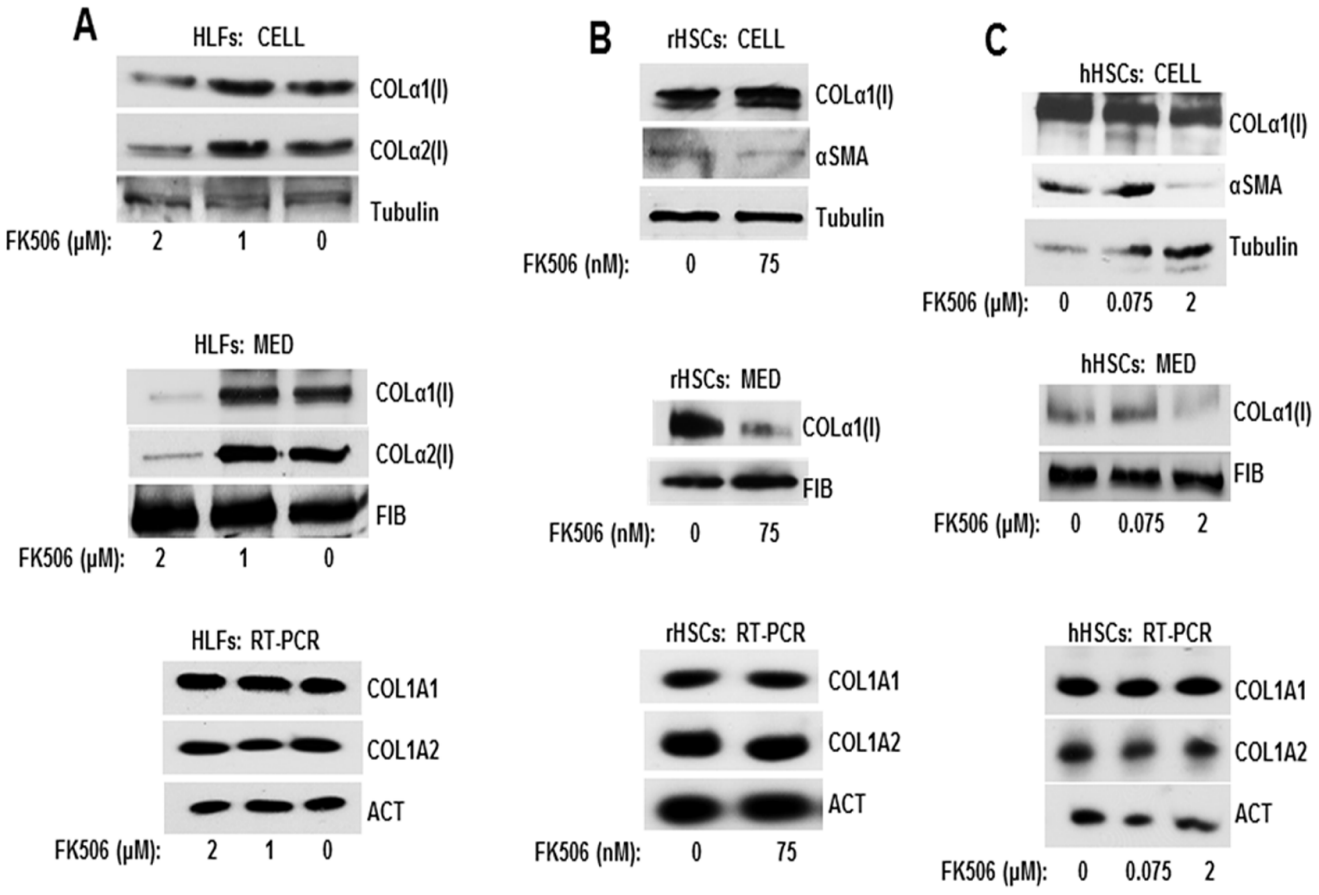
FK506 inhibits secretion of collagen polypeptides into cellular medium. A. Western blot analysis of collagen α1(I) and α2(I) polypetides in cellular extracts and in the medium of human lung fibroblasts (HLF) treated with the indicated concentrations of FK506. Top panel: western blot of cellular collagen. Loading control: tubulin. Middle panel: Western blot of collagen secreted into the cellular medium. Loading control, fibronectin (FIB). Lower panel: RT-PCR analysis of the collagen mRNAs level in HLFs. Actin mRNA (ACT) is shown as loading control. B. Western blot analysis of collagen α1(I) polypeptide in cellular extracts and in the medium of primary activated rat HSCs (rHSCs) treated with the indicated concentrations of FK506. Top panel: Collagen α1(I) polypeptide in cellular extracts. α-smooth musle actin (αSMA) was analyzed as a marker of HSCs activation and tubulin as loading control. Middle panel: secretion of collagen α1(I) polypeptide into the medium of rHSCs. Lower panel: RT-PCR analysis of the collagen mRNAs level in rHSCs. C. Same analysis as in B, except activated human hepatic stellate cell line (hHSC) was used.

The initial discovery that FK506 can reduce collagen expression in vitro was observed in human lung fibroblasts (HLFs). When these cells were treated with 2 µM of FK506 the cellular level of collagen α1(I) and α2(I) polypeptides decreased slightly compared to the control cells (Fig. 1A, top panel). However, secretion of collagen into cellular medium was profoundly affected. Fig. 1A, middle panel, shows a dramatic decrease in secretion of both collagen polypeptides from cells treated with 2 µM of FK506. At 1 µM FK506 was ineffective. The analysis of fibronectin secretion showed no change, indicating that the FK506 did not affect the general protein secretion machinery. Since FK506/FKBP3 may play a role in transcription regulation [26], we tested the expression of collagen α1(I) (COL1A1) and α2(I) (COL1A2) mRNAs (Fig. 1A, lower panel). We extracted the RNA from the same cells shown in the top and middle panels of Fig. 1A and analyzed collagen mRNAs by a semi-quantitative RT-PCR. There was no change in expression of collagen mRNAs with FK506 treatment, suggesting that FK506 had not altered transcription of collagen genes or stability of collagen mRNAs.

To extend these findings to hepatic stellate cells (HSCs), we isolated primary HSCs from rat livers and cultured them in vitro [40]. HSCs spontaneously activate after 2 days in culture and differentiate into activated HSCs by day 8, when they up-regulate collagen synthesis by 100 fold [6], [46]. At day 8 after isolation we treated rHSCs with 75 nM FK506 overnight. Then, we collected the cells and analyzed collagen protein expression in cellular extracts (Fig. 1B, top panel) and the medium (Fig. 1B, middle panel). The cellular level of collagen α1(I) polypeptide remained unchanged with the FK506 treatment. As control for loading we analyzed tubulin, because its expression does not change during HSCs activation, and α-smooth muscle actin (αSMA), which is the maker of HSC activation [46]. Expression of both these proteins was also not changed by FK506 treatment. We could not analyze the α2(I) polypeptide in these experiments, because there is no antibody available that can specifically recognize rodent α2(I) polypeptide. However, when we analyzed collagen excreted in the medium, FK506 treatment significantly decreased the secretion of collagen α1(I) polypeptide (Fig. 1B, middle panel). The secretion of fibronectin was not affected, again indicating that general excretion machinery was intact. When the steady state level of collagen mRNAs was analyzed (Fig. 1B, lower panel), no changes in expression was seen, suggesting that FK506 affects the ability of rHSCs to excrete type I collagen.

To verify this result in fully activated human hepatic stellate cells (hHSC) we cultured immortal human hepatic cell line [39] and treated the cells with 75 nM and 2 µM FK506 for 24 h. The cells were analyzed as above for the cellular and medium levels of type I collagen. Even at high FK506 concentration of 2 µM, the cellular level of collagen and αSMA was unchanged (Fig. 1C, upper panel). At 2 µM FK506 significantly inhibited excretion of collagen α1(I) polypeptides into the medium, while at 75 nM FK506 was ineffective (Fig. 1C, middle panel). Analysis of collagen mRNAs showed no change in expression (Fig. 1C, lower panel), again suggesting a defect in secretion of collagen polypeptides and not in collagen gene expression.

From these experiments we concluded that FK506 at doses of 2 µM in HLF and hHSCs and at 75 nM in rat HSCs significantly reduces the secretion of type I collagen. Since secreted type I collagen is relevant for fibrilogenesis, this result justified further evaluation of the antifibrotic potential of FK506.

### FK506 reduces type I collagen synthesis in liver slices cultured in vitro

**Figure 2:**
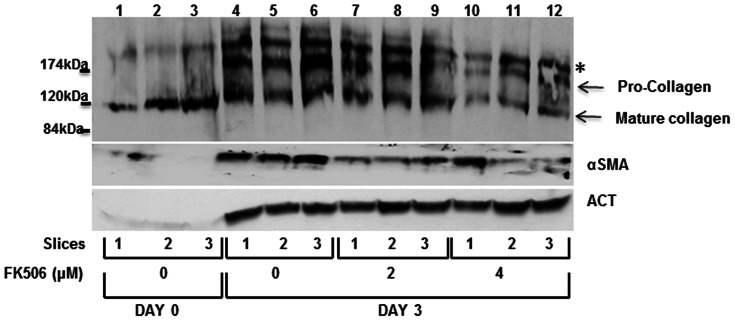
FK506 reduces collagen synthesis by precision cut liver slices. The 350 µm thick rat liver slices were analyzed immediately after the preparation (day 0, lanes 1–3) or after culturing for 3 days without FK506 (day 3, lanes 4–6) or with two different concentrations of FK506 (day 3, lanes 7–12). Expression of collagen α1(I) polypeptide, αSMA and actin, as a loading control, was analyzed by western blot. α1(I) collagen polypeptide is resolved as processed, mature, polypeptide of 120 kDa (mature collagen) and as freshly synthesized unprocessed α1(I) pro-peptide of 180 kDa (pro-collagen). A nonspecific band is indicated by asterisk.

Precision cut liver slices have been used before as in vitro model of fibrosis in the whole liver [42], [43]. When cut 250–350 microns thick and incubated in vitro for 2–3 days, liver slices initiate fibrosis by upregulating type I collagen expression. We employed this model to further test the FK506 effects on collagen synthesis, in a setting more relevant to hepatic fibrosis. The experiments were done using three separate slices treated independently for 3 days. Immediately after slicing (day 0), three slices were collected for analysis of the starting level of collagen expression. Other slices were incubated for 3 days in the presence of 2 µM and 4 µM of FK506 or vehicle (DMSO). The medium was changes daily and the drug was freshly added. At day 3, the slices were homogenized and total protein was extracted and analyzed by western blot for expression of collagen α1(I) polypeptide (Fig. 2). Freshly prepared slices (day 0) contained only the already existing type I collagen in the liver, which was detected as 120 kDa, mature, processed α1(I) polypeptide (Fig. 2, lanes 1–3). No active fibrilogenesis was detected, based on the absence of the newly synthesized pro-collagen of 180 kDa. After 3 days of culturing, the control slices contained high levels of pro-collagen molecular species, indicating active, *de novo* collagen synthesis (lanes 4–6). When treated with 2 µM of FK506 these slices had a similar level of pro-collagen (lanes 7–9) as control slices. However, the slices incubated in presence of 4 µM of FK506 had about 2-fold reduced levels of pro-collagen (lanes 10–12). RNA analysis of the slices showed no changes in collagen α1(I) and α2(I) mRNA expression (data not shown). As control for protein loading in Western blots we analyzed the actin levels. The absence of actin signal in slices at day 0 is an artifact of extraction, because actin filaments became insoluble and cannot be extracted when the liver and the slices are kept on ice (a necessary procedure in preparation of slices). However, the actin signal in other samples showed comparable loading. We also measured αSMA expression, as an indicator of activation of HSCs. It was highly increased in control slices after 3 days of incubation (lanes 4–6), compared to slices at day 0, suggesting rapid activation of HSCs in this model. FK506 at both concentrations slightly reduced the αSMA expression (lanes 7–12). However, it reduced collagen expression only at the higher concentration. These results suggested that FK506 can suppress activation of collagen synthesis in the liver and may be protective against hepatic fibrosis in vivo. Therefore, we proceeded to verify this in an animal model.

### Antifibrotic effect of FK506 in an alcohol model of hepatic fibrosis

Feeding 5% ethanol to Wistar rats for 4 weeks in combination with low doses of carbon tetrachloride (CCl_4_) injections is a well-established model of alcoholic fibrosis in rodents [45]. The use of CCl_4_ in combination with ethanol is necessary to achieve well developed fibrosis, but the animals show typical changes of alcoholic liver disease, like steatosis [45], [47]. To assess the potency of FK506 it was necessary to achieve fibrosis in all animals, with some animals showing advanced fibrotic changes.

**Figure 3:**
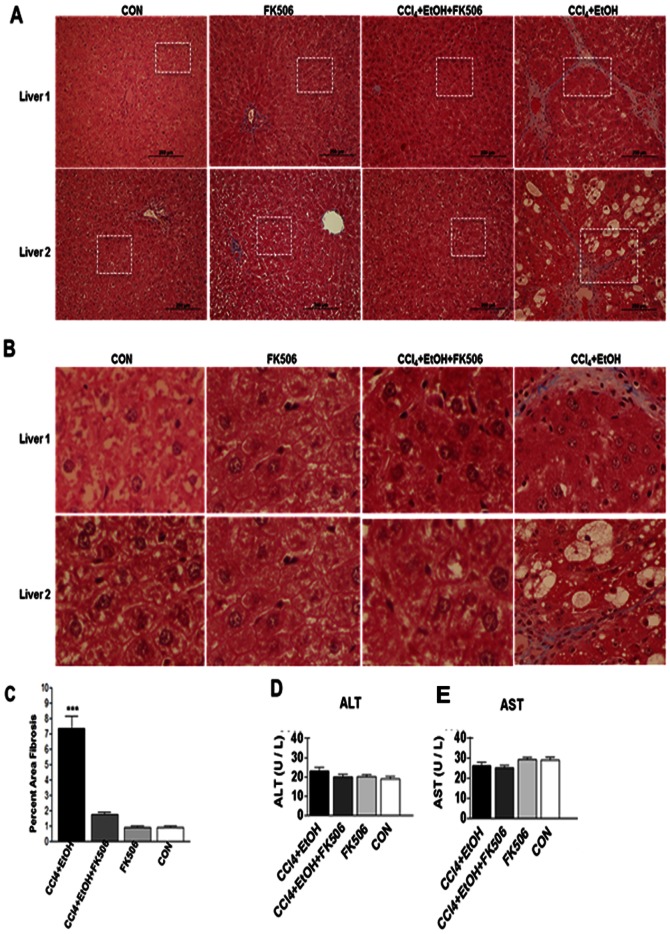
Antifibrotic effect of FK506 in alcohol model of hepatic fibrosis. A. Masson's trichrome staining of the liver sections from rats treated for 4 weeks as indicated. Two livers of each treatment group are shown at 100× magnification. B. 500× magnification of the squared sections in A. C. Percentage of liver fibrosis was determined from the Masson's trichrome staining using ImageJ software and plotted as percent fibrotic area vs. total liver area. Data represents average and±1 SEM from 8 rats, *** represents significance at p<0.01. D. and E. Aminotransferases in plasma of the experimental animals. The activity is presented as average and±1 SEM of 8 animals analyzed in duplicate.

Male Wistar rats (n = 32) weighing 150 – 200 grams and approximately 50 days of age were randomly divided into four groups. Group 1 (n = 8) was exposed to 5% ethanol in the drinking water at will, as the only source of water. In addition, a biweekly intraperitoneal (i.p) injections of CCl_4_ at 0.5 µL/g in mineral oil were performed (CCl_4_+EtOH group); Group 2 (n = 8) received the identical treatment, but with daily administration of FK506 (4 mg/g i.p.) from the day 1 (CCl_4_+EtOH+FK506 group); Group 3 (n = 8) received only FK506 daily (FK506 group), while group 4 (n = 8) received only vehicle (CON group). Daily liquid intake was measured and there was no significant difference between the groups in the total volume consumed (data not shown). After 28 days (24 hours after the last injection) liver samples and plasma were collected for analysis. The histology of two representative livers from each group stained with Mason's trichrome is shown in figure 3. Fig. 3A shows 100× magnification and Fig. 3B shows 500× magnification of the selected squared area. The CCl_4_+EtOH group developed moderate to advanced fibrosis with clearly visible bridging between adjacent portal tracts. In the CCl_4_+EtOH+FK506 group the fibrosis was completely absent and liver histology was similar to the CON group. Group which received only FK506 also did not show histopathological changes. The percentage of area of fibrosis was computed using the ImageJ software from all 8 trichrome stained slides of each group and plotted in figure 3C. The area of fibrosis in the CCl_4_+EtOH group was estimated to be 8.5% of the total liver area. In the CCl_4_+EtOH+FK506 group it was ∼2%, what represents a highly significant decrease of fibrosis (p<0.01). It was only slightly greater than the trichrome positively stained area in the CON group and FK506 group (∼1.2%) and this was not statistically significant. This result clearly indicated that FK506 was highly potent in preventing development of liver fibrosis in the alcohol/CCl_4_ model.

We also measured the level of amino-transferases in the plasma. The levels of ALT and AST were within the normal range in all groups averaging 20±2 U/L for ALT and 30±3 U/L for AST (Fig. 3D and E). This suggested that no significant necrosis accompanied fibrosis in our model, what was further verified by H&E staining and evaluation of liver histology (not shown).

**Figure 4:**
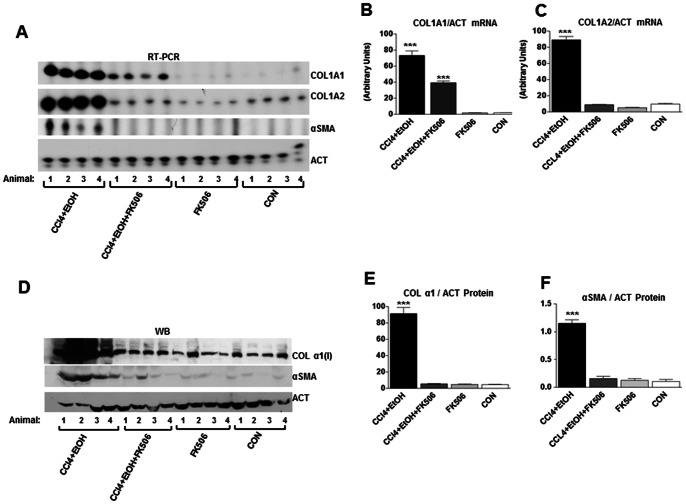
FK506 reduces expression of type I collagen and αSMA in hepatic fibrosis. A. RT-PCR analysis of collagen α1(I) mRNA (COL1A1), collagen α2(I) mRNA (COL1A2), αSMA mRNA and actin (ACT) mRNA in total liver RNA from the experimental animals. The results from 4 animals in each group are shown. B. and C. Quantification of expression of collagen α1(I) and α2(I) mRNA after normalization to actin mRNA expression. The data from all 8 animals were used and presented as average and±1 SEM. *** represents significance at p<0.01. D. Western blot analysis of total proteins from the livers of experimental animals. Collagen α1(I) polypeptide (COL α1(I)), αSMA and actin (ACT), as a loading control, from 4 animals of each group is shown. E. Quantification of expression of collagen α1(I) polypeptide after normalization to actin expression. The data from all 8 animals were used and presented as average and±1 SEM. *** represents significance at p<0.01. F. Quantification of αSMA expression after normalization to actin expression.

To further validate the FK506 efficacy, we analyzed type I collagen protein and mRNA expression in the liver samples by western blots and RT-PCR. All eight livers from each group were analyzed and used in statistical evaluation of these biochemical parameters (Fig. 4B and C), but we show the raw data of the four livers from each group (Fig. 4A and D). Fig. 4A shows the expression of collagen α1(I) and α2(I) mRNAs, αSMA mRNA and actin mRNA, as a loading control. The CCl_4_+EtOH livers had about 50-fold increased level of collagen α1(I) and α2(I) mRNAs, compared to CON and FK506 groups (for quantification see Fig. 4B and C). αSMA mRNA expression was clearly detected in this experimental group, but was absent in all other groups. This indicated activation of HSCs and massive up-regulation of collagen mRNA expression, what correlated well with the histology (Fig. 3). In the CCl_4_+EtOH+FK506 livers, expression of collagen α1(I) mRNA was decreased about two fold compared to the CCl_4_+EtOH livers, but it was still higher than that in CON and FK506 groups (Fig. 4A and B). However, the expression of collagen α2(I) mRNA in the CCl_4_+EtOH+FK506 group was completely suppressed and no different compared to CON and FK506 livers (Fig. 4D and E). This indicated that FK506 treatment failed to completely inhibit expression of collagen α1(I) mRNA, but abolished expression of collagen α2(I) mRNA, as well as development of fibrosis (Fig. 3). Expression of αSMA mRNA was undetectable in the CCl_4_+EtOH+FK506 livers, indicating that FK506 treatment also inhibited activation of HSCs. Alternatively; it may have reduced the number of HSCs in the livers. This result is similar to the result obtained with liver slices, where FK506 also decreased the expression of this marker of HSCs activation (Fig. 2).

Because of the failure of FK506 to completely suppress collagen α1(I) mRNA, in spite of preventing activation of HSCs and fibrosis, we measure the level of collagen α1(I) polypeptide in the livers by western blot (Fig. 4D and E). While collagen α1(I) polypeptide was highly up-regulated in the CCl_4_+EtOH livers, its expression in the CCl_4_+EtOH+FK506 livers was not up-regulated and was similar to that of CON and FK506 livers (Fig. 4E). This is consistent with absence of fibrosis in the CCl_4_+EtOH+FK506 livers, but does not correlate with the expression of its mRNA, which was still 25 fold higher than in CON and FK506 livers. The discrepancy between the mRNA level and protein level suggested that either, the α1(I) mRNA was not translated, or the α1(I) polypeptide was rapidly degraded.

The expression of αSMA protein was high in all CCl_4_+EtOH livers and significantly decreased in CCl_4_+EtOH+FK506 livers (Fig. 4D and F). Thus, by measuring αSMA protein, we could also demonstrate that FK506 treatment inhibited activation of HSCs or reduced their number in the liver. Based on these results we concluded that FK506 has multiple effects in the liver; which may include impaired translation of collagen α1(I) mRNA and inhibition of general activation of HSCs.

**Figure 5:**
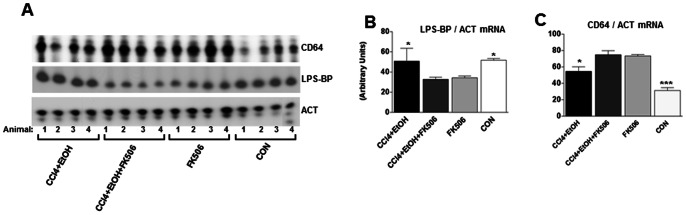
Expression of LPS-BP mRNA and CD64 mRNA in the livers of experimental animals. A. Total RNA from the livers of treated animals was analyzed by RT-PCR for expression of lipopolysaccharide binding protein mRNA (LPS-BP) and CD64 mRNA. Data from 4 animals of each group are shown. Loading control, actin (ACT). B. Quantification of expression of LPS-BP mRNA after normalization to actin mRNA expression. The data from all 8 animals were used and presented as average and±1 SEM. * represents significance at p<0.05. C. Quantification of expression of CD64 mRNA after normalization to actin mRNA. *** represents significance at p<0.01 and * represents significance at p<0.05.

Chronic inflammation under lays many fibrotic processes [1], therefore, it is possible that immunosuppressive activity of FK506 may have contributed to prophylaxis against fibrosis [48]. H&E staining showed no infiltration of lymphocytes, plasma cells and neutrophils in any of the livers. We also assessed the biochemical markers of Kupffer cells activation and analyzed the expression of TNF- α, IL1β, IL6, lipopolysaccharide binding protein (LPS-BP), CD64 and CD68. TNF-α, IL1β and IL-6 are the cytokines most commonly associated with liver inflammation, while CD64 and CD68 are markers of Kupffer cell activation [49], [50]. LPS-BP is binding protein for LPS, which presents LPS to its receptor [51], [52]. It is of particular importance, because alcoholic liver injury is mediated by increased absorption of LPS from gut flora and stimulation of Kupffer cells by LPS [53]. Expression of TNF- α, IL1β, IL6, CD64 was undetectable by RT-PCR in the livers of all groups (data not shown). When CD64 was analyzed, its expression was variable between the groups, but there was no clear correlation between the CD64 expression, fibrosis and FK506 treatment (Fig. 5A and C). The expression of LPS-BP was down-regulated in the FK506 and CCl_4_+EtOH+FK506 groups, suggesting that FK506 can alter the expression of this mediator of Kupffer cell activation. However, the expression of LPS-BP was similar in the CCl_4_+EtOH and CON groups (Fig. 5A and B), suggesting that it probably did not play a major role in the pathogenesis in our model. We concluded from these experiments that our fibrosis model was not associated with overt inflammation and that it is not likely that immunosuppressive action of FK506 is responsible for its dramatic antifibrotic effect.

### LARP6 dependent mechanism of collagen synthesis as a target of FK506

**Figure 6:**
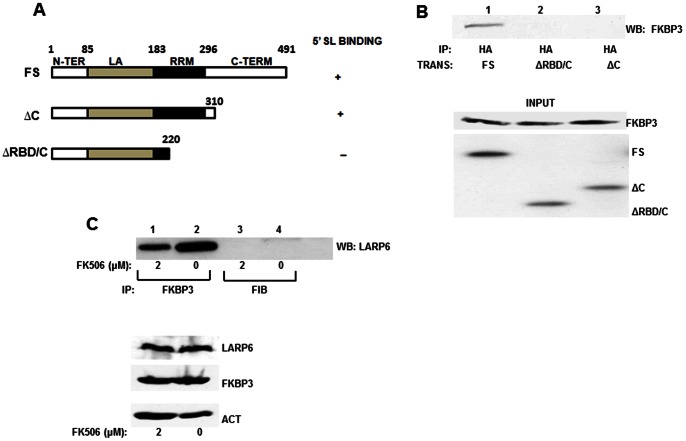
Interaction of LARP6 with FK506 binding protein 3 (FKBP3). A. Schematic representation of LARP6 constructs used in immunoprecipitations (IP) with amino-acid numbering on the top. All constructs contained HA tag at the N-terminus. Full size LARP6 (FS) has the domains indicated: N-TER, N-terminal domain, La, La homology domain, RRM, RNA recognition motif, C-TER, C-terminal domain. Binding of the constructs to 5′ SL RNA is indicated to the right. B. IP of endogenous FKBP3 with LARP6 constructs. Upper panel: after expression of HA-tagged LARP6 constructs in HEK293 cells, IP was performed with anti-HA antibody and Western blot with anti-FKBP3 antibody. Bottom panel: Expression of the proteins in the input material analyzed by Western blot. C. IP of endogenous LARP6 with endogenous FKBP3 in cells treated with FK506. Top panel: HLFs were treated with the indicated concentrations of FK506. The IP was done using anti-FKBP3 antibody (lanes 1 and 2) or anti-fibronectin antibody (FIB, lanes 3 and 4), as control, and Western blot probed with anti-LARP6 antibody. Bottom panel: expression of proteins in the input material.

LARP6 is the protein that binds 5′SL of collagen mRNAs [18]. It has been implicated in regulation of stability and translation of collagen mRNAs [19], [20], [22]. Using yeast-two-hybrid screening we cloned FKBP3 as one of the proteins that interacts with LARP6. This approach also indicated that the interaction between these two proteins is direct (in preparation). Since FKBP3 binds FK506 [54], this raised a possibility that this drug may interfere with LARP6/FKBP3 interaction, as one of the mechanisms by which it could suppress collagen synthesis. To test this hypothesis we first verified the interaction of LARP6 and FKBP3. To map the interaction domain on LARP6 we designed adenoviruses expressing different mutants of LARP6 (shown in Fig. 6A) and analyzed these constructs for the ability to interact with endogenous FKBP3. By immunoprecipitation (IP) of LARP6 and western blot analysis for FKBP3 pull down (Fig. 6B) we demonstrated that FKBP3 only immunoprecipitated with the full size LARP6. LARP6ΔC, a mutant which lacks the C-terminal domain, but still can bind 5′SL [18], [22], showed no interaction with FKBP3. Even shorter construct, LARP6ΔC/RBD, also did not interact with FKBP3, so we concluded that FKBP3 binding to LARP6 requires the presence of the C-terminal domain of LARP6. Detailed LARP6/FKBP3 interaction and its function is currently under investigation (manuscript in preparation).

To test if FK506 may inhibit the interaction of endogenous LARP6 and FKBP3 we treated the HLFs with 2 µM of FK506, the concentration which reduced collagen synthesis in these cells (Fig. 1). Similar experiment in HSCs was not possible due to lower expression of LARP6 in these cells compared to HLFs. In the presence of FK506, the pull down of LARP6 with anti-FKBP3 antibody was reduced by ∼50% (Fig. 6C, lane 1), compared to the control pull down (lane 2). The control reactions using anti-fibronectin antibody or no antibody did not pull down any LARP6 (lanes 3 and 4). The expression of proteins in the input material was similar (Fig. 6C, lower panel). This suggested that FKBP3 may interfere with the direct binding of LARP6 to FKBP3. To verify that this interaction is not cell type specific we repeated FKBP3/LARP6 pull downs in scleroderma skin fibroblasts with the same result (data not shown).

**Figure 7:**
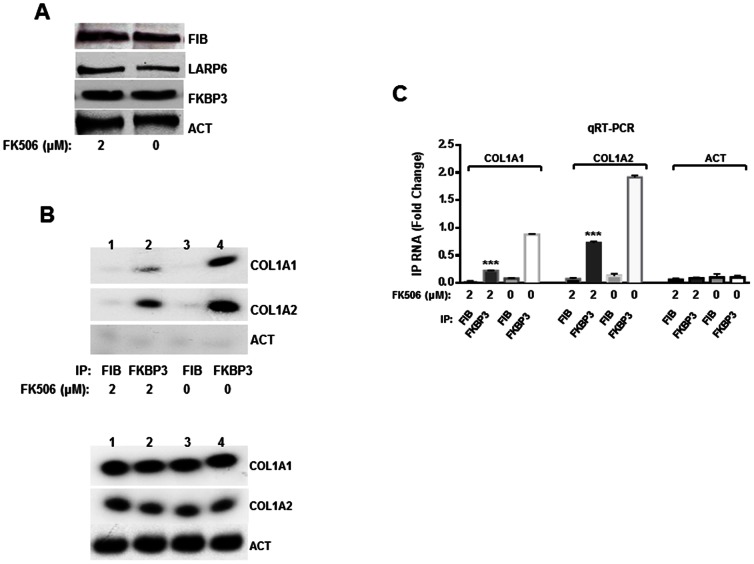
FK506 inhibits association of collagen mRNAs with FKBP3. A. Expression of proteins in the input material used for mRNA pull downs analyzed by Western blot. B. Top panel: pull down of collagen mRNAs with FKBP3. HLFs treated with 2 µM FK506 (lanes 1 and 2) or untreated HLFs (lanes 3 and 4) were used for IP with anti-FKBP3 antibody (lanes 2 and 4) or anti-fibronectin antibody (lanes 1 and 3). The IP material was analyzed for collagen α1(I) (COL1A1), collagen α2(I) (COL1A2) and actin (ACT) mRNA by semi-quantitative RT-PCR. Bottom panel: analysis of the mRNAs in the input material. C. Quantitative RT-PCR (qRT-PCR) analysis of the collagen mRNAs in the IP from A. The experiments were done in duplicates and plotted as fold change over the negative control, actin mRNA. The error bars represent average with±1 SEM. *** represents significance with p<0.01.

FKBP3 interaction to collagen mRNAs is mediated by LARP6 and this interaction was attenuated by FK506 (Fig. 6). This raises a possibility that FK506 may interfere with the function of LARP6/FKBP3 complex when bound to collagen mRNAs. Therefore, we tested the interaction of FKBP3 with collagen α1(I) and α2(I) mRNAs in the presence or absence of FK506. These experiments were done using human lung fibroblasts (HLFs), which express high level of endogenous LARP6, allowing the analysis of the interaction of the endogenous proteins [18]. The cells were treated with 2 µM of FK506 or DMSO (CON) for 24 h and immunoprecipitated with anti-FKBP3 or control anti-fibronectin antibodies. After the immunoprecipitation, RNA was extracted and analyzed by RT-PCR for pull down of collagen α1(I) and α2(I) mRNAs (Fig. 7B). Collagen α1(I) and α2(I) mRNAs were efficiently immunoprecipitated with anti-FKBP3 antibody (Fig. 7B lane 4), while there was no immunoprecipitation with the control (anti-fibronectin) antibody (Fig. 7B lanes 1 and 3). Actin mRNA was not pulled down with either antibody, suggesting that the interaction of collagen mRNAs with FKBP3 is specific for this mRNA. Since FKBP3 has not been shown to bind any RNA, and it does not bind collagen mRNAs without the presence of LARP6 (in preparation), the pull down of collagen mRNAs was almost certainly mediated by interaction of FKBP3 and LARP6. However, when HLFs were treated with FK506, the collagen pull down was reduced 2–3 fold (Fig. 7B lane 2). The amount of proteins (Fig. 7A) and collagen mRNAs (Fig. 7B) in the input material was similar, suggesting that the strength of interaction between FKBP3 and LARP6 was altered. To better quantify the results shown in figure 7B, we re-analyzed the samples from the immunoprecipitation reaction by real time RT-PCR (Fig. 7C). The quantitative RT-PCR was in excellent agreement with the semi-quantitative RT-PCR and verified that both, α1(I) and α2(I) mRNAs, were pulled down 50% less efficiently in FK506 treated HLFs. We concluded from these experiments that one of the mechanisms of antifibrotic activity of FK506 may involve inhibition of the interaction between LARP6 and FKBP3, resulting in aberrant translation of collagen mRNAs and inefficient folding and secretion of collagen polypeptides.

## Discussion

Post-transcriptional regulation of type I collagen is the major mechanism of excessive synthesis of type I collagen in fibrosis of various organs [15], [18]–[20], [22]. The mechanism probably operates in all collagen producing cells, including fibroblasts, myofibroblasts and HSCs [55]. To understand the molecular details of the mechanism which governs collagen synthesis we have previously identified and characterized a novel RNA binding protein, LARP6 [18]. In the 5′ UTR of collagen α1(I) and α2(I) mRNAs there is a unique sequence that can be folded into a stem-loop structure, the 5′SL [56]. LARP6 binds 5′SL of collagen mRNA and regulates coordinated translation of collagen α1(I) and α2(I) mRNAs [18]. LARP6 also interacts with several other proteins, including RNA helicase A, vimentin, nonmuscle myosin and STRAP [19], [20], [22]. Recently, we have discovered yet another protein that interacts with LARP6, FKBP3. Our preliminary results suggested that knock down of FKBP3 decreases expression of type I collagen (in preparation). Elucidation of the molecular mechanism of collagen synthesis is crucial for development of specific antifibrotic therapy. Since FKBP3 binds the well-established immunosuppressive drug, FK506 [57], we surmised that this compound may interfere with FKBP3 function in collagen synthesis. In previous studies, FK506 had both profibrotic [37], [38] and antifibrotic [36], [58] effects, based on the animal models used. In this study, we wanted to establish the effectiveness of FK506 in an alcohol related model and in addition, provide an insight into a novel mechanism of action of FK506.

Our results show that in vitro (Fig. 1) and in cultured liver slices (Fig. 2), FK506 reduced collagen expression. This was predominantly due to a reduced excretion of collagen into the cellular medium. In liver slices it also inhibited the activation of HSC, as judged by expression of the marker of activation, αSMA. In vivo, in an alcohol model of hepatic fibrosis, FK506 completely prevented development of fibrosis (Fig. 3). We employed a preventive model in this study; however, the potency of FK506 remains to be evaluated in a curative model. Based on our results, such study is highly warranted.

FK506 is thought to be nephrotoxic at higher doses [59]. Our dosage was 30% higher than the therapeutic recommendations in humans, but the treated animals did not show any overt renal impairment. One other drawback of chronic administration of FK506 is immunosuppression. The duration of our study was too short to observe any adverse effects on the immune system. The vast experience with this drug in human use will help adjust the dose to avert severe side effects if the drug reaches clinical trials for fibrosis.

What is the mechanism of action of FK506 in fibrosis? First, FK506 either prevented activation of HSCs in liver slices (Fig. 2) and in the animal model (Fig. 4) or reduced their numbers, without affecting the activation. The measurement of αSMA in total liver homogenates could not distinguish between these possibilities, but, based on the results with liver slices, we believe that it suppressed the activation of HSCs. However, it is clear that FK506 inhibited the interaction between LARP6 and FKBP3. Both of these proteins are critical for efficient collagen synthesis (in preparation and [18]). It also reduced the fraction of collagen mRNAs that can be immunoprecipitated with FKBP3 (Fig. 7). This indicates that coordinated translation of collagen mRNAs and efficient folding of collagen triple helix may have been perturbed. When collagen synthesis is impaired, it yields to unfolded collagen polypeptides accumulation in the ER that undergoes excessive post-translational modifications [21], [60]. The intracellular accumulation of hyper-modified polypeptides triggers the unfolded protein response and cell apoptosis [61], [62]. Such mechanism may have inhibited activation of HSCs and/or caused their elimination from the liver.

Second, we observed that FK506 had a different effect on collagen α1(I) mRNA and α2(I) mRNA. While the drug completely inhibited up-regulation of collagen α2(I) mRNA in the fibrotic livers (Fig. 4A), it reduced expression of collagen α1(I) mRNA two fold and this mRNA was still significantly increased compared to the control livers (Fig. 4A). Studies on the transcription rates of collagen α1(I) and α2(I) genes in HSCs and measurements of the stability of collagen mRNAs revealed a possible explanation for this phenomenon. Transcription rates of collagen α1(I) and α2(I) genes are increased to a similar extent, about 3 fold, in activated HSCs compared to quiescent HSCs [13], [63] and in fibrotic livers compared to control livers [64]. However, collagen α1(I) mRNA is stabilized by binding of protein αCP to the C-rich sequence in the 3′ UTR of this mRNA [13]. This binding takes place only in activated HSCs and not in quiescent HSCs [14] and prolongs the long half-life of this mRNA. A similar C-rich sequence is not found in collagen α2(I) mRNA, thus, an additional mechanism involving mRNA stabilization contributes to higher accumulation of collagen α1(I) mRNA in fibrosis. Therefore, the effect of FK506 on steady state level of collagen α1(I) mRNA was less pronounced. Nevertheless, the collagen α1(I) mRNA that accumulated to higher levels in CCl_4_+EtOH+FK506 livers was not efficiently translated, as evidenced by the levels of α1(I) polypeptide, which were similar to that in control livers (Fig. 4C). This is consistent with explanation that FK506 primarily disrupted LARP6 mediated translation of collagen mRNAs by blocking the FKBP3/LARP6 interaction.

Third, liver fibrosis is often accompanied by subclinical chronic inflammation [11]. Chronic ethanol consumption causes oxidative stress and promotes inflammation [9]. In addition, increased gut permeability of alcoholics allows increased absorption of LPS produced by the gut flora. LPS binds to LPS-BP and is presented to the receptors on Kupffer cells, this results in activation of Kupffer cells with up-regulation of the markers of Kupffer cells activation, CD64 and CD68 and production of cytokines such as TNF-α, IL-6 and IL1 [10], [11], [65]. Therefore, we measured the expression of these factors in the livers (Fig. 5), but could not detect any difference in expression between fibrotic vs. control livers. Histological examination did not reveal infiltration of any immune cells into the liver. We particularly looked for lymphocyte infiltration, because it is known that FK506 can suppress activation of T-lymphocytes [28], [48]. These findings suggested that the fibrosis model employed in this study is not associated with a detectable hepatic inflammation. However, it does not exclude the presence of subclinical pro-inflammatory changes, therefore, we cannot completely exclude that some of the FK506 antifibrotic effects are due to suppressing the immune response. However, we do not believe that this is the major mechanism of FK506 activity in the model employed.

Fourth, in addition to FKBP3, there are additional FK506 binding proteins (FKBPs) which bind FK506 with high affinity. They all possess peptidyl-prolyl isomerase activity (PPIase), which is inhibited by FK506 at the half maximal PPIase inhibitory concentration of 400 nM [57]. The general inhibition of prolyl bonds conversion in proteins may have contributed to the antifibrotic effect by limiting cis-trans-isomarization of the propyl-peptide bonds in collagen polypeptides. However, the general PPIase inhibition would affect all proteins and would be highly toxic to all cells, including hepatocytes. We have not observed any necrosis of hepatocytes, as evidenced by normal aminotransferase levels (Fig. 3D and E) and absence of necro-inflammatory changes in histology (not shown). Also, FK506 has been in clinical practice for years with well-established doses and side effects [30], [66], which exclude general cytotoxicity.

In conclusion, our study is first to provide an insight into the mechanism of antifibrotic effect of FK506. Our results support the hypothesis that FK506 perturbs synthesis of type I collagen by interfering with binding of FKBP3 to LARP6. This interaction is necessary for regulating the synthesis of heterotrimeric type I collagen and is currently studied in our lab. Impaired collagen synthesis may result in overloading of the ER with unfolded collagen polypeptides and apoptosis of collagen producing cells. Although encouraging, these are only preliminary results that need to be further tested in other models of hepatic fibrosis and with respect to translation of collagen mRNAs, unfolded protein response and apoptosis of HSCs. Lastly, we are not eliminating a possibility that FK506 may target additional PPIases that may play a critical role in proper collagen folding and excretion. We believe that this report will renew the interest of using FK506 as an antifibrotic drug.
