# Correction: Tacrolimus (FK506) Prevents Early Stages of Ethanol Induced Hepatic Fibrosis by Targeting LARP6 Dependent Mechanism of Collagen Synthesis

**DOI:** 10.1371/journal.pone.0306020

**Published:** 2024-06-20

**Authors:** Zarko Manojlovic, John Blackmon, Branko Stefanovic

In Fig 3B, the Liver 1 and Liver 2 panels for FK506 were erroneously duplicated. The Liver 1 image is correct. An updated version of Fig 3 is provided here in which the Liver 2 FK506 panel has been replaced. Underlying quantification data supporting Fig 3C from recent replicate analyses of the samples used in the original experiments are available in S2, S3, S4 and S5 Files. The original individual-level quantitative data from which Fig 3C were generated are no longer available.

In the ACT panel of Fig 5A, the bands in lane 16 (labelled as animal 4 of the CON group) were repositioned lower to align with the other lanes to correct for a slow gel migration artifact. An updated Fig 5A is provided here in which the ACT panel is shown without selective adjustment of lane 16. The original underlying gel for Fig 5A is provided in S1 File.

Underlying data for Figs 3A, 4A, and 7A are also provided here in S1 File. The remaining data for this article are available upon request from the corresponding author as per the data availability policy in place at the time of the article’s publication.

The *PLOS ONE* Editors apologize for the delay in resolving this matter.

The authors apologize for the errors in the published article.

**Fig 3:**
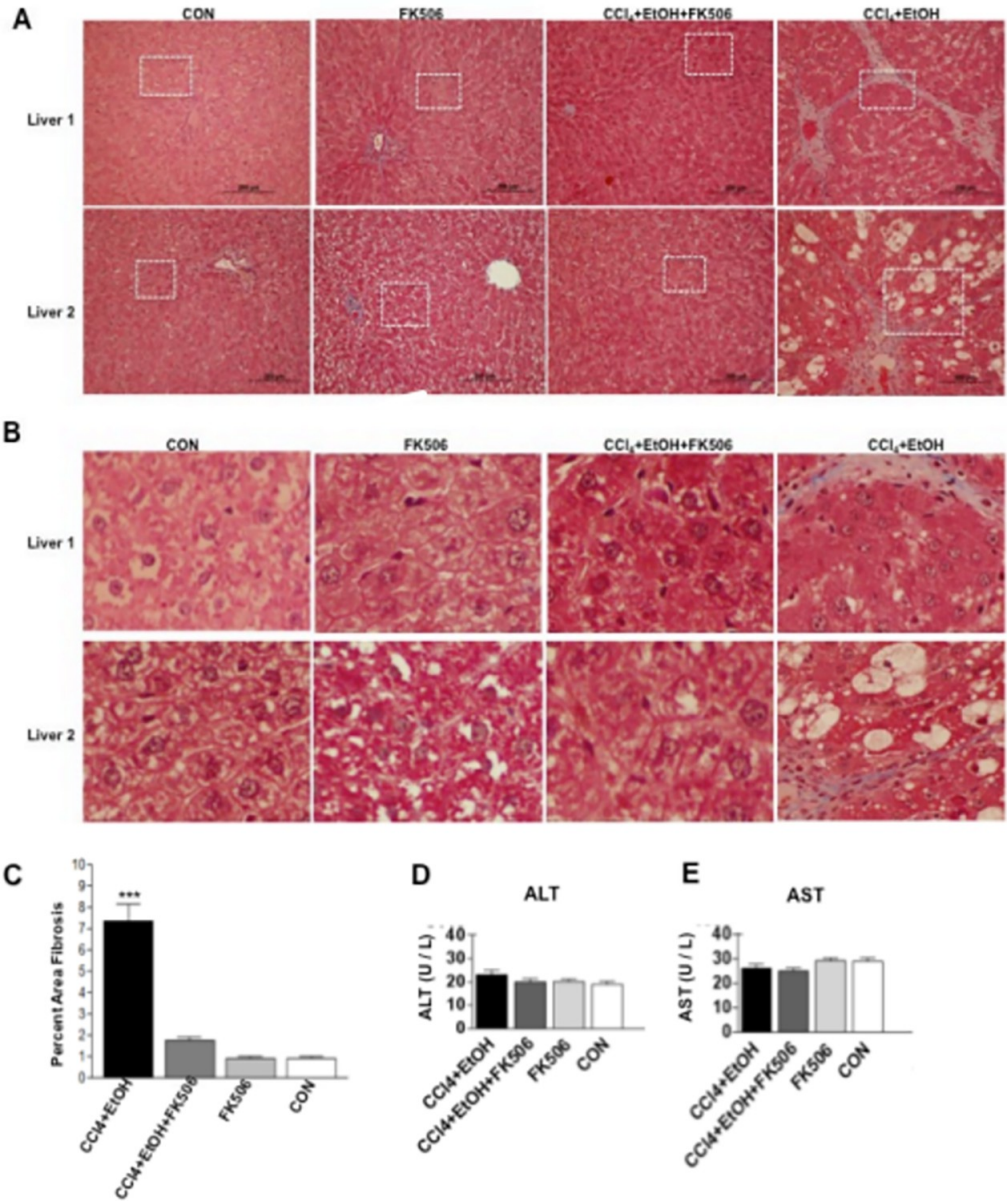
Antifibrotic effect of FK506 in alcohol model of hepatic fibrosis. A. Masson’s trichrome staining of the liver sections from rats treated for 4 weeks as indicated. Two livers of each treatment group are shown at 100× magnification. B. 500× magnification of the squared sections in A. C. Percentage of liver fibrosis was determined from the Masson’s trichrome staining using ImageJ software and plotted as percent fibrotic area vs. total liver area. Data represents average and±1 SEM from 8 rats, *** represents significance at p<0.01. D. and E. Aminotransferases in plasma of the experimental animals. The activity is presented as average and±1 SEM of 8 animals analyzed in duplicate.

**Fig 5:**
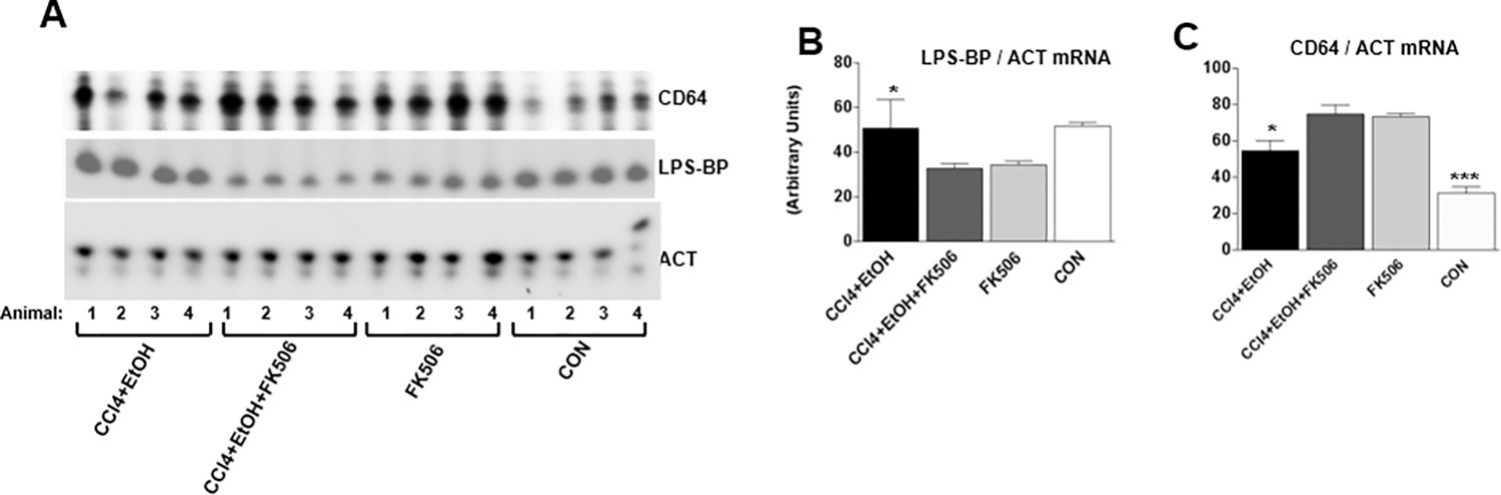
Expression of LPS-BP mRNA and CD64 mRNA in the livers of experimental animals. A. Total RNA from the livers of treated animals was analyzed by RT-PCR for expression of lipopolysaccharide binding protein mRNA (LPS-BP) and CD64 mRNA. Data from 4 animals of each group are shown. Loading control, actin (ACT). B. Quantification of expression of LPS-BP mRNA after normalization to actin mRNA expression. The data from all 8 animals were used and presented as average and±1 SEM. * represents significance at p<0.05. C. Quantification of expression of CD64 mRNA after normalization to actin mRNA. *** represents significance at p<0.01 and * represents significance at p<0.05.

## Supporting information

S1 File Image Data underlying Fig 5. .
(PDF)

S2 File Fig 3 Quantification part 1. (ZIP)

S3 File Fig 3 Quantification part 2. (ZIP)

S4 File Fig 3 Quantification part 3. (ZIP)

S5 File Fig 3 Quantification part 4 .
(ZIP)
