# Supplementary material for: Correction: Tacrolimus (FK506) Prevents Early Stages of Ethanol Induced Hepatic Fibrosis by Targeting LARP6 Dependent Mechanism of Collagen Synthesis
Source: PLoS One. 2024 Jun 20;19(6):e0306020. doi: 10.1371/journal.pone.0306020 (PMC11189226; doi:10.1371/journal.pone.0306020)
Supplement: S1 File — . (PDF) [file pone.0306020.s001.pdf]

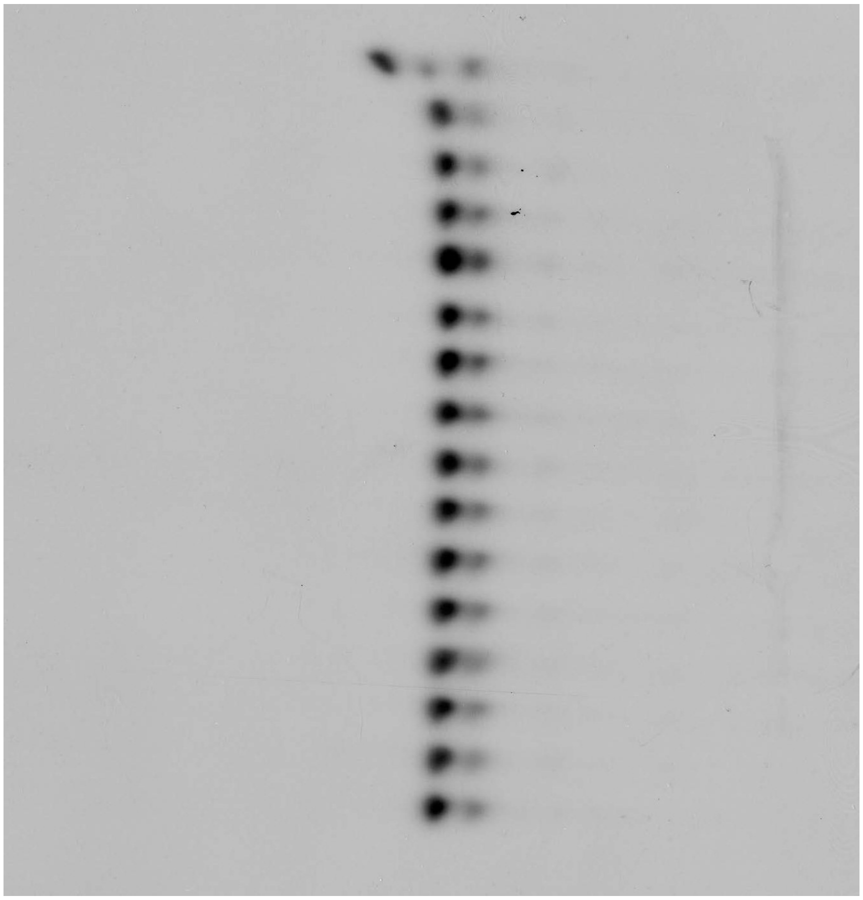

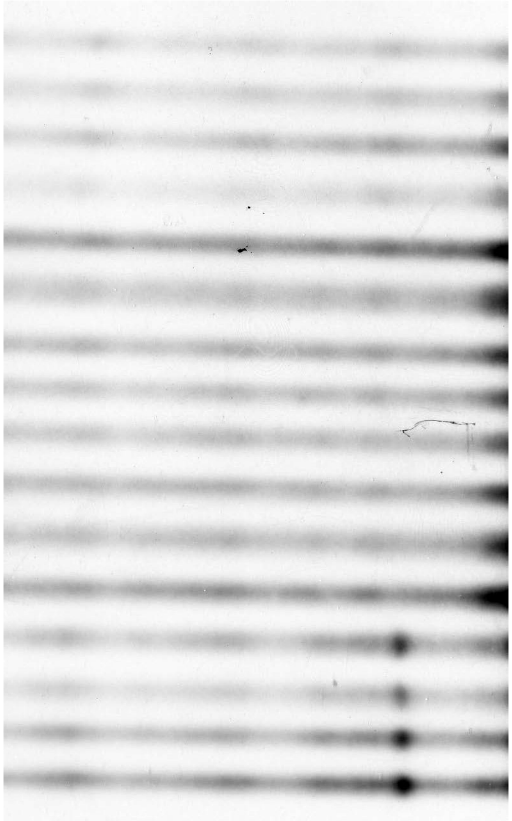

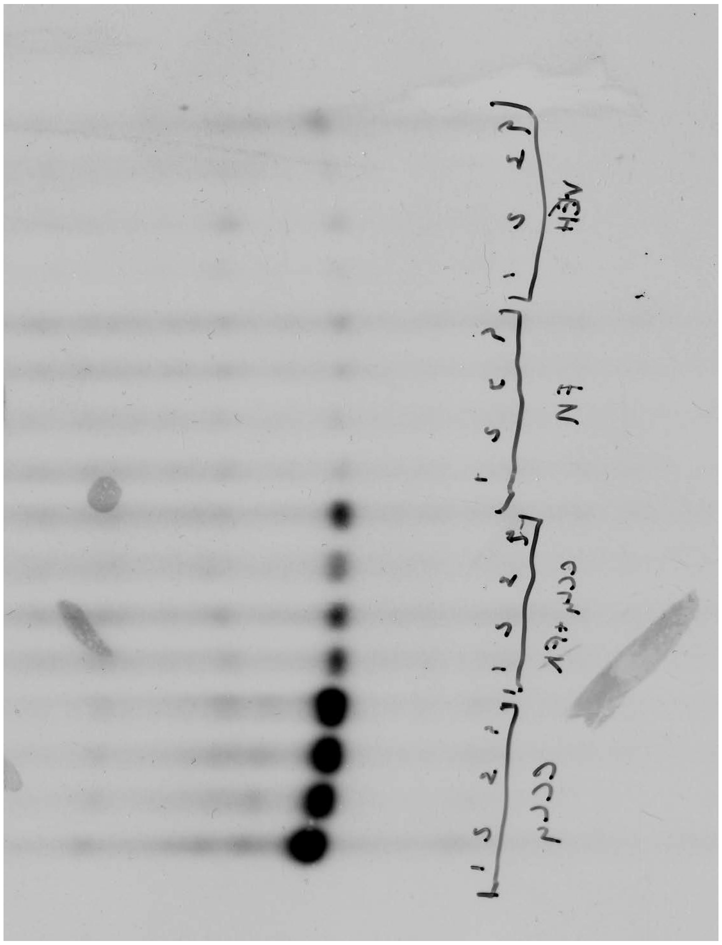

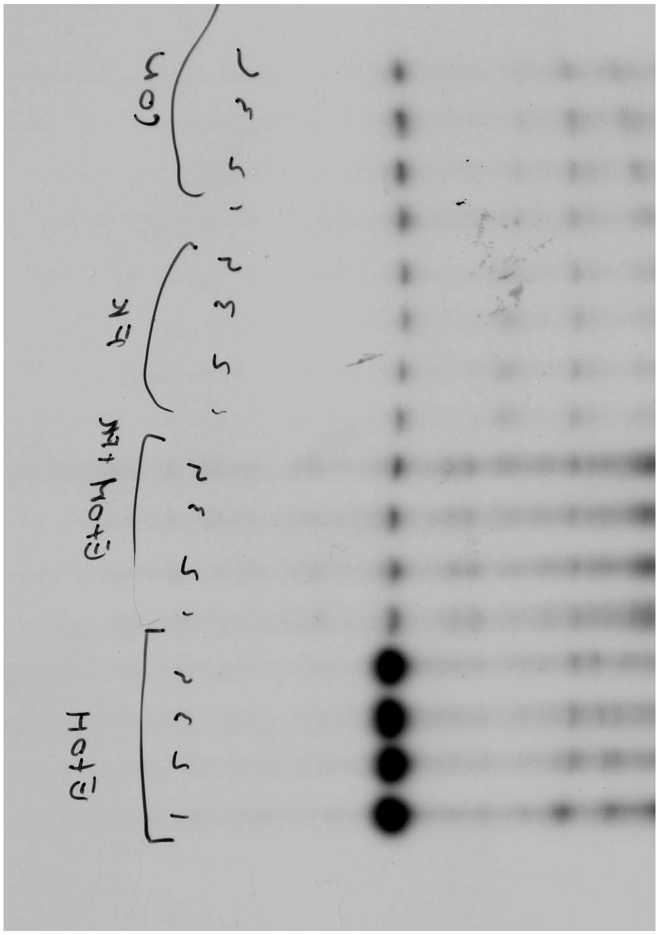

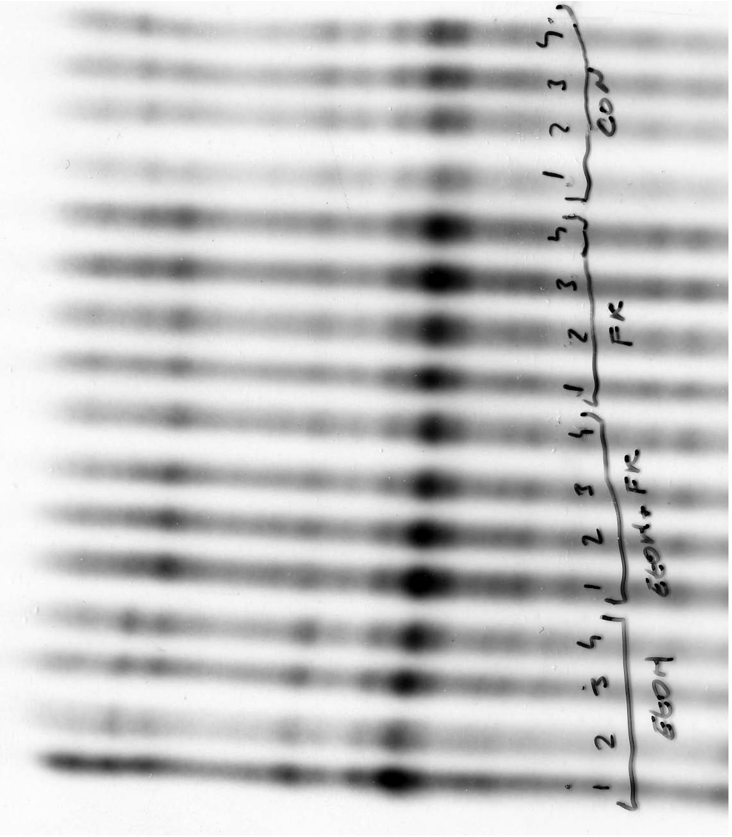

1 2 3 4
EtOH
1 2 3 4
EtOH + FK
1 2 3 4
FK
1 2 3 4
CON

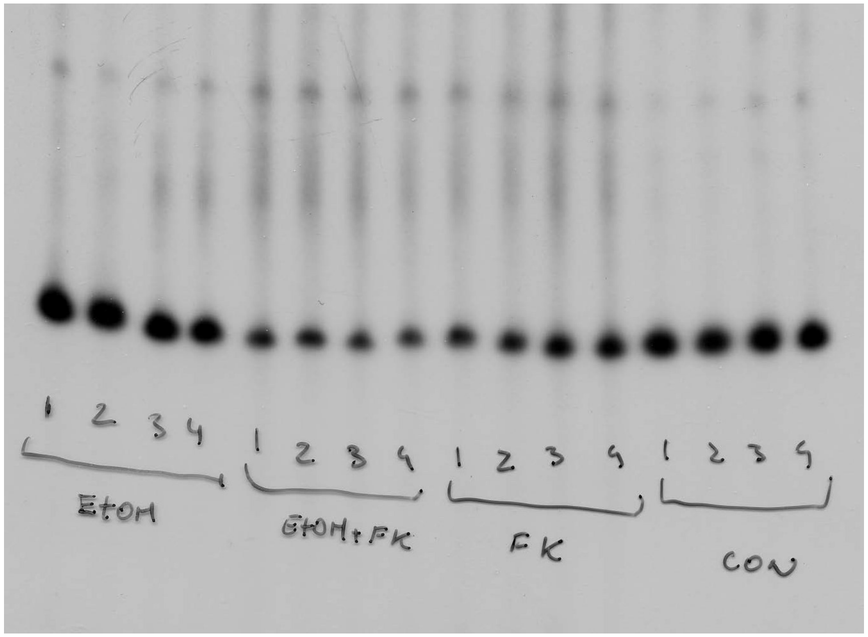

1 2 3 4
EtOH
1 2 3 4
EtOH+FK
1 2 3 4
FK
1 2 3 4
CON

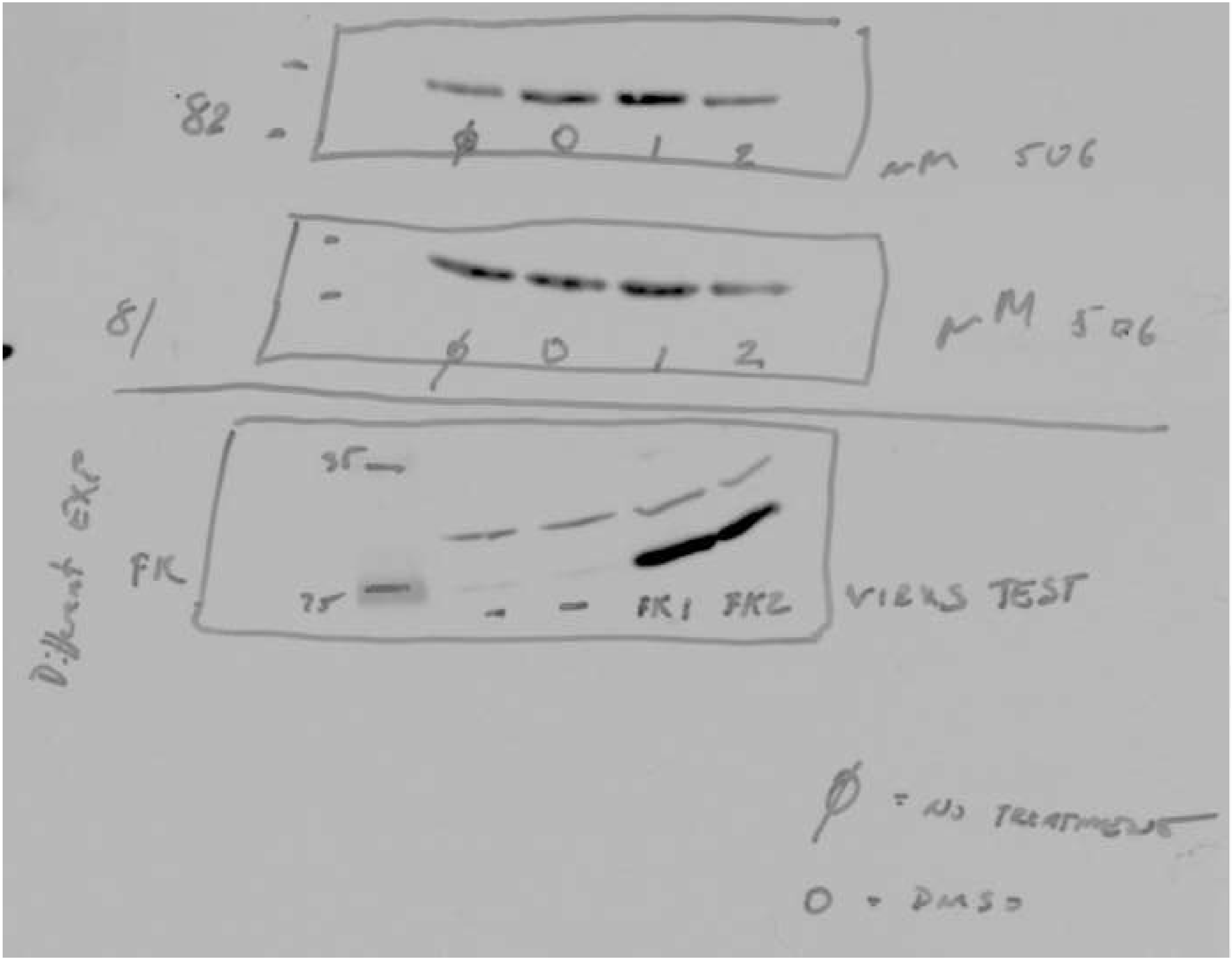

82
ø
O
1
2
nM 5U6
8/
ø
O
1
2
nM 5U6
Different exp
35
FK
75
FK1
FK2
VIERS TEST
ø = NO TREATMENT
O = DMSO

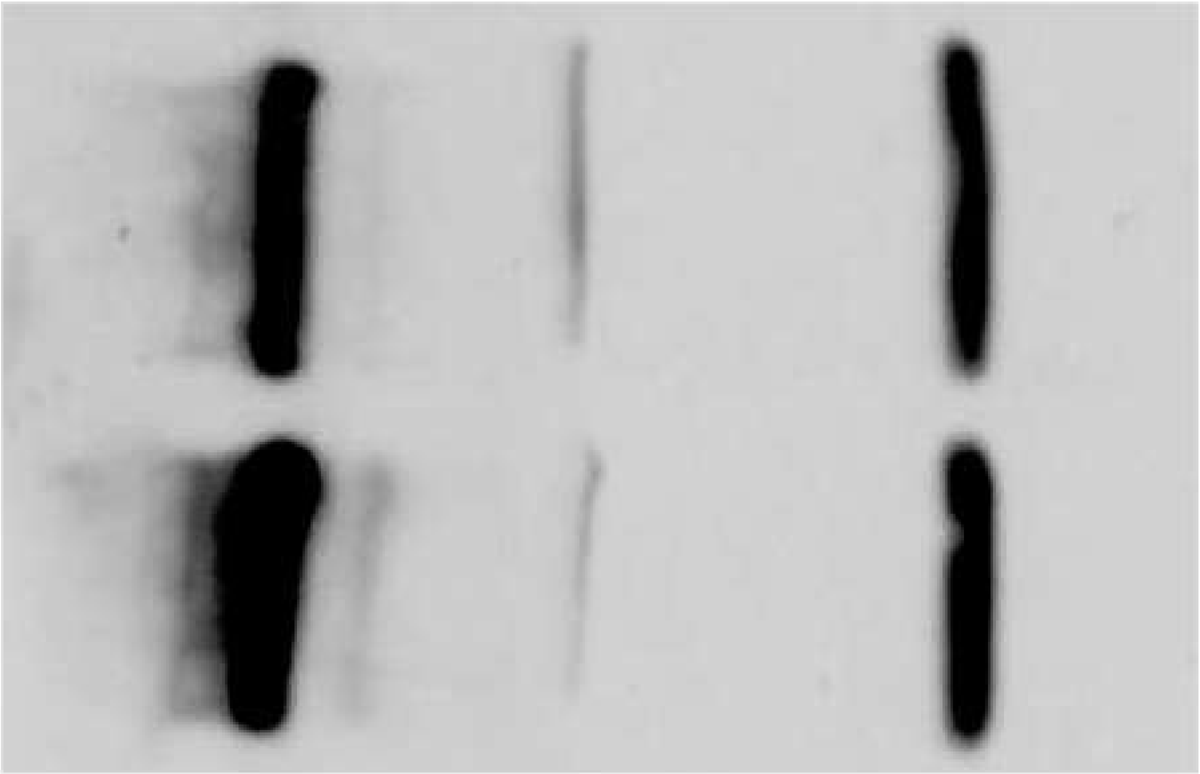

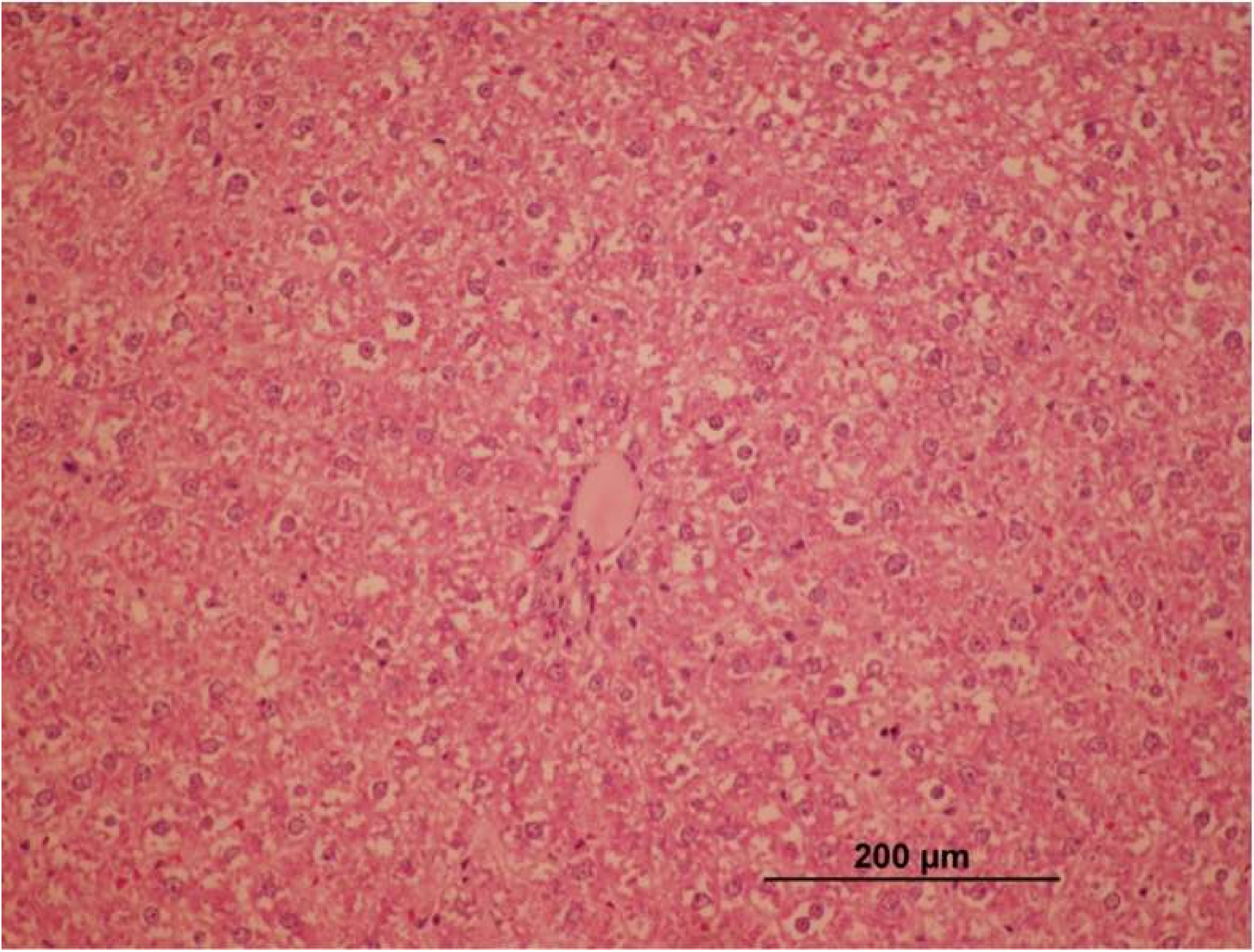
200 μm

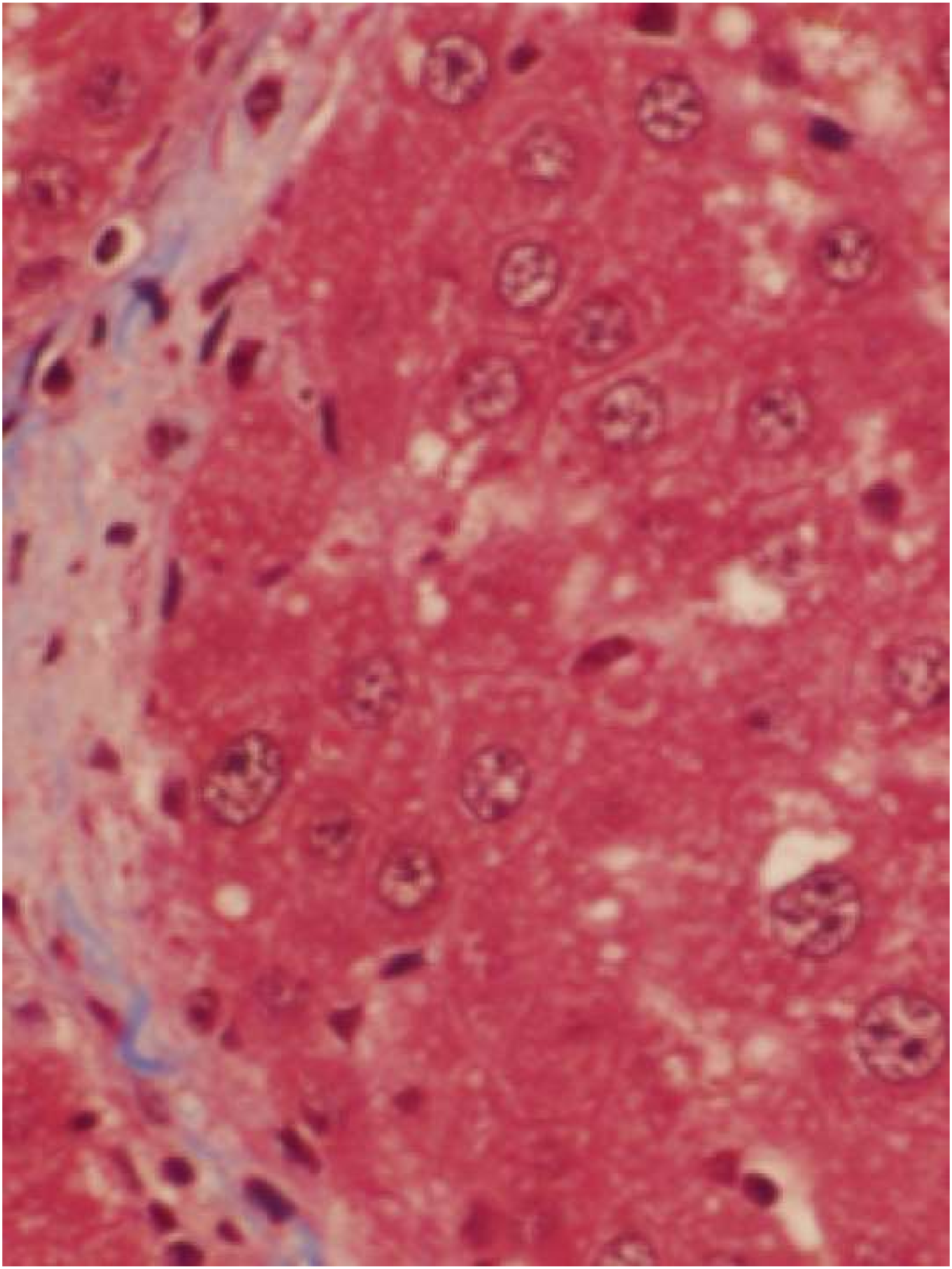

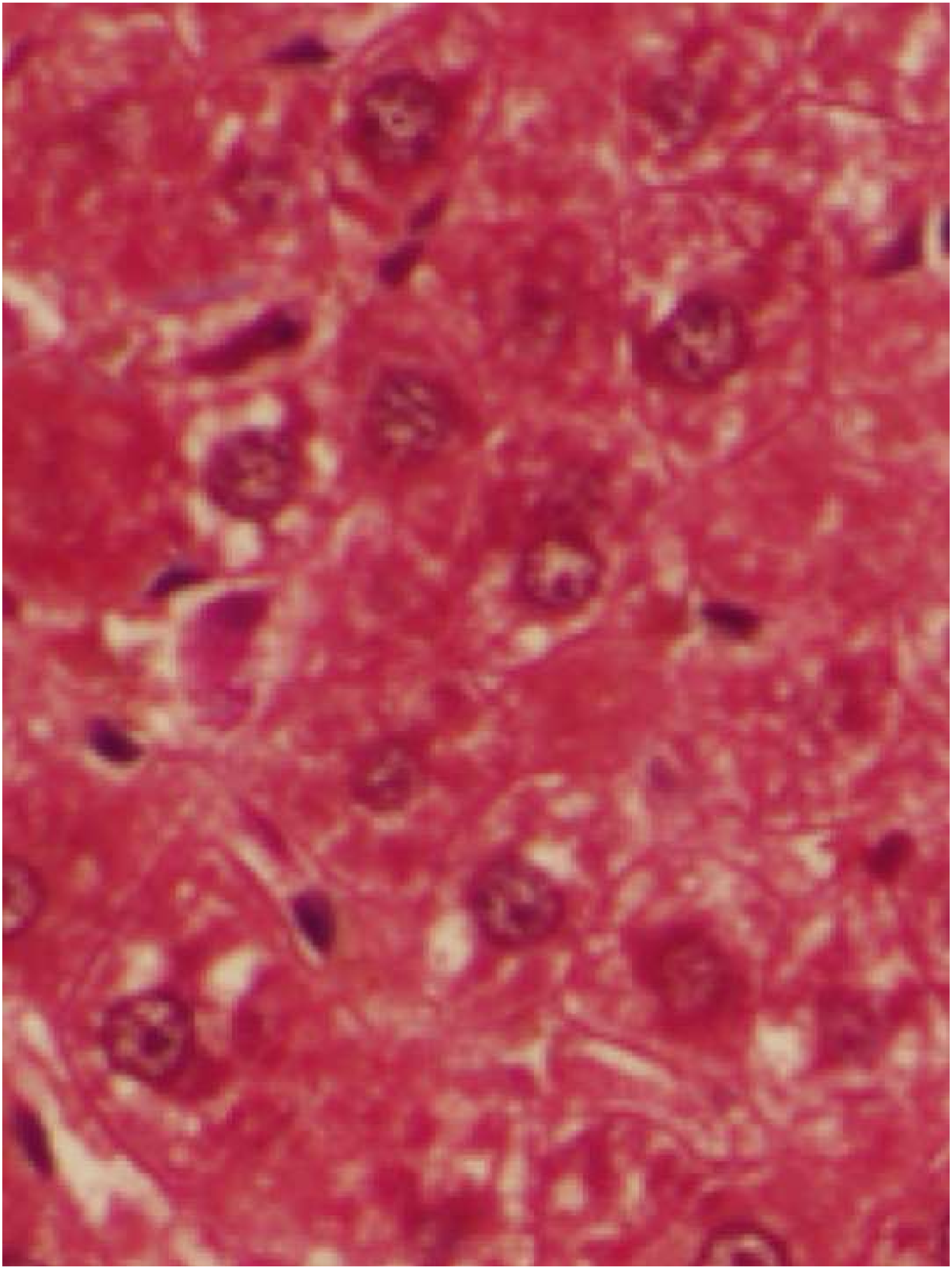

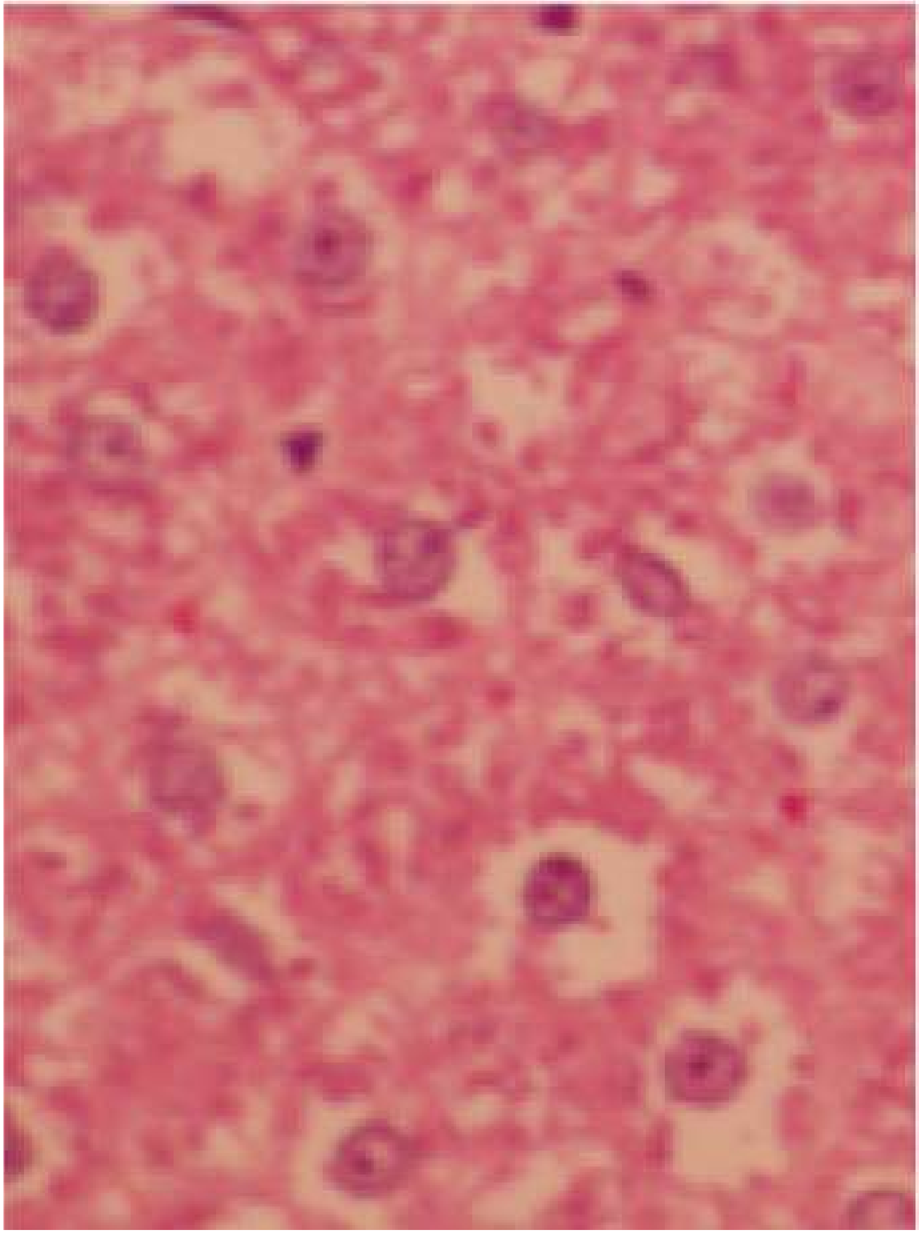

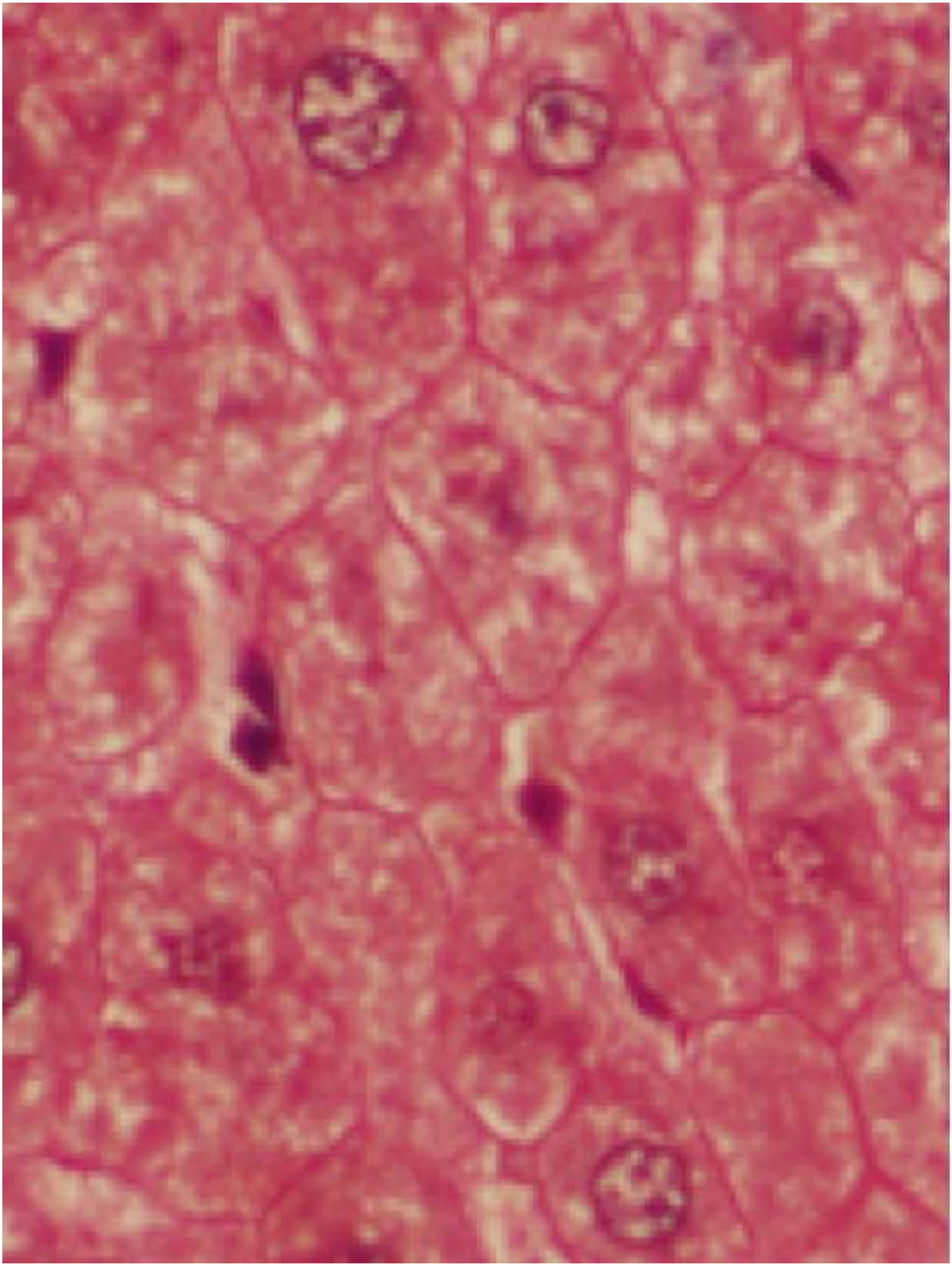

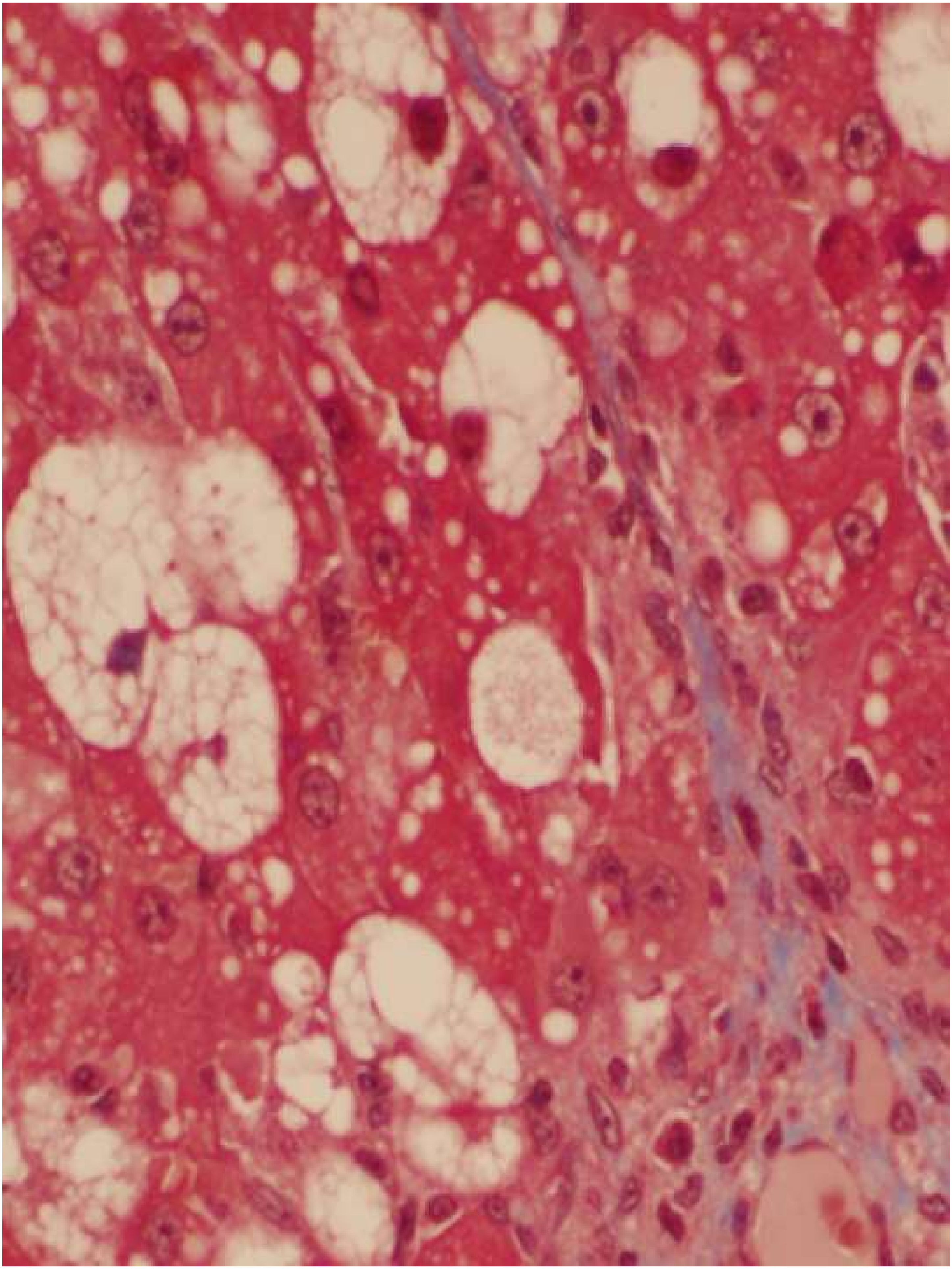

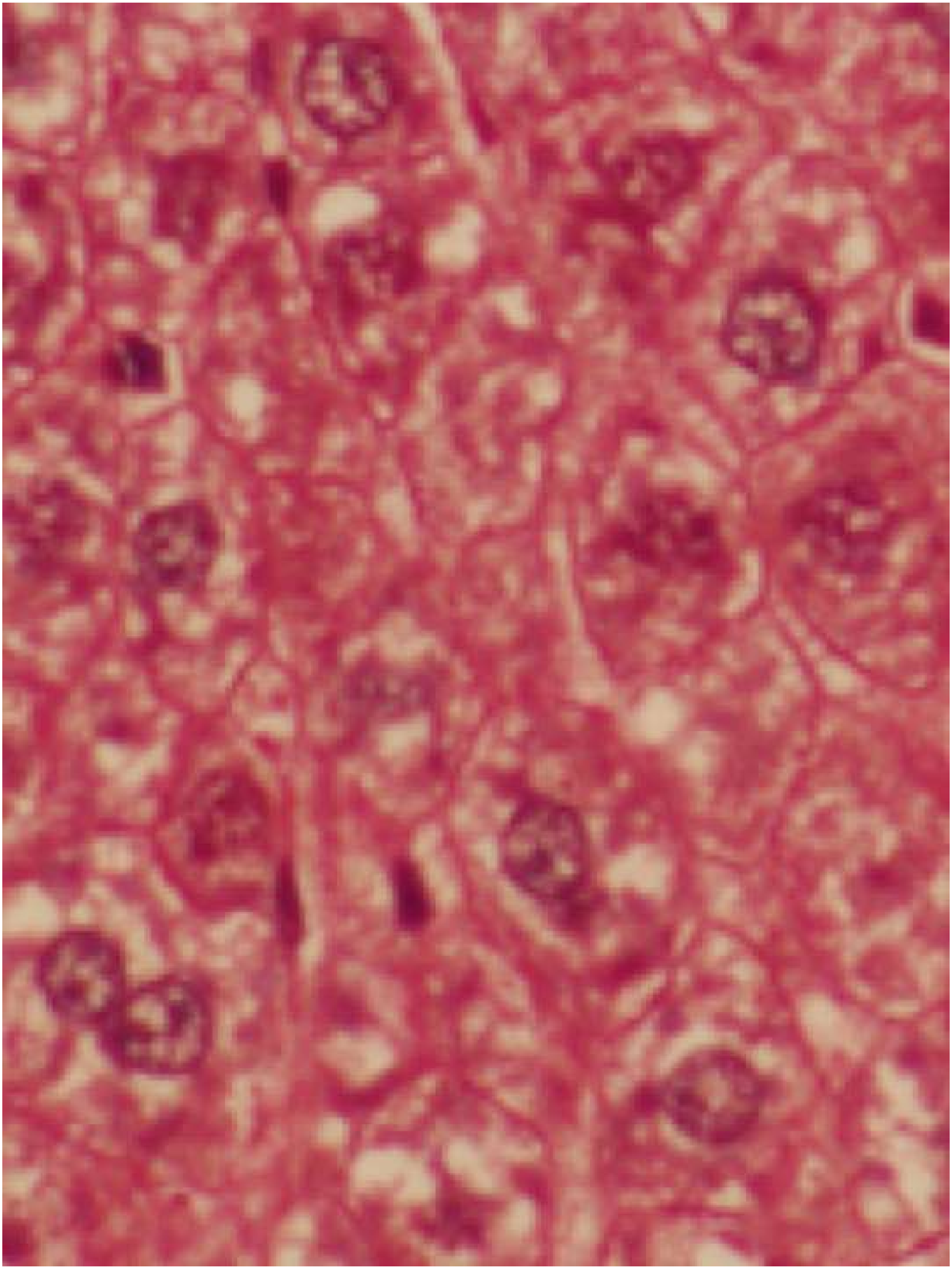

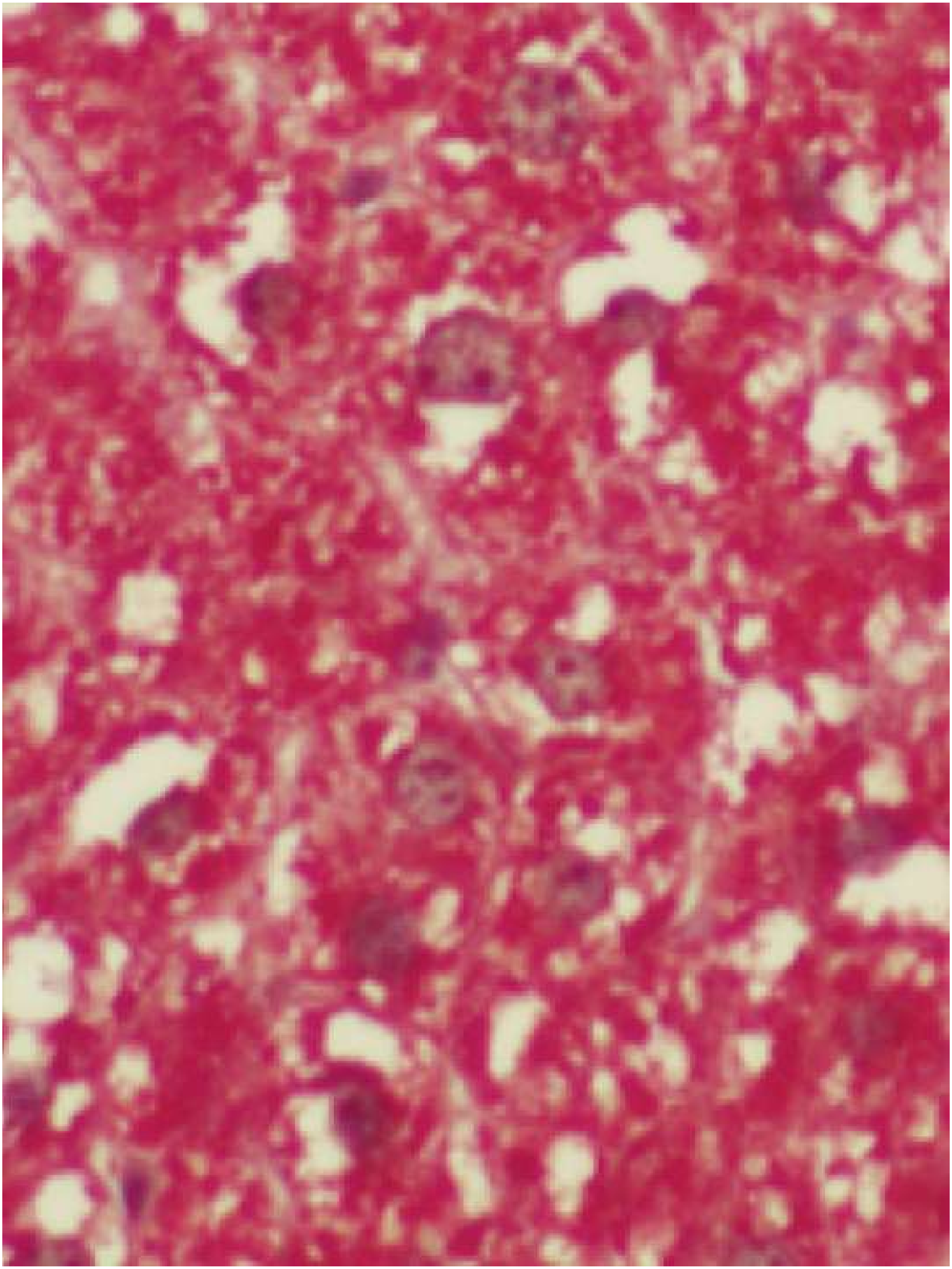

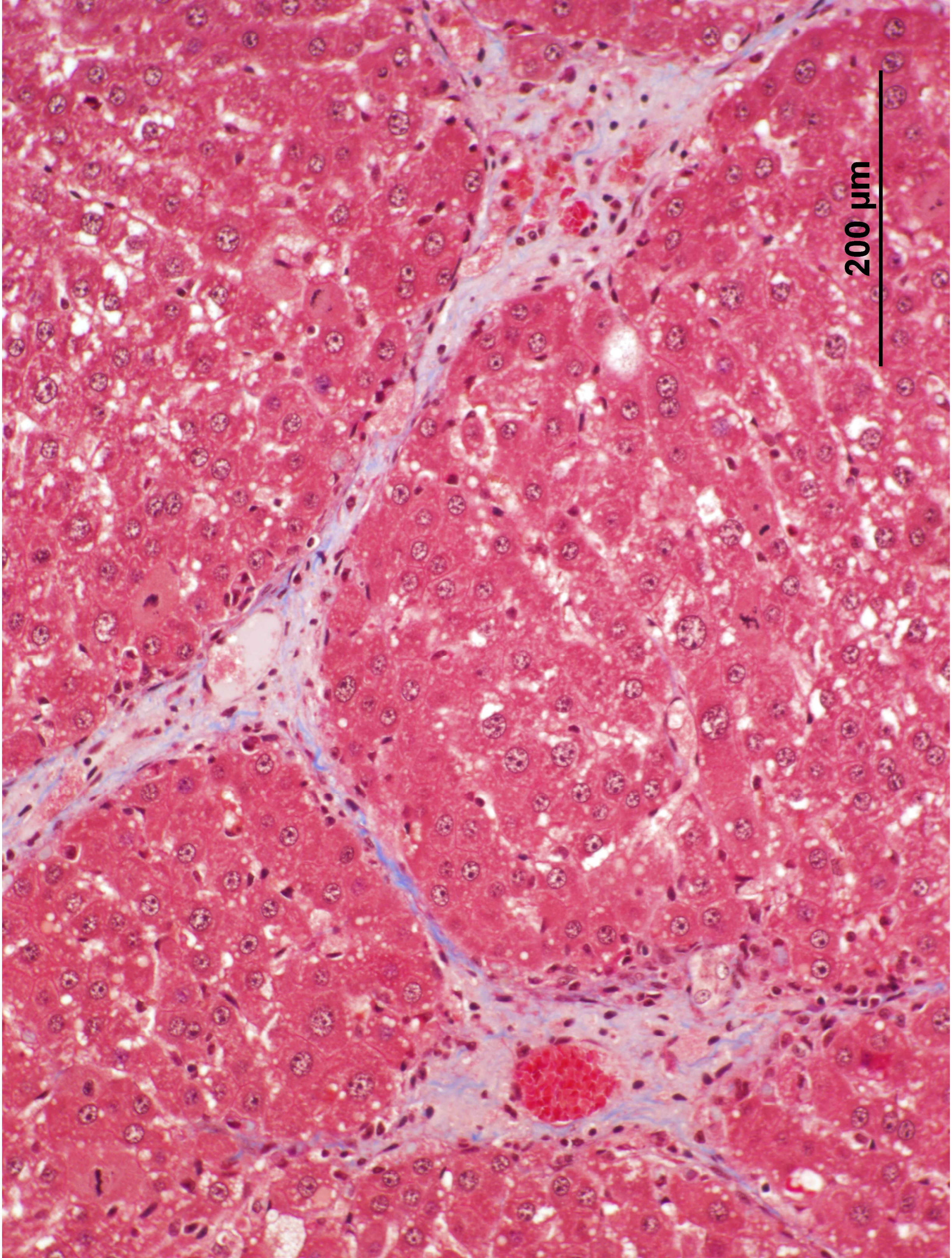
200 µm

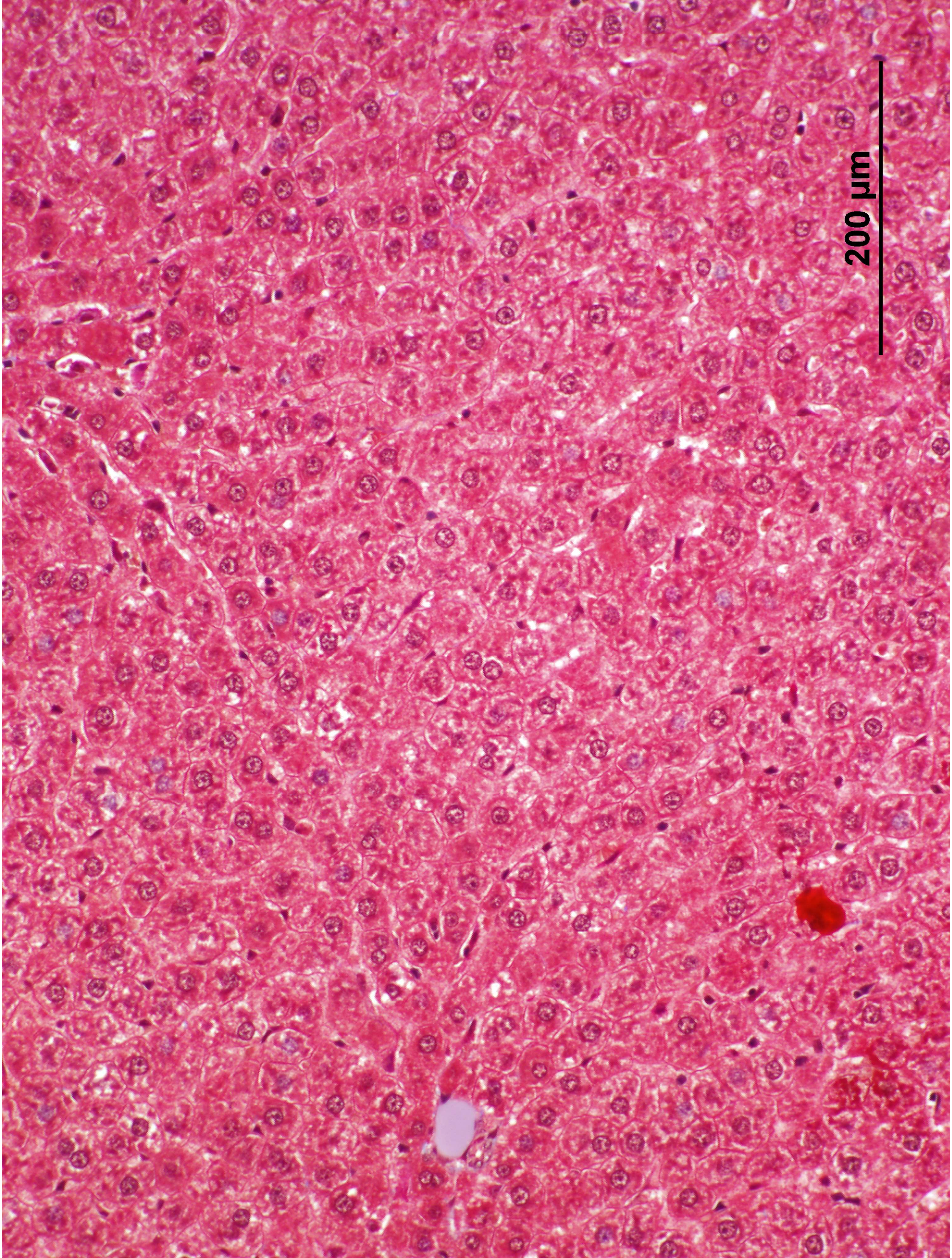
200 µm

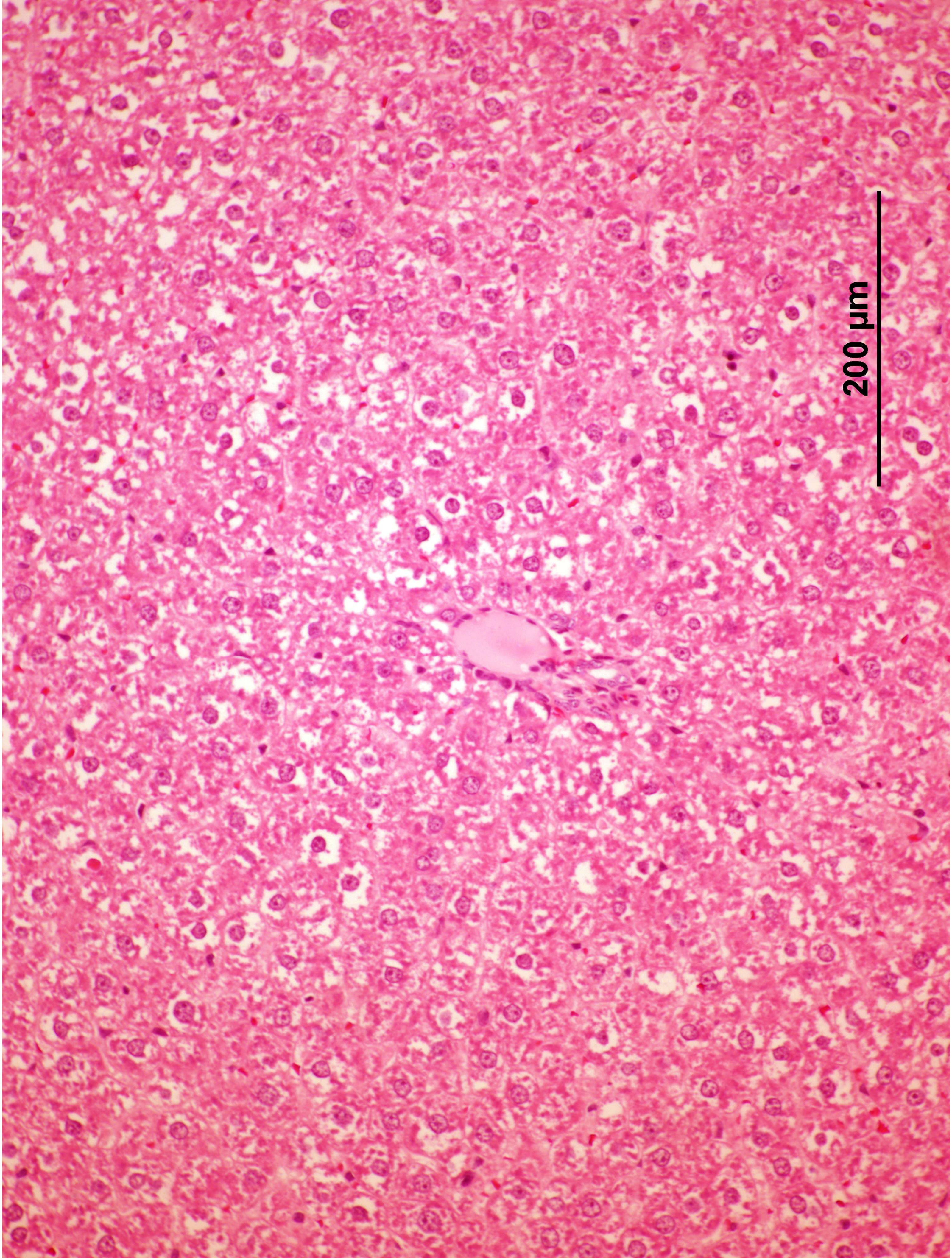
200 µm

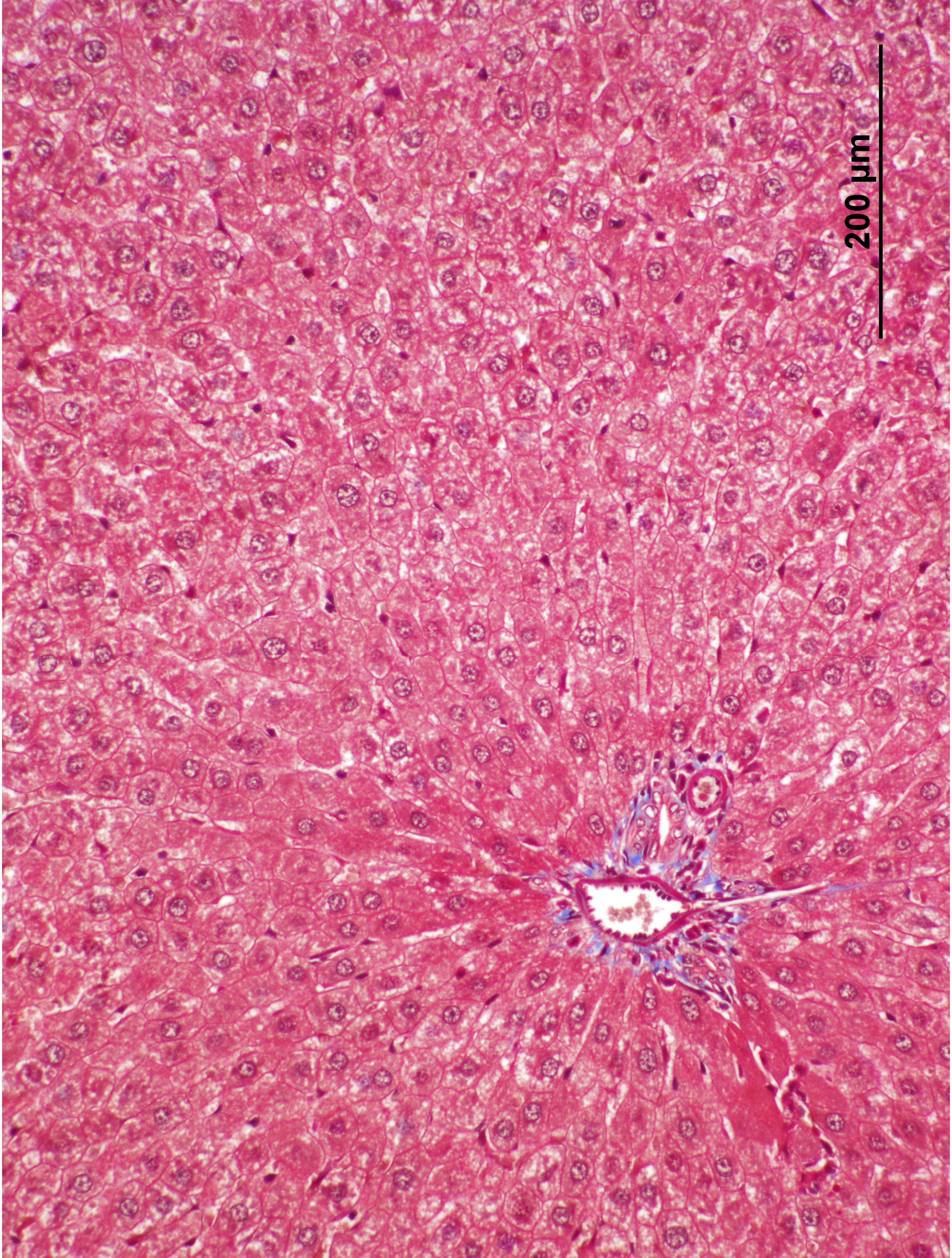
200 µm

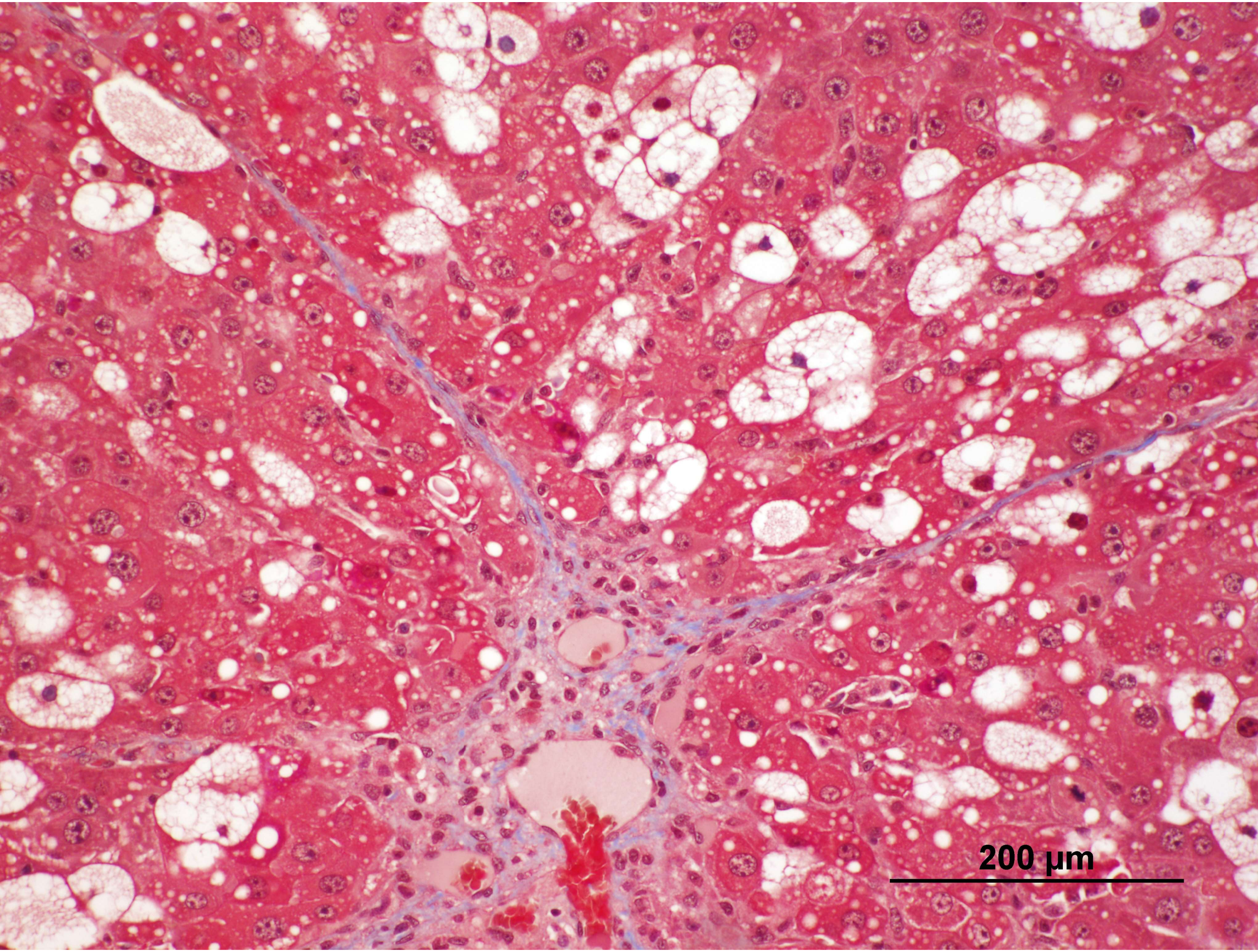
200 µm

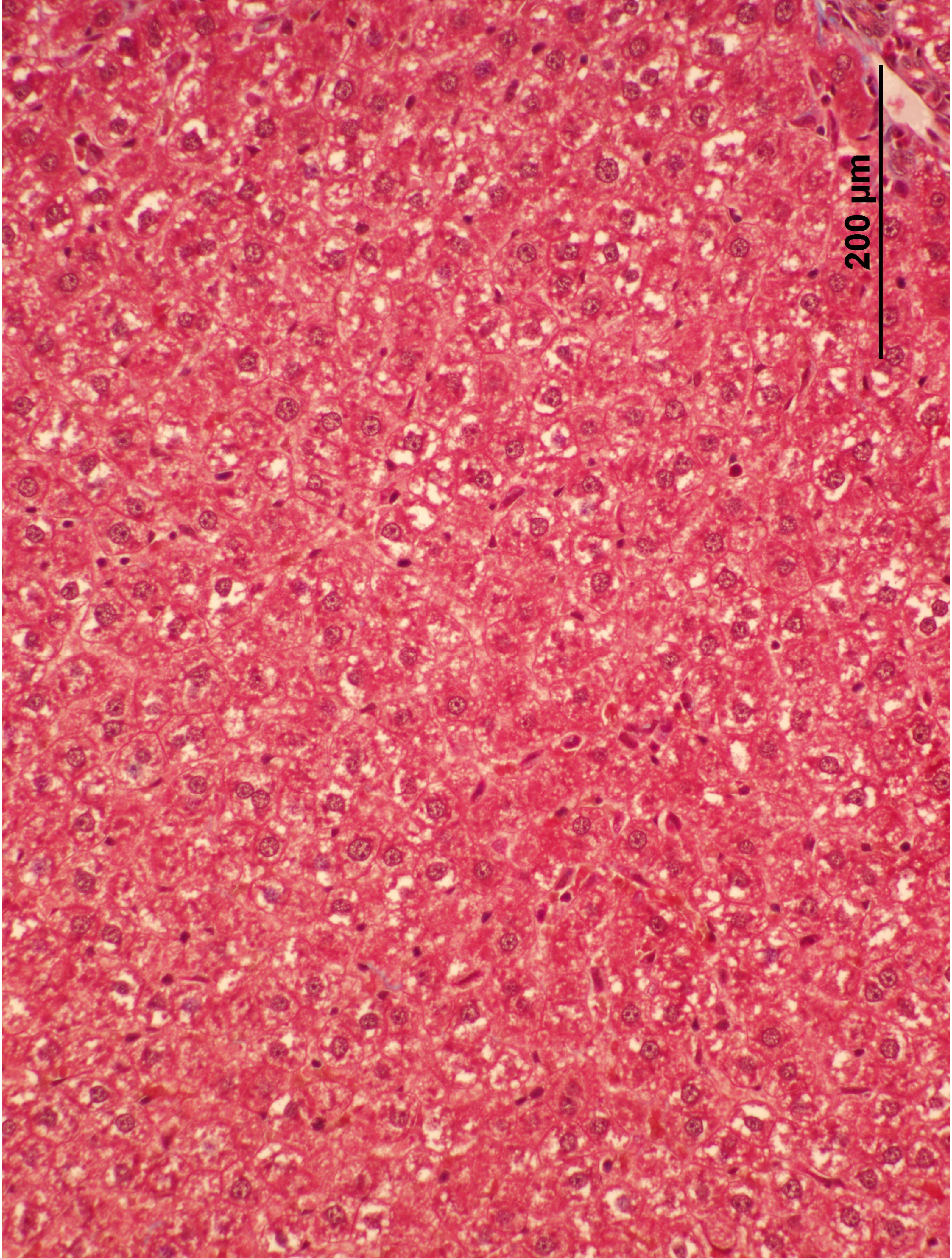
200 μm

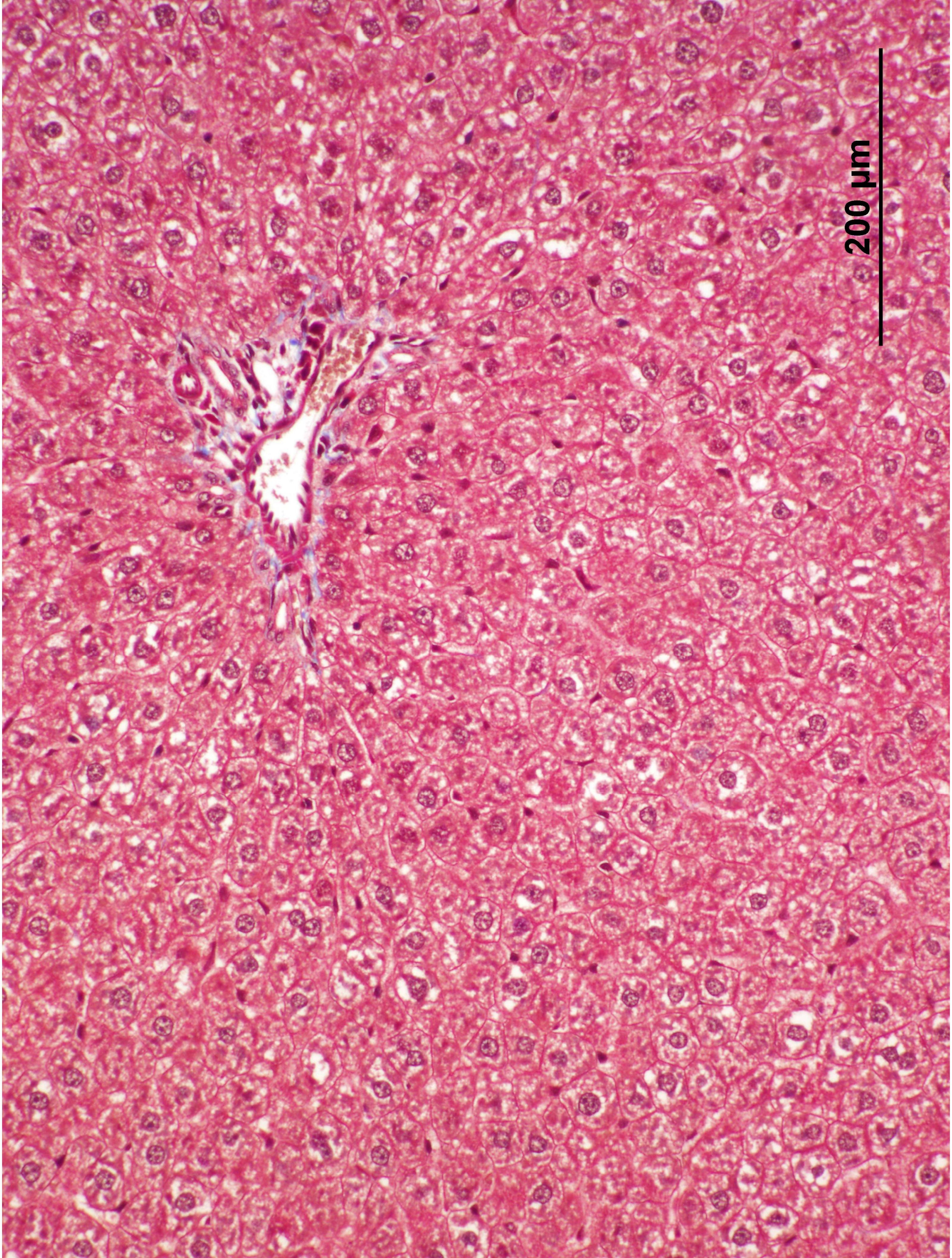
200 µm

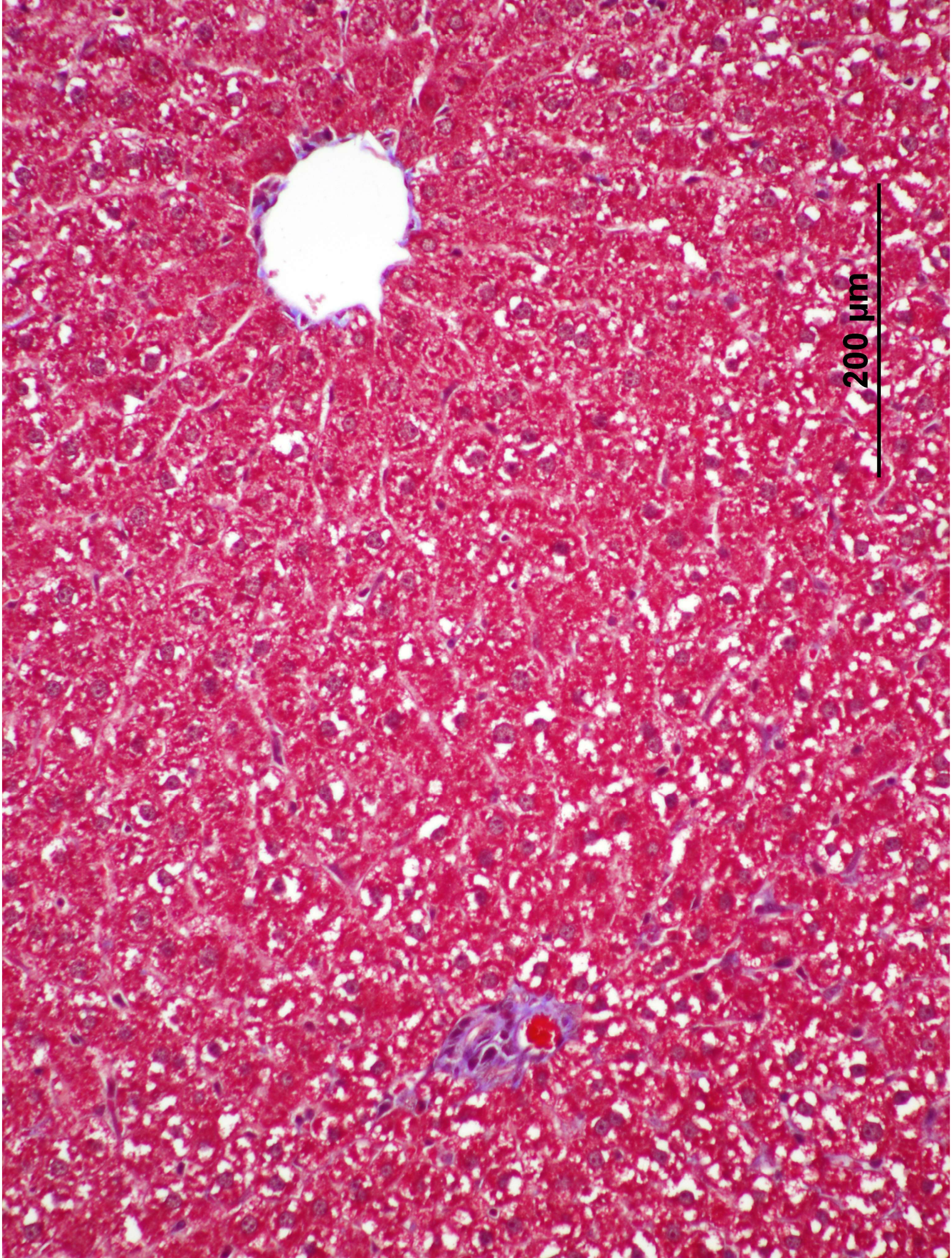
200 µm
