# Supplementary figures and images for: Correction: Tacrolimus (FK506) Prevents Early Stages of Ethanol Induced Hepatic Fibrosis by Targeting LARP6 Dependent Mechanism of Collagen Synthesis
Source: PLoS One. 2024 Jun 20;19(6):e0306020. doi: 10.1371/journal.pone.0306020 (PMC11189226; doi:10.1371/journal.pone.0306020)

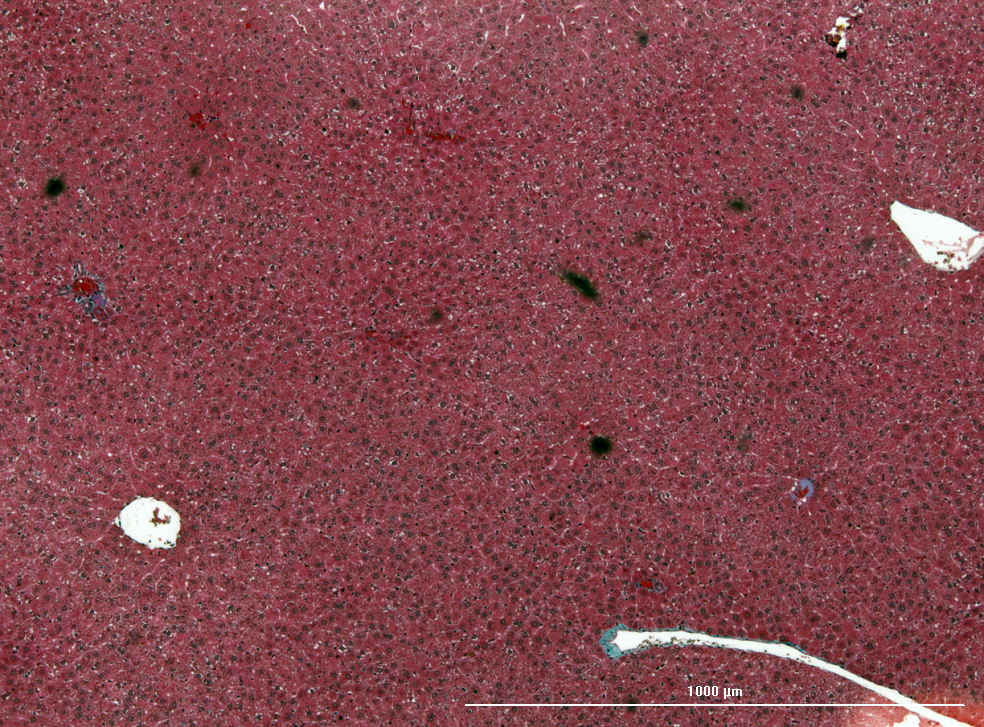

Supplement: S2 File — (ZIP) [file pone.0306020.s002.zip › a ccl+fk506 1.png]

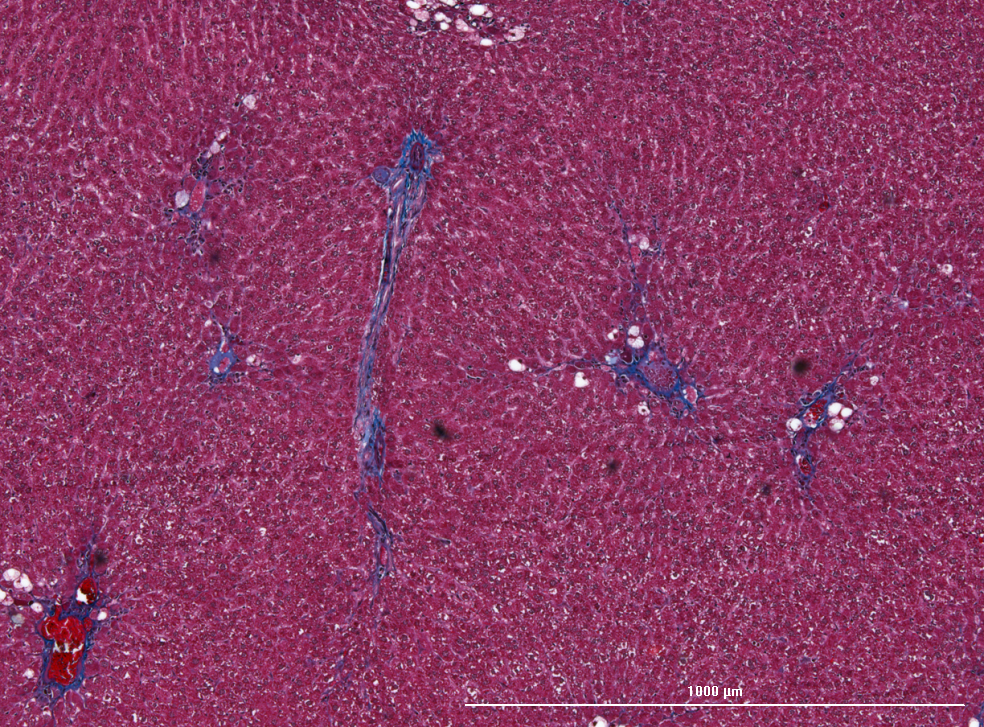

Supplement: S2 File — (ZIP) [file pone.0306020.s002.zip › ccl 1.jpg]

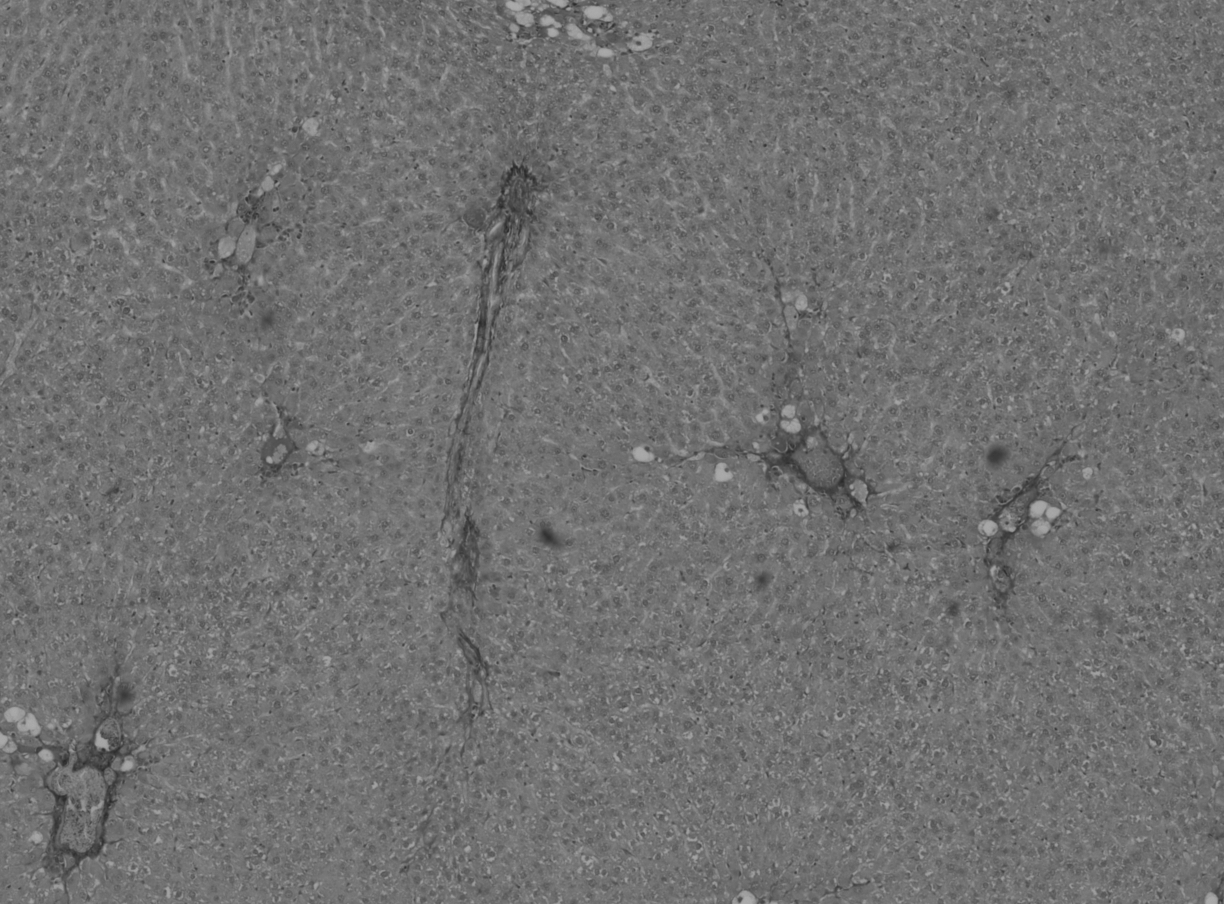

Supplement: S2 File — (ZIP) [file pone.0306020.s002.zip › ccl 1.tif]

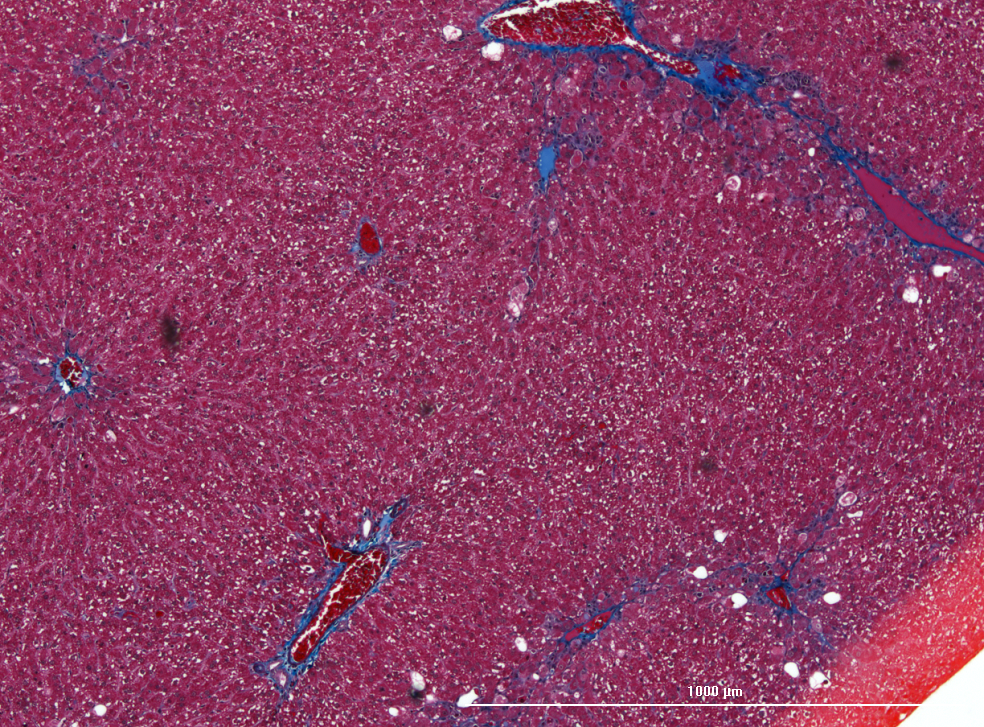

Supplement: S2 File — (ZIP) [file pone.0306020.s002.zip › ccl 2.jpg]

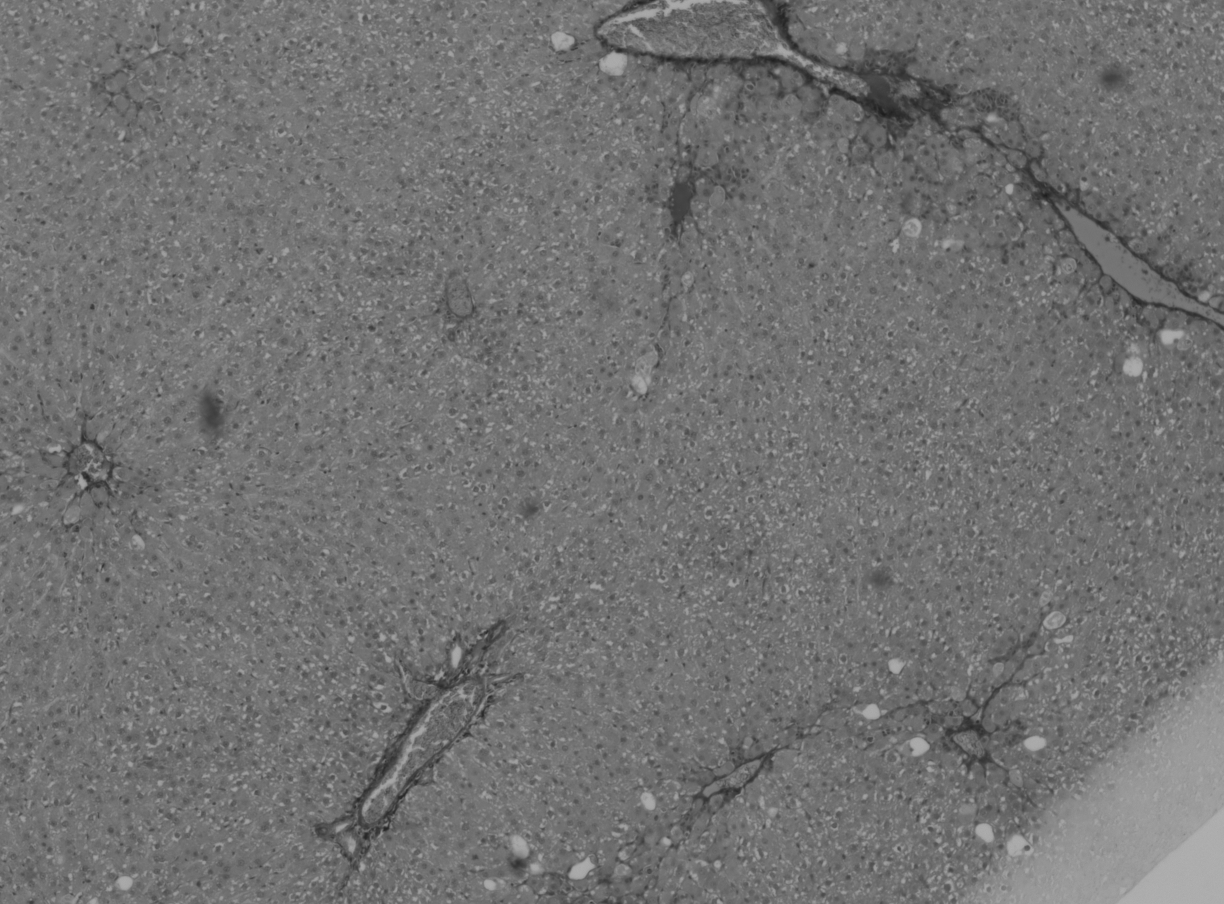

Supplement: S2 File — (ZIP) [file pone.0306020.s002.zip › ccl 2.tif]

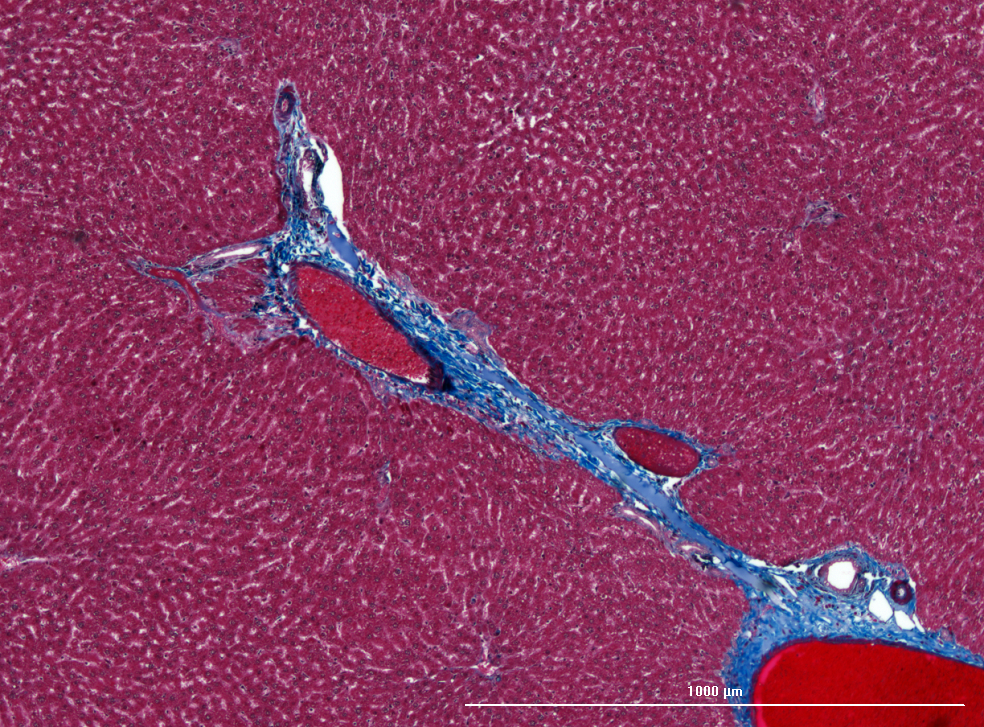

Supplement: S2 File — (ZIP) [file pone.0306020.s002.zip › ccl 3.jpg]

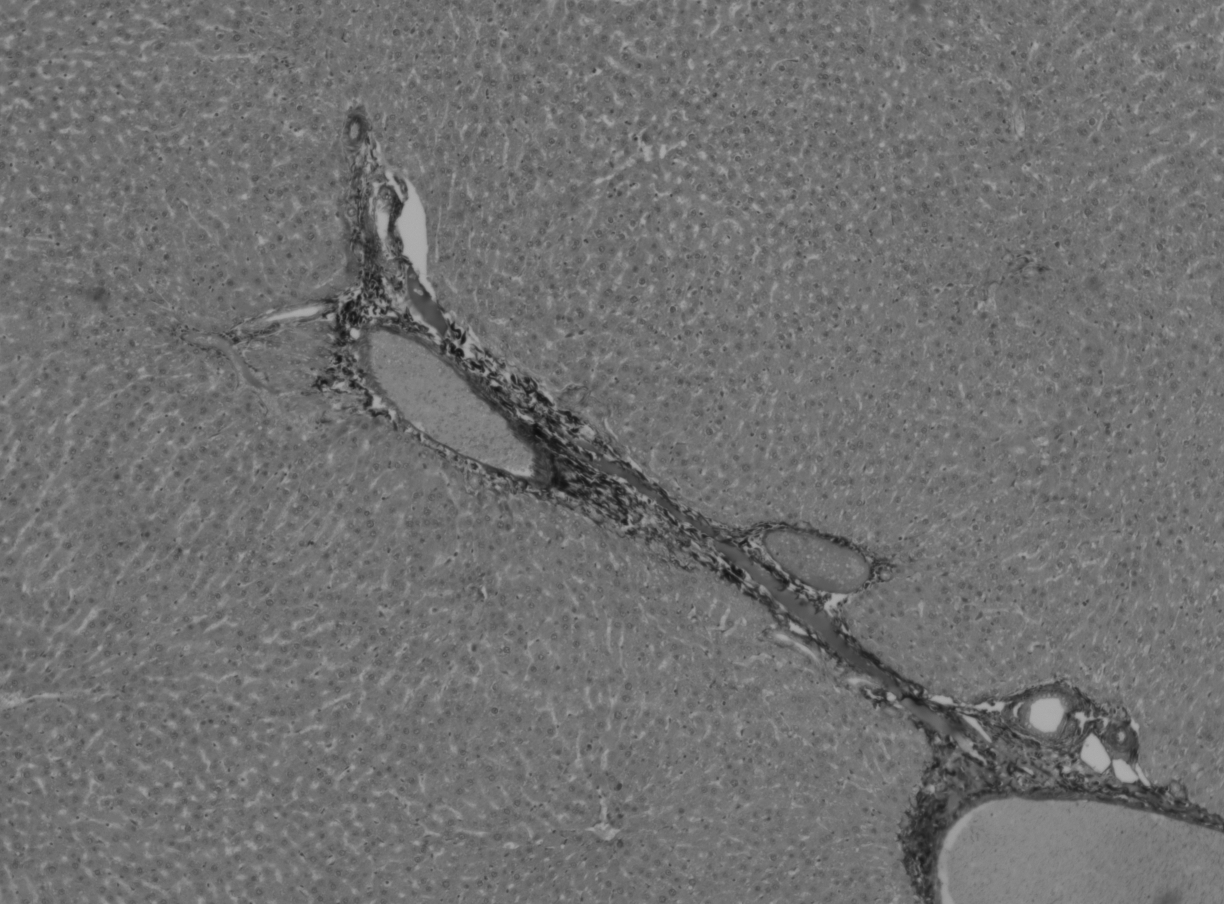

Supplement: S2 File — (ZIP) [file pone.0306020.s002.zip › ccl 3.tif]

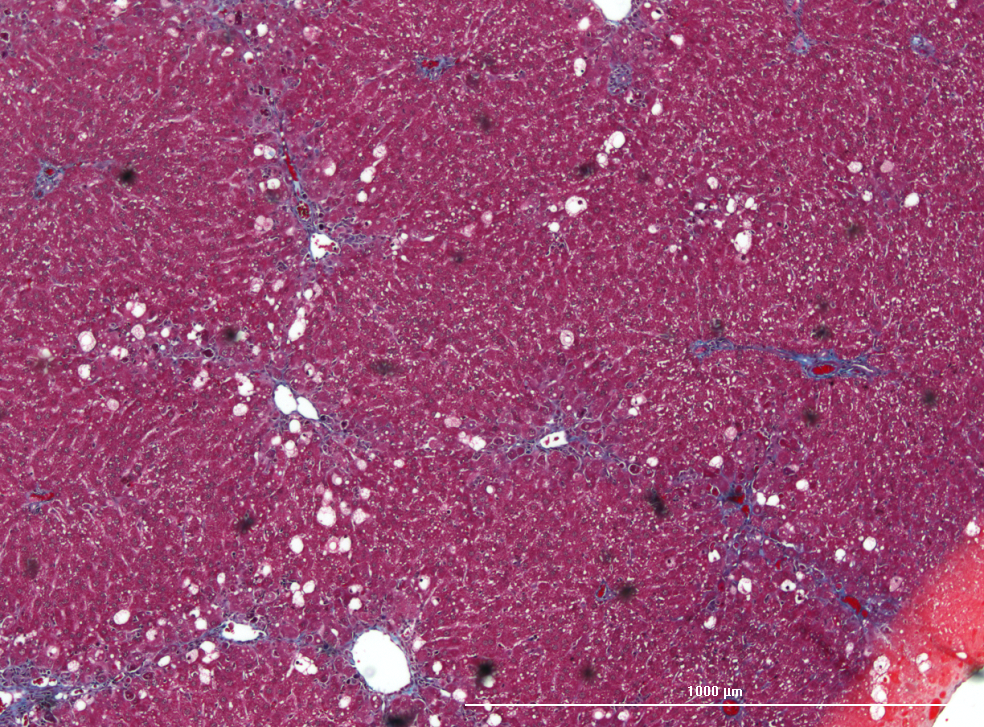

Supplement: S2 File — (ZIP) [file pone.0306020.s002.zip › ccl 4.jpg]

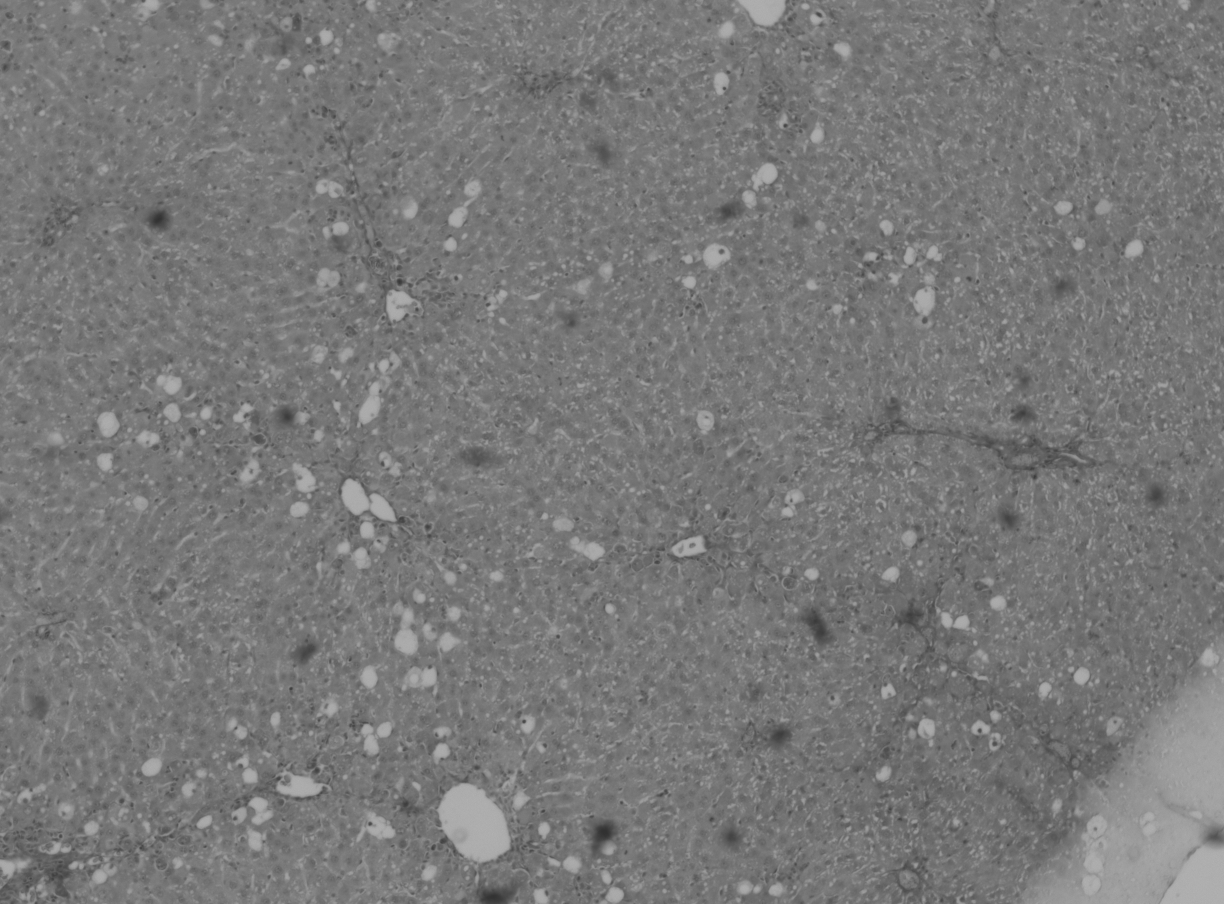

Supplement: S2 File — (ZIP) [file pone.0306020.s002.zip › ccl 4.tif]

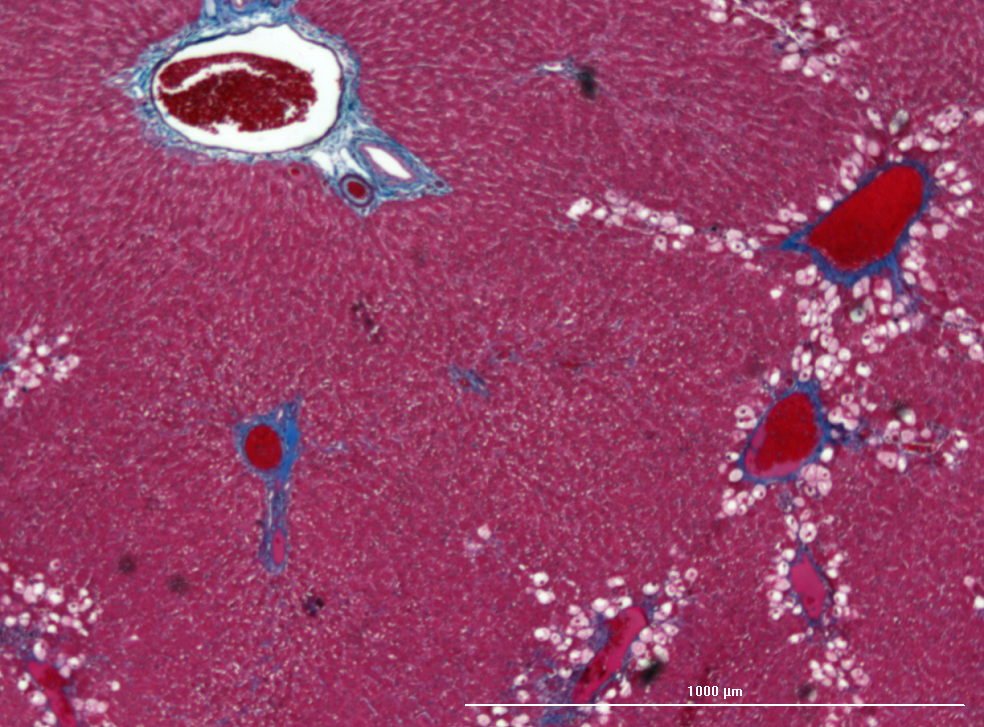

Supplement: S2 File — (ZIP) [file pone.0306020.s002.zip › ccl 5.jpg]

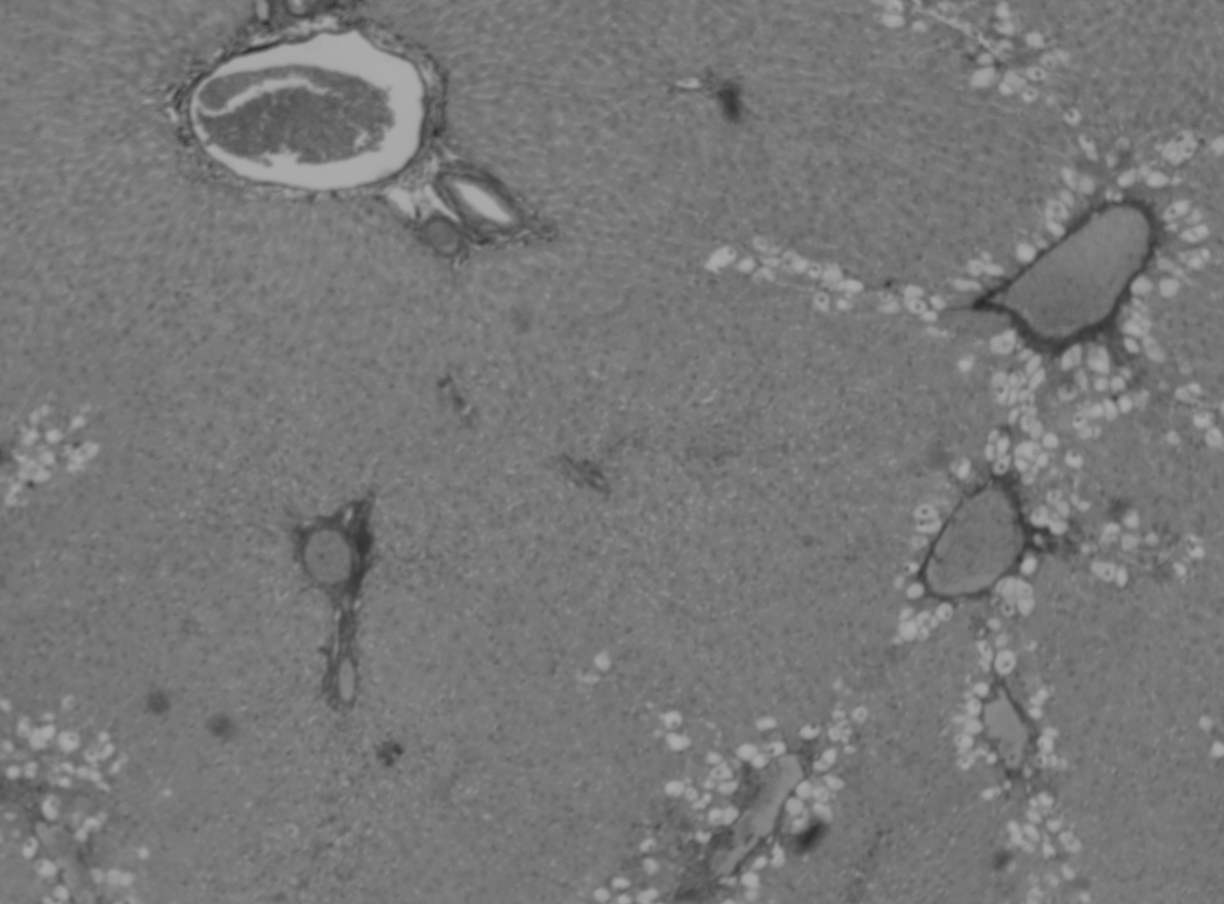

Supplement: S2 File — (ZIP) [file pone.0306020.s002.zip › ccl 5.tif]

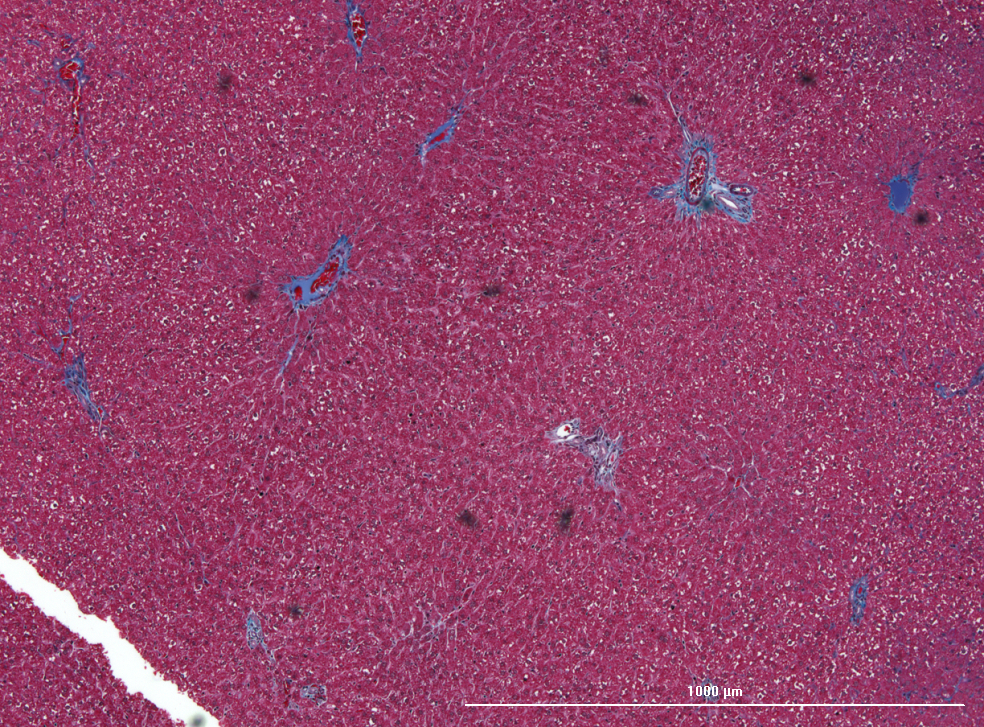

Supplement: S2 File — (ZIP) [file pone.0306020.s002.zip › ccl 6.jpg]

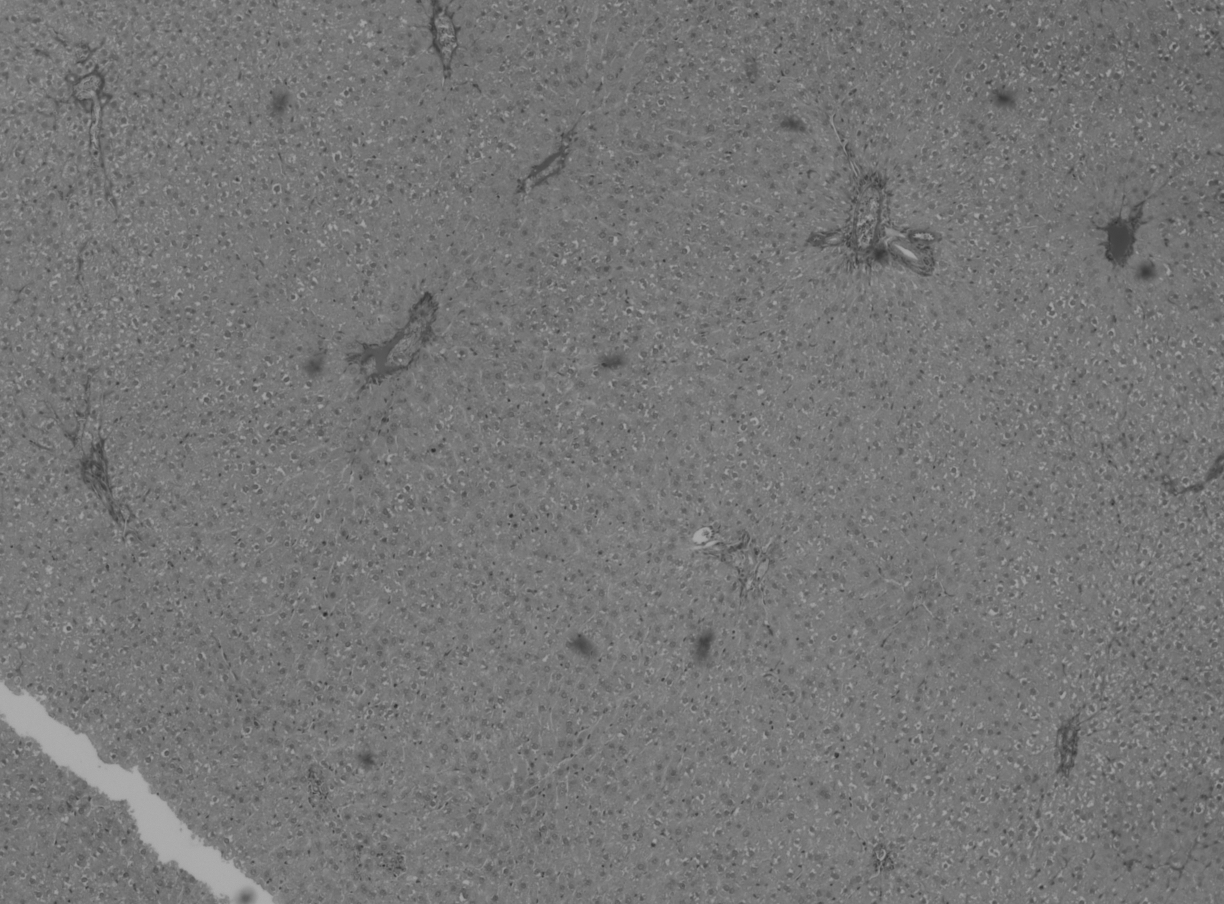

Supplement: S2 File — (ZIP) [file pone.0306020.s002.zip › ccl 6.tif]

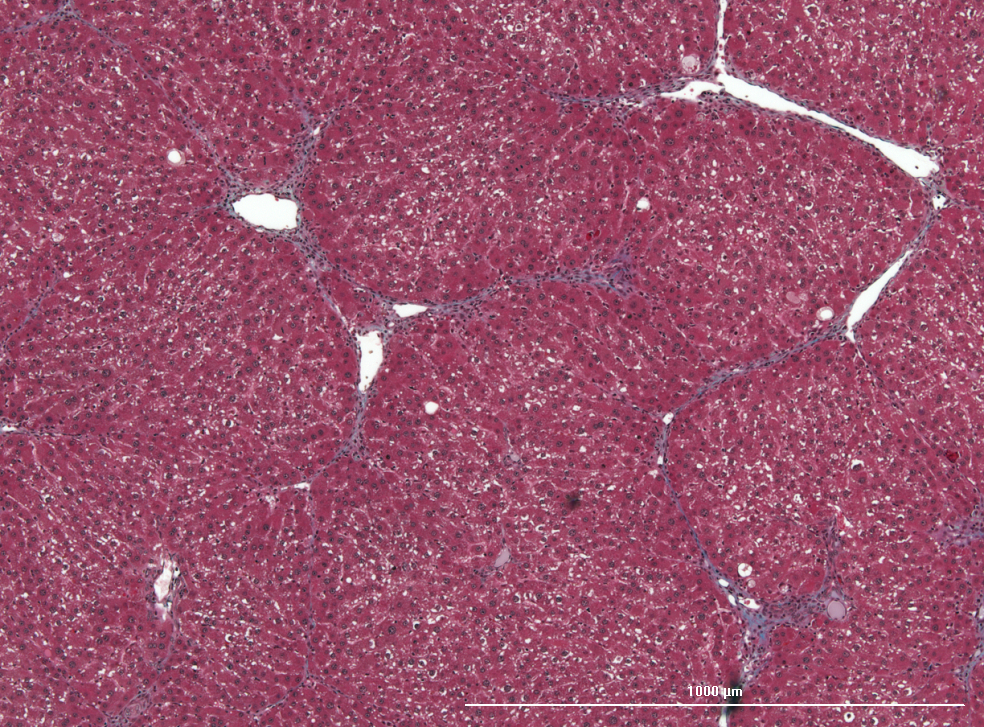

Supplement: S2 File — (ZIP) [file pone.0306020.s002.zip › ccl 7.jpg]

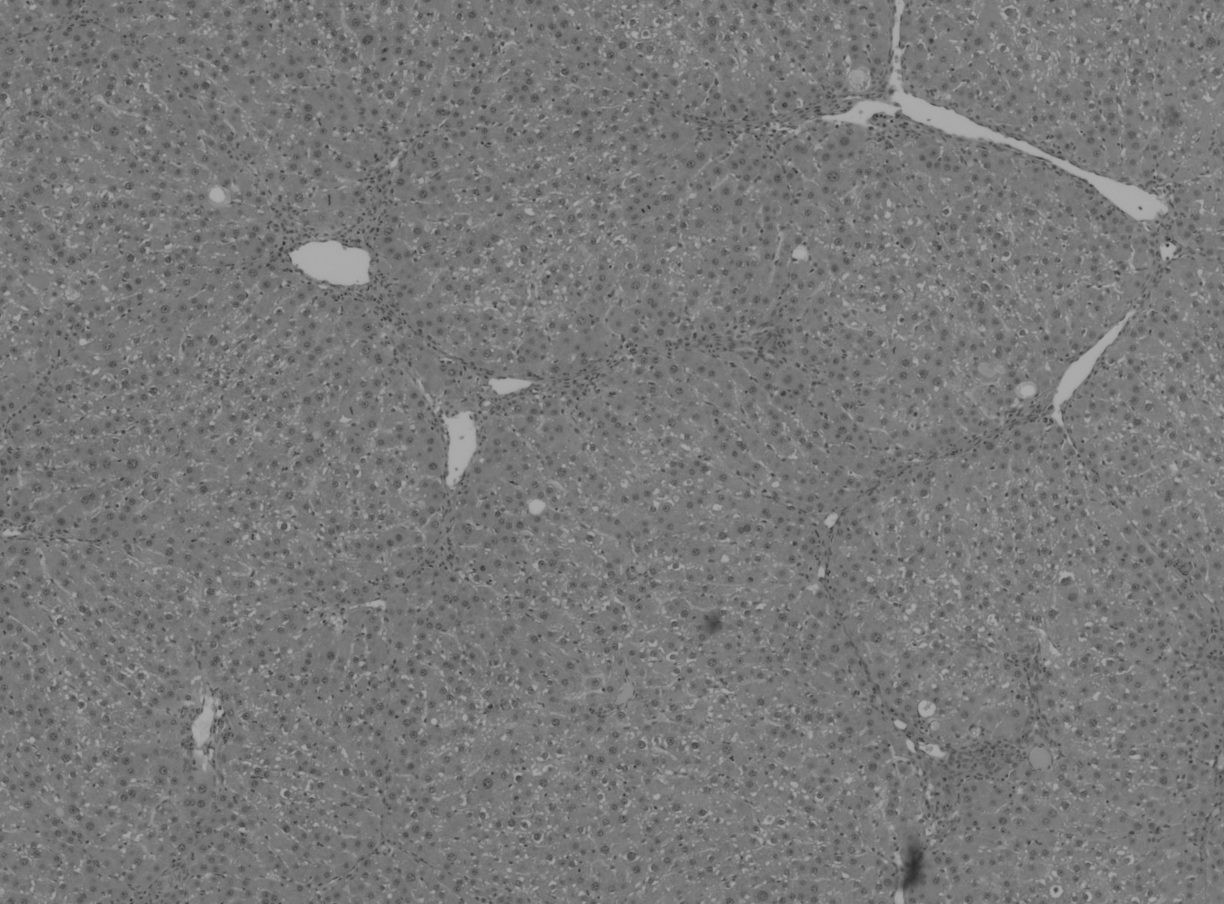

Supplement: S2 File — (ZIP) [file pone.0306020.s002.zip › ccl 7.tif]

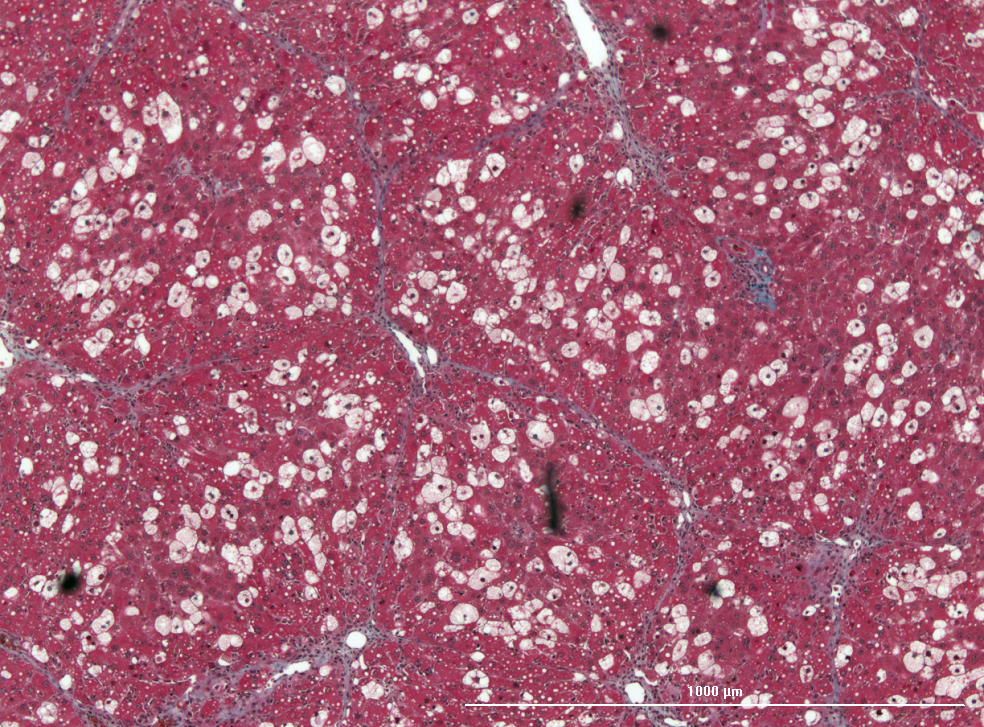

Supplement: S2 File — (ZIP) [file pone.0306020.s002.zip › ccl 8.jpg]

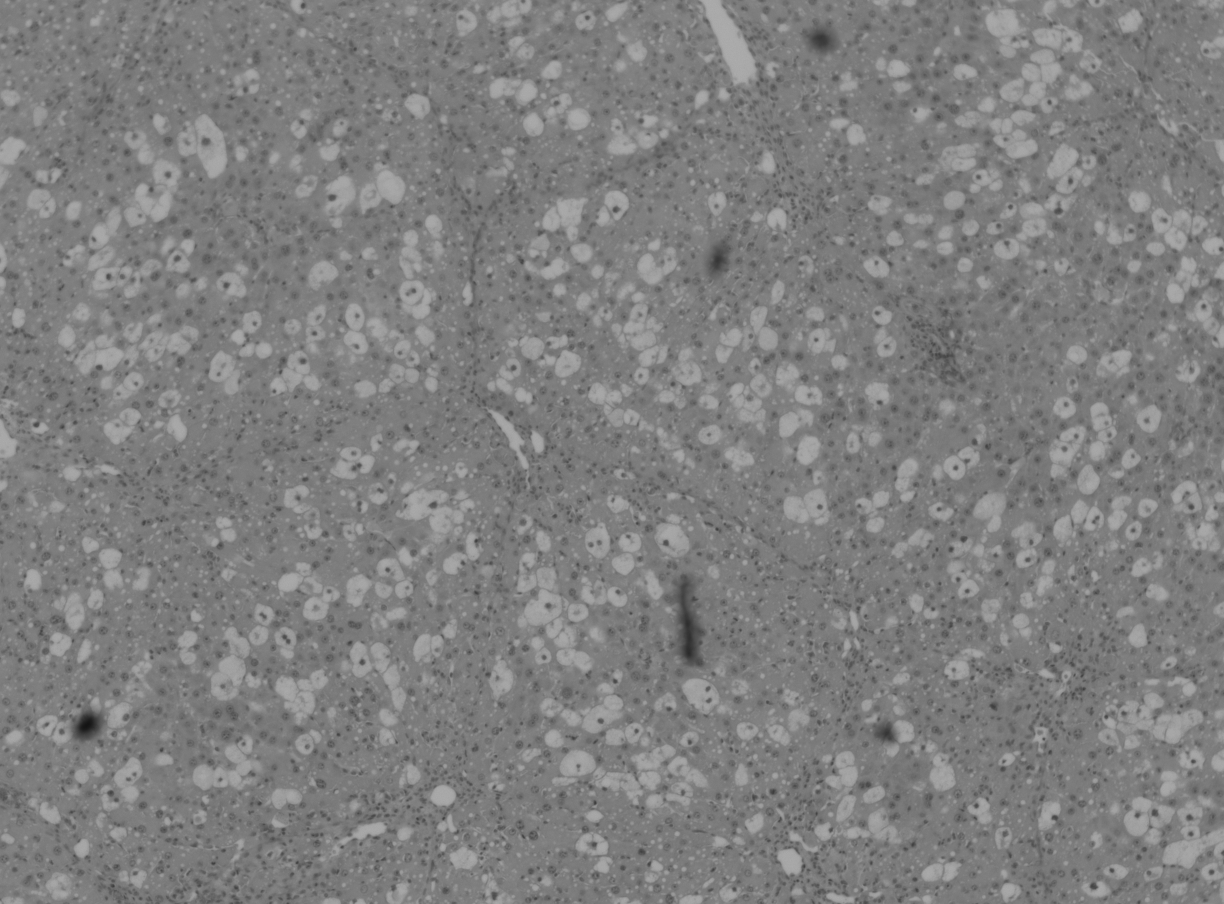

Supplement: S2 File — (ZIP) [file pone.0306020.s002.zip › ccl 8.tif]

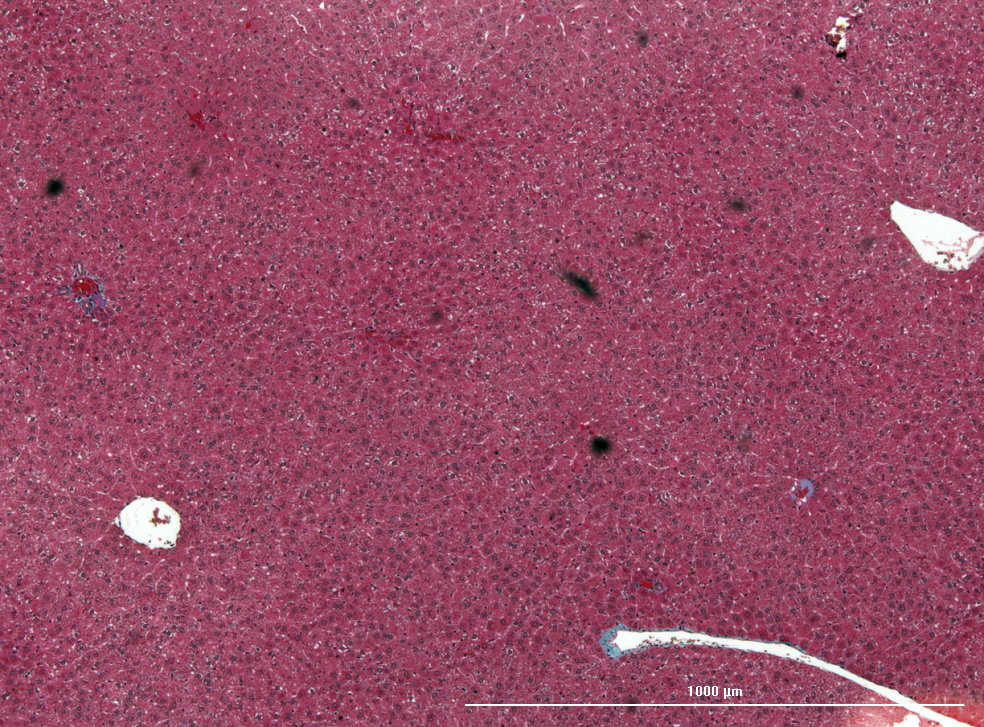

Supplement: S2 File — (ZIP) [file pone.0306020.s002.zip › ccl+fk506 1.jpg]

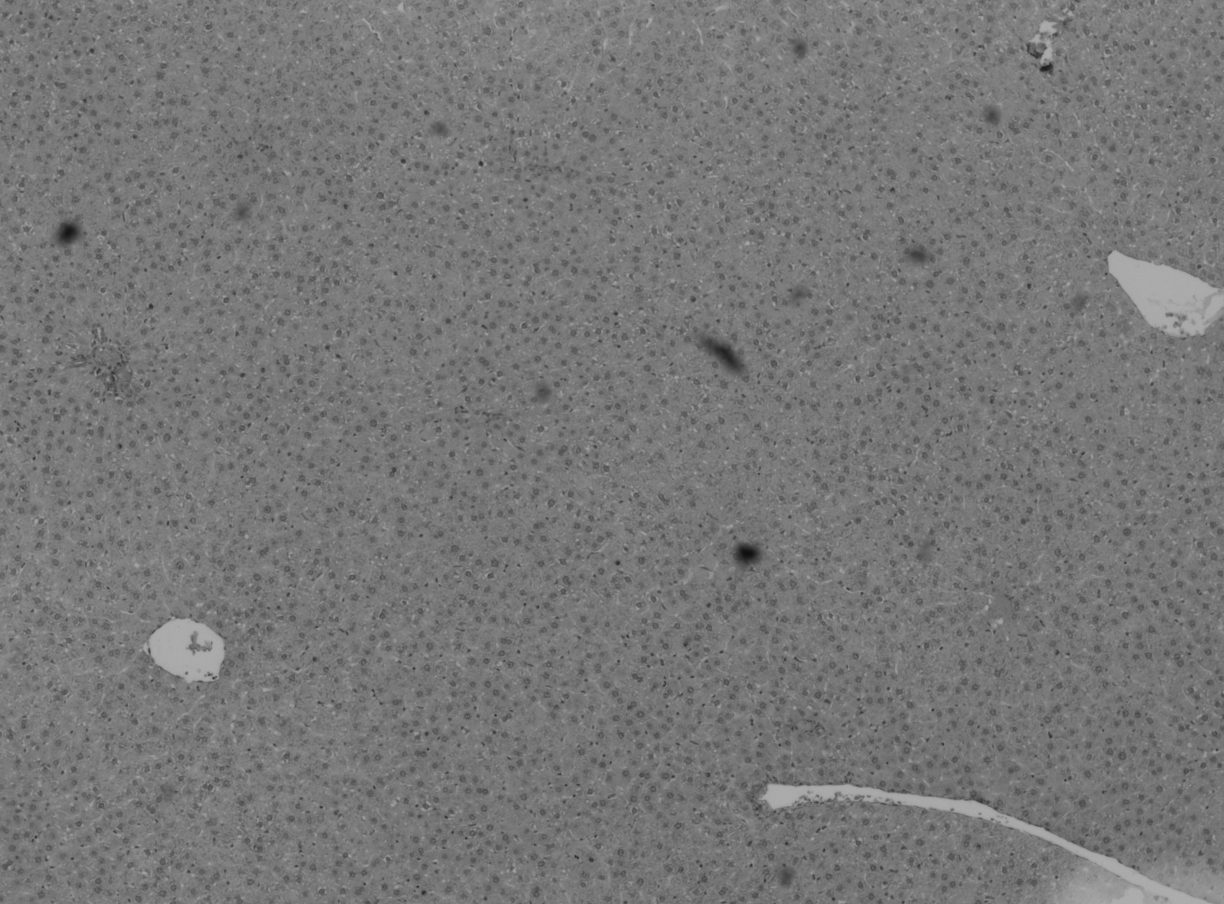

Supplement: S2 File — (ZIP) [file pone.0306020.s002.zip › ccl+fk506 1.tif]

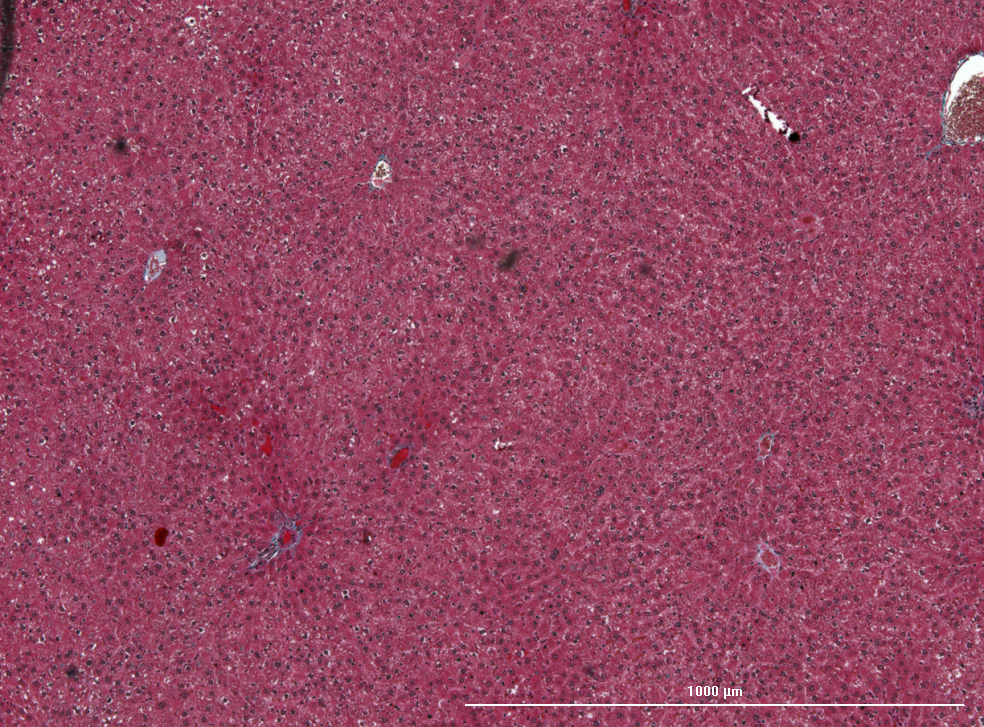

Supplement: S3 File — (ZIP) [file pone.0306020.s003.zip › ccl+fk506 2.jpg]

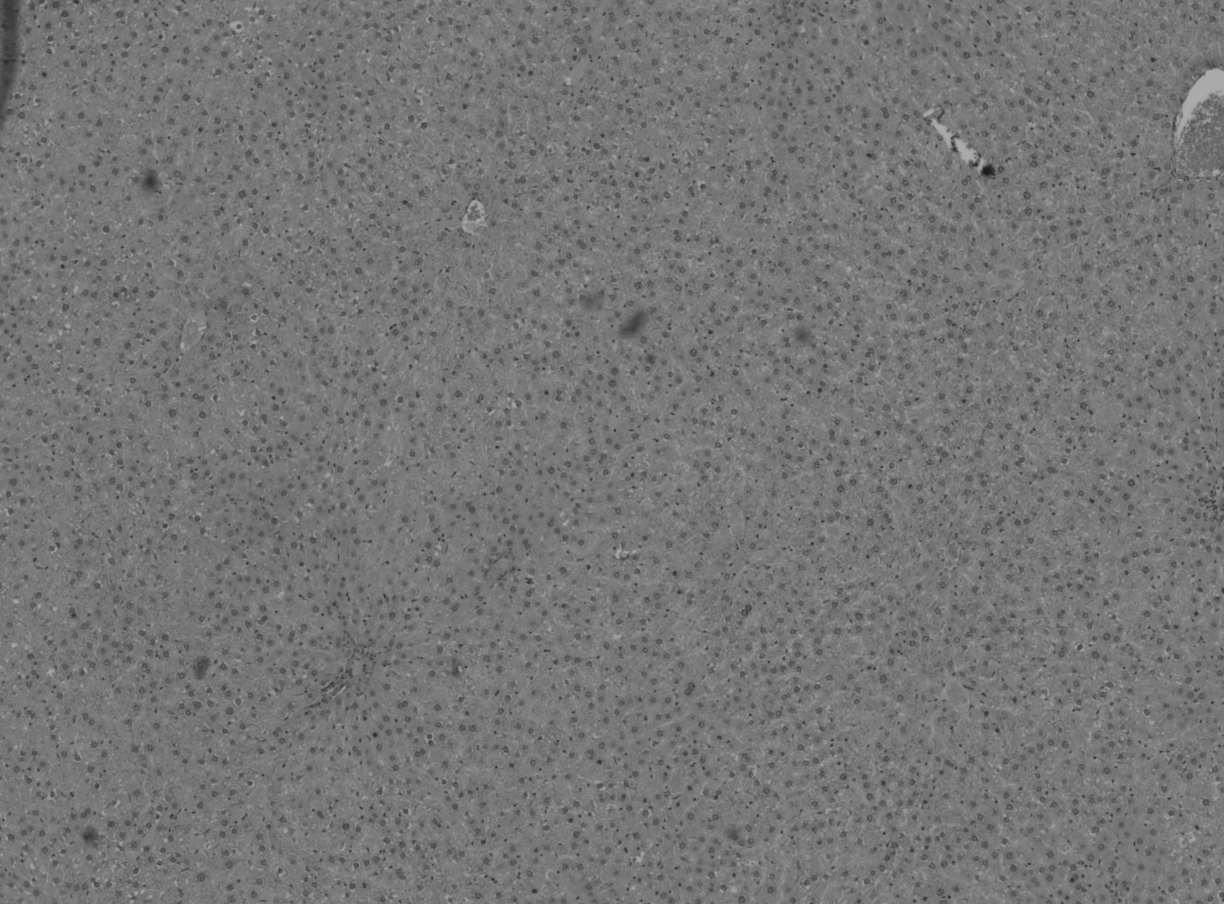

Supplement: S3 File — (ZIP) [file pone.0306020.s003.zip › ccl+fk506 2.tif]

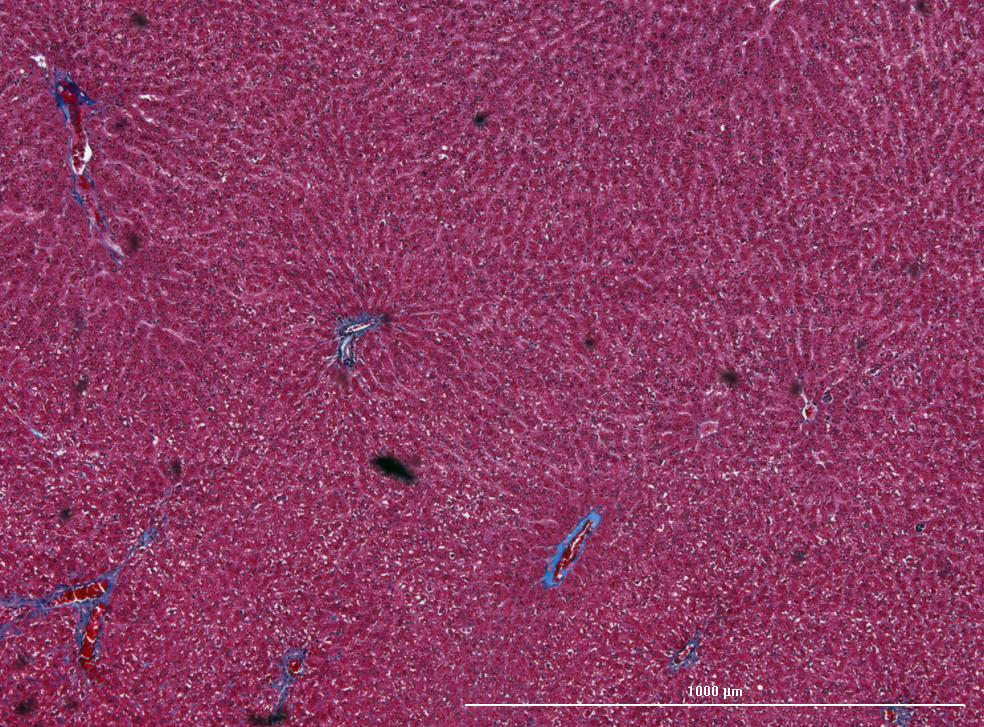

Supplement: S3 File — (ZIP) [file pone.0306020.s003.zip › ccl+fk506 3.jpg]

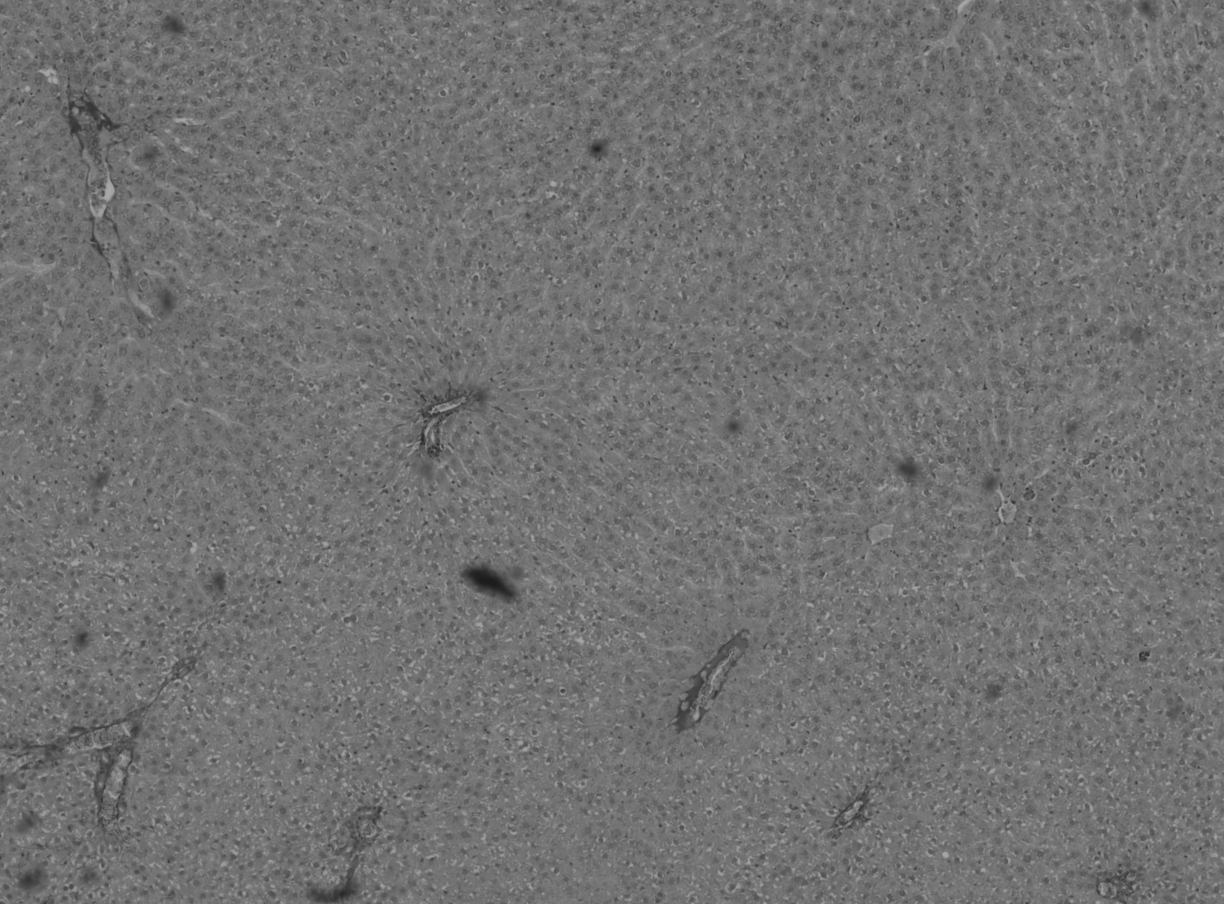

Supplement: S3 File — (ZIP) [file pone.0306020.s003.zip › ccl+fk506 3.tif]

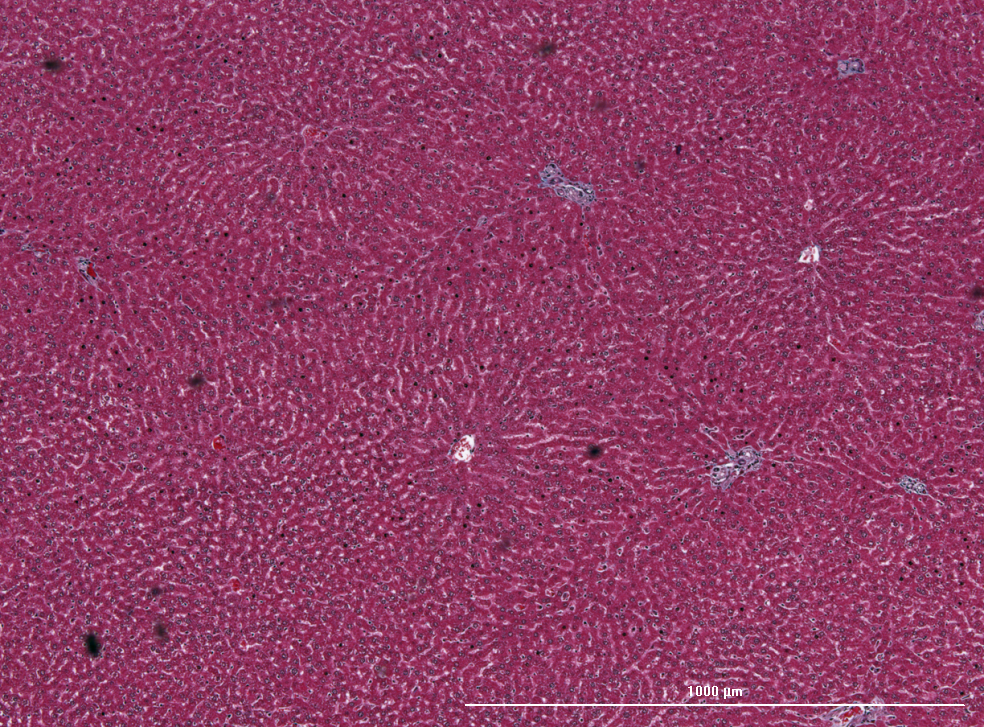

Supplement: S3 File — (ZIP) [file pone.0306020.s003.zip › ccl+fk506 4.jpg]

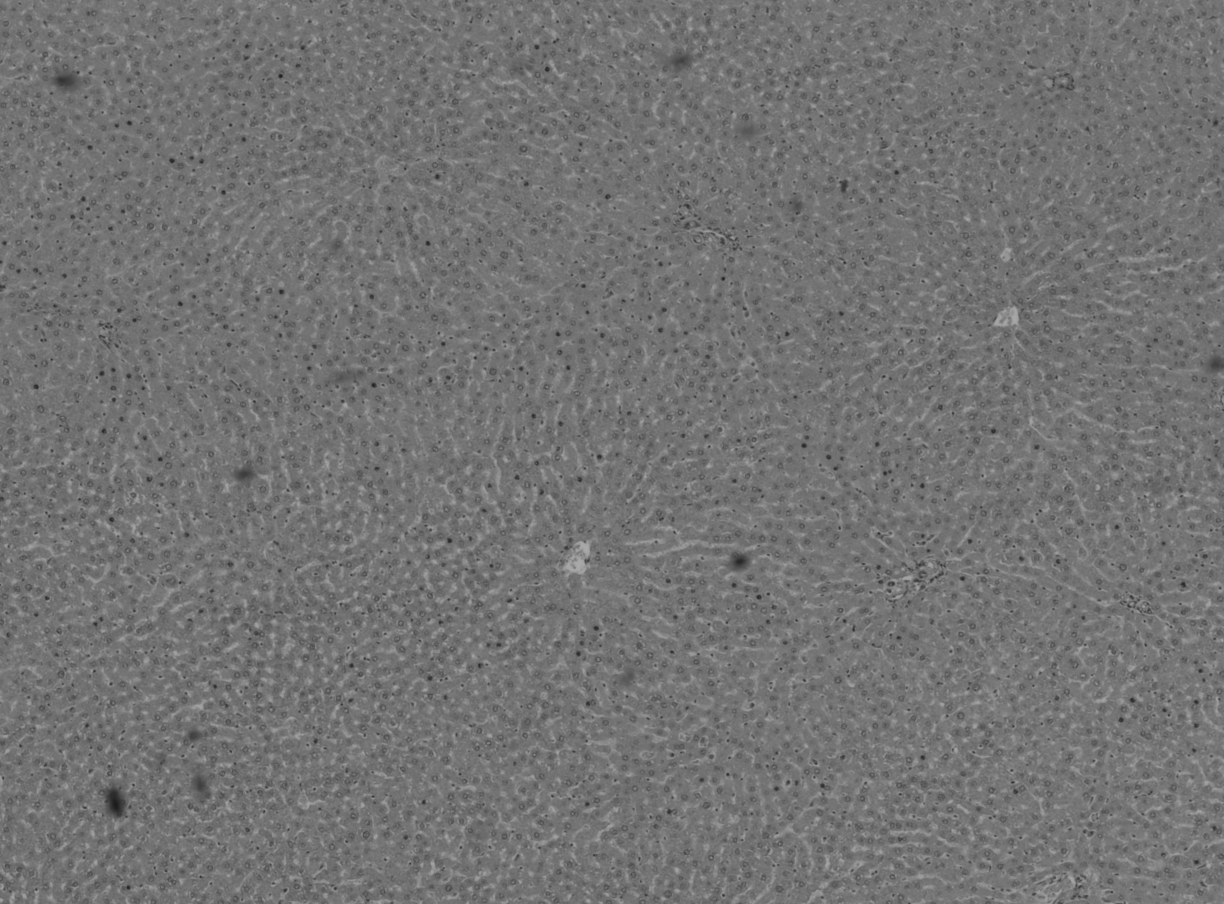

Supplement: S3 File — (ZIP) [file pone.0306020.s003.zip › ccl+fk506 4.tif]

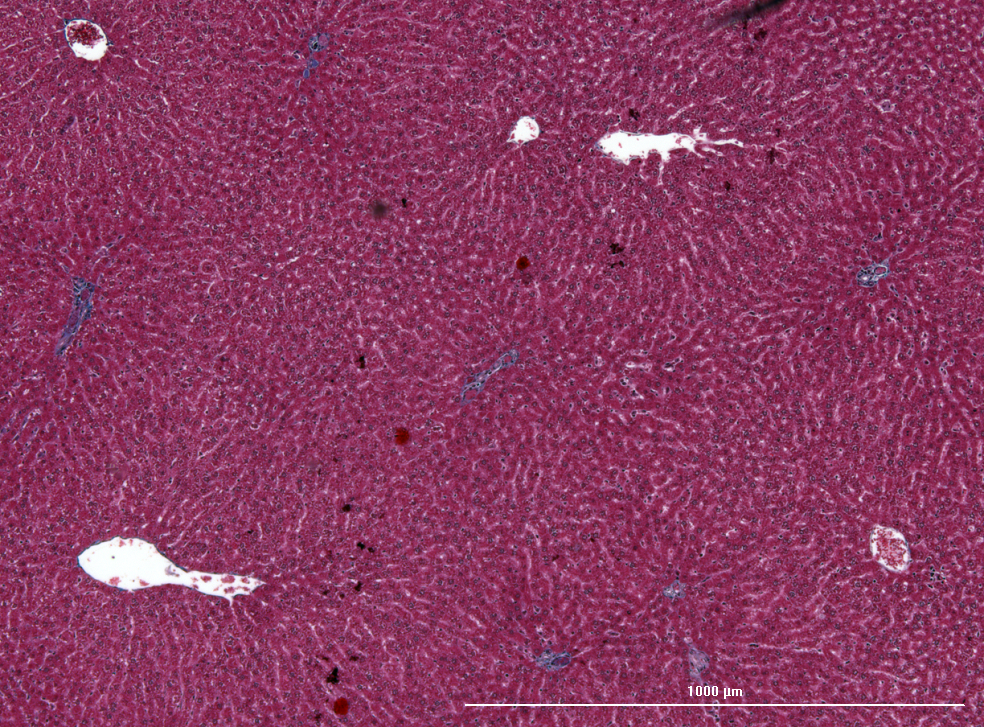

Supplement: S3 File — (ZIP) [file pone.0306020.s003.zip › ccl+fk506 5.jpg]

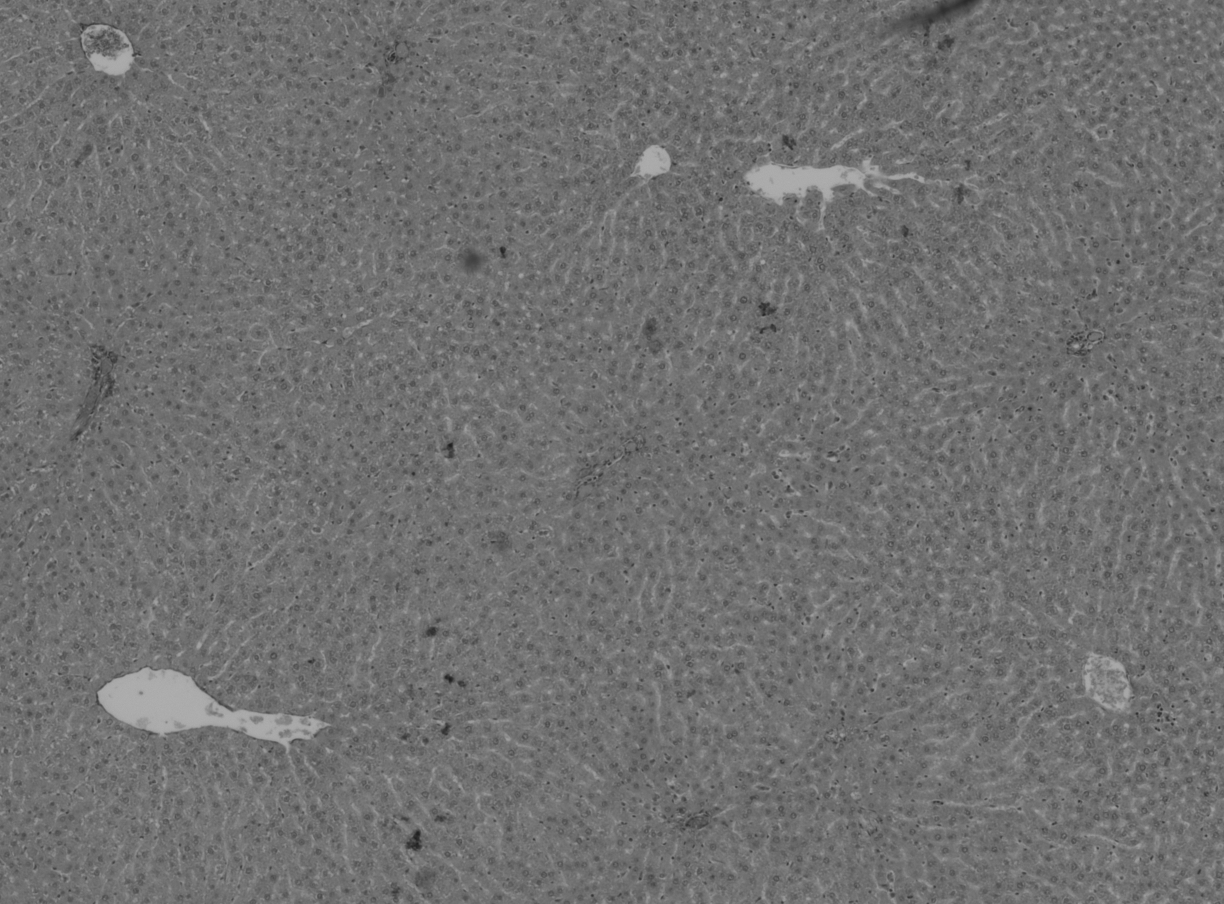

Supplement: S3 File — (ZIP) [file pone.0306020.s003.zip › ccl+fk506 5.tif]

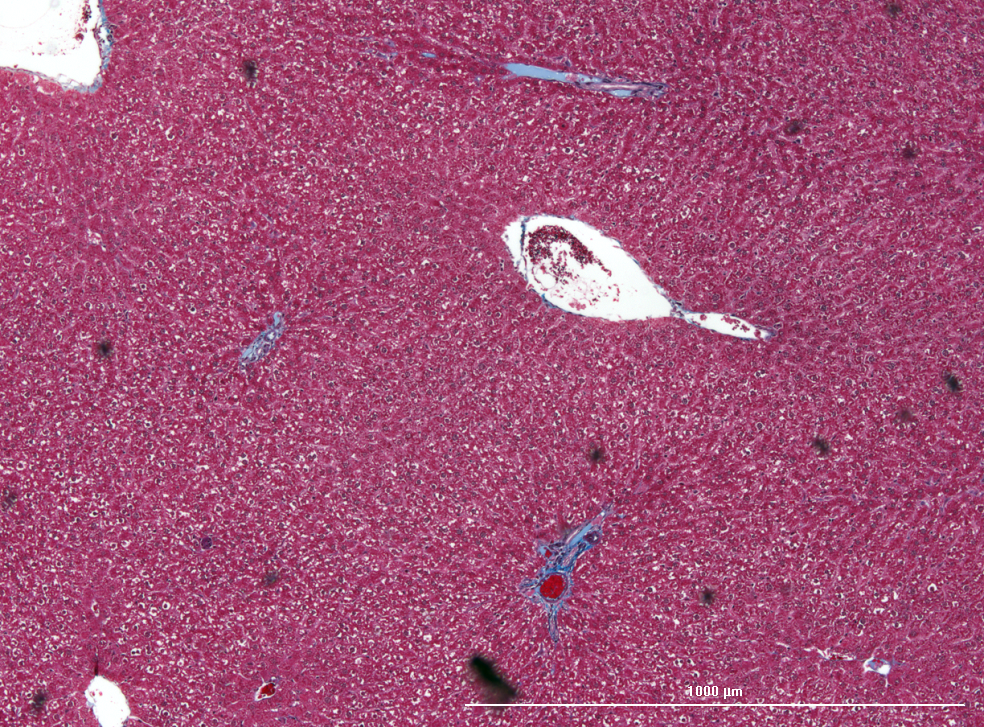

Supplement: S3 File — (ZIP) [file pone.0306020.s003.zip › ccl+fk506 6.jpg]

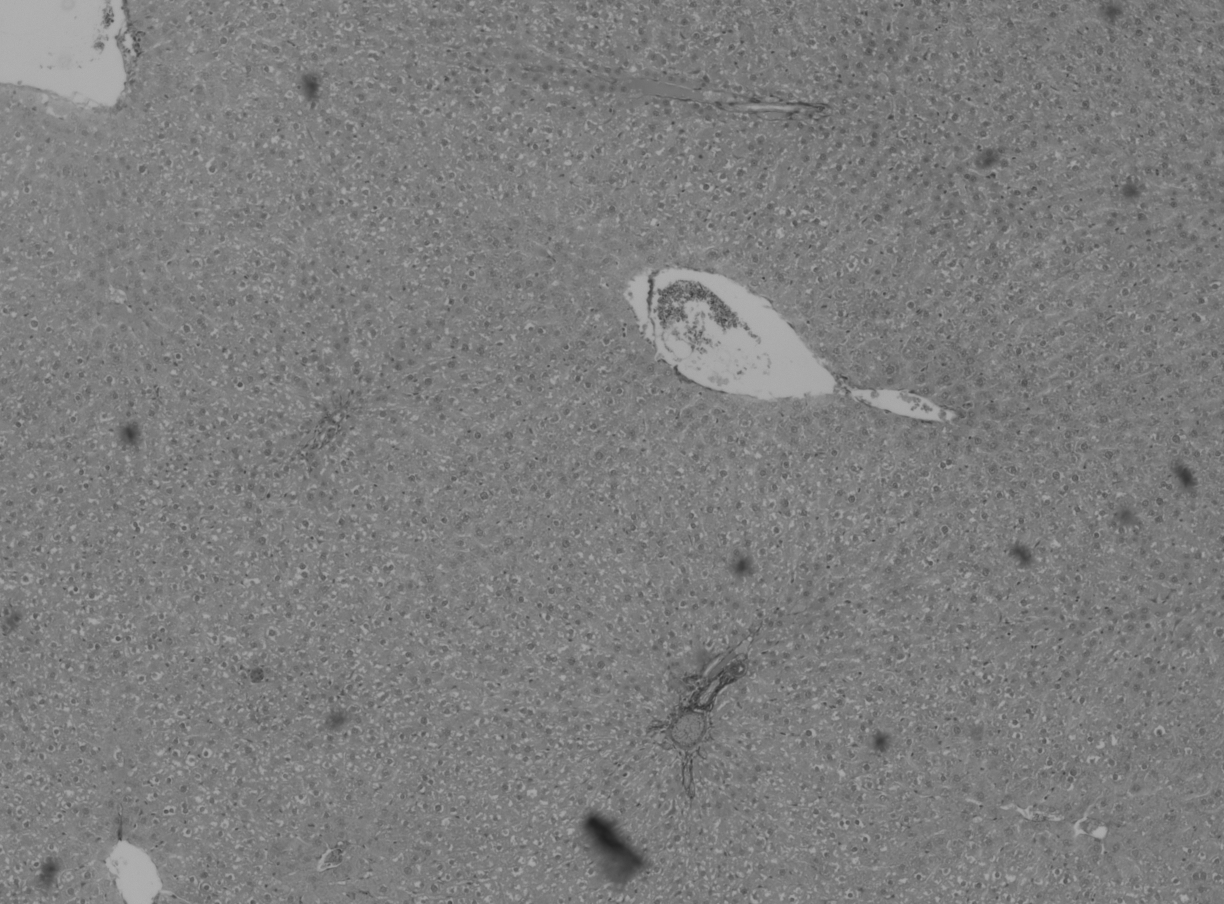

Supplement: S3 File — (ZIP) [file pone.0306020.s003.zip › ccl+fk506 6.tif]

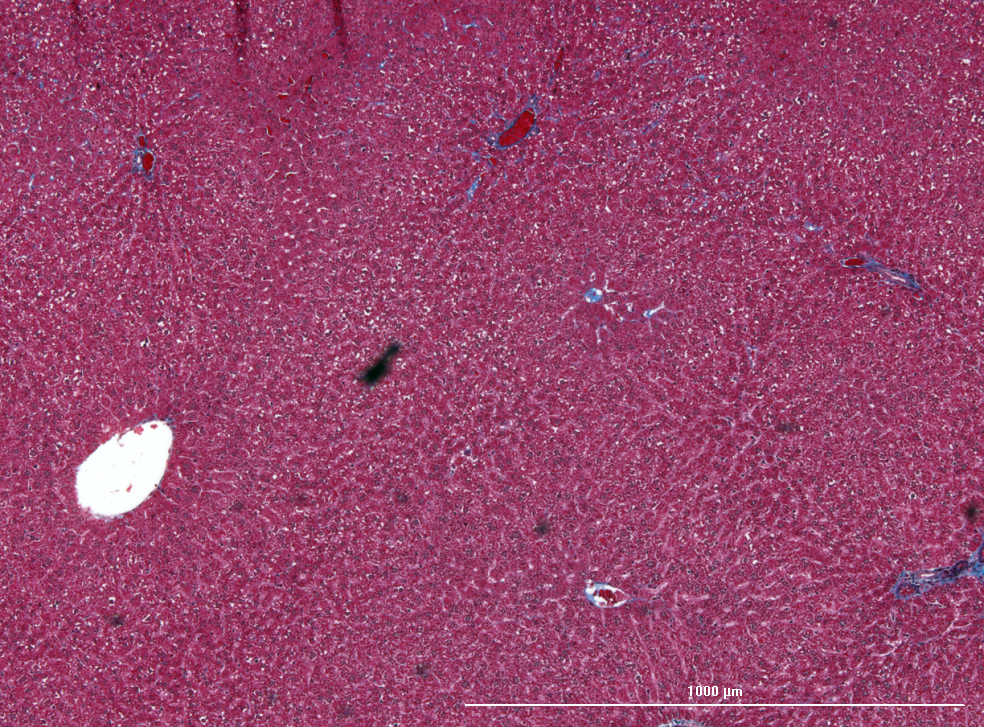

Supplement: S3 File — (ZIP) [file pone.0306020.s003.zip › ccl+fk506 7.jpg]

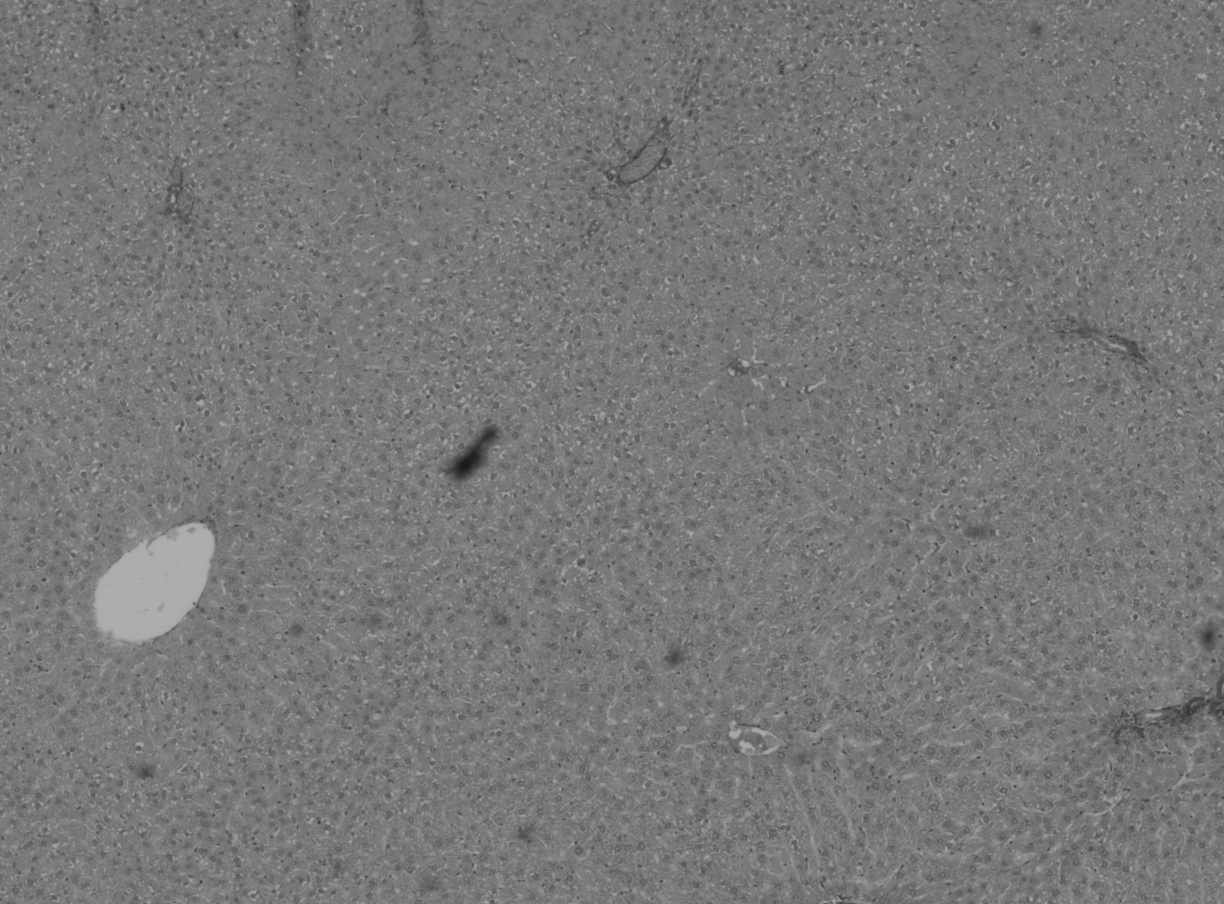

Supplement: S3 File — (ZIP) [file pone.0306020.s003.zip › ccl+fk506 7.tif]

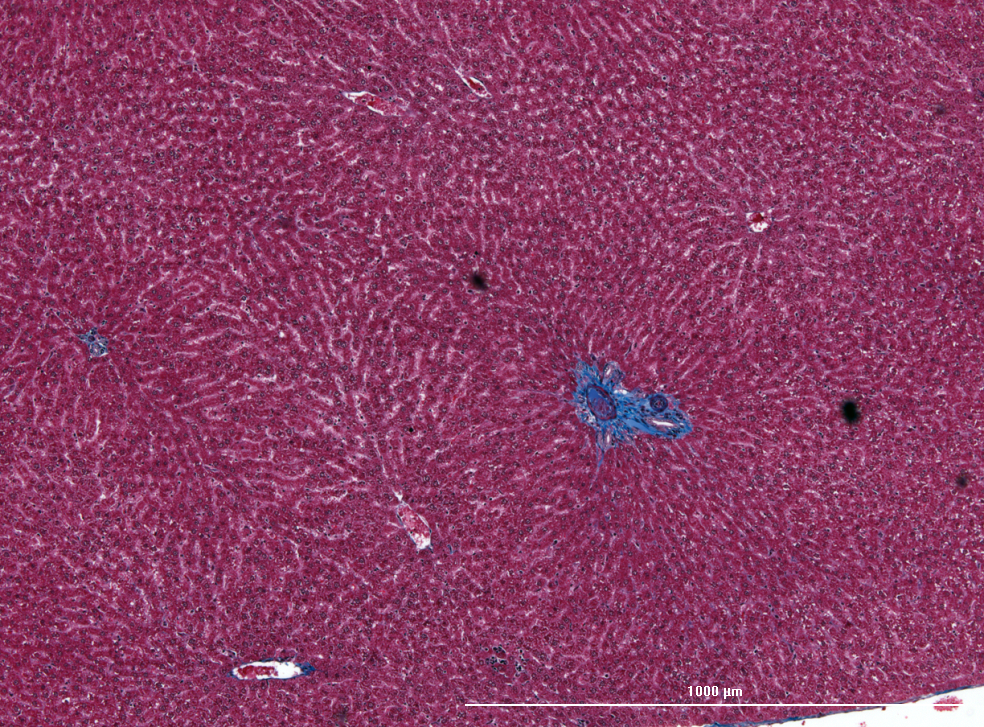

Supplement: S3 File — (ZIP) [file pone.0306020.s003.zip › ccl+fk506 8.jpg]

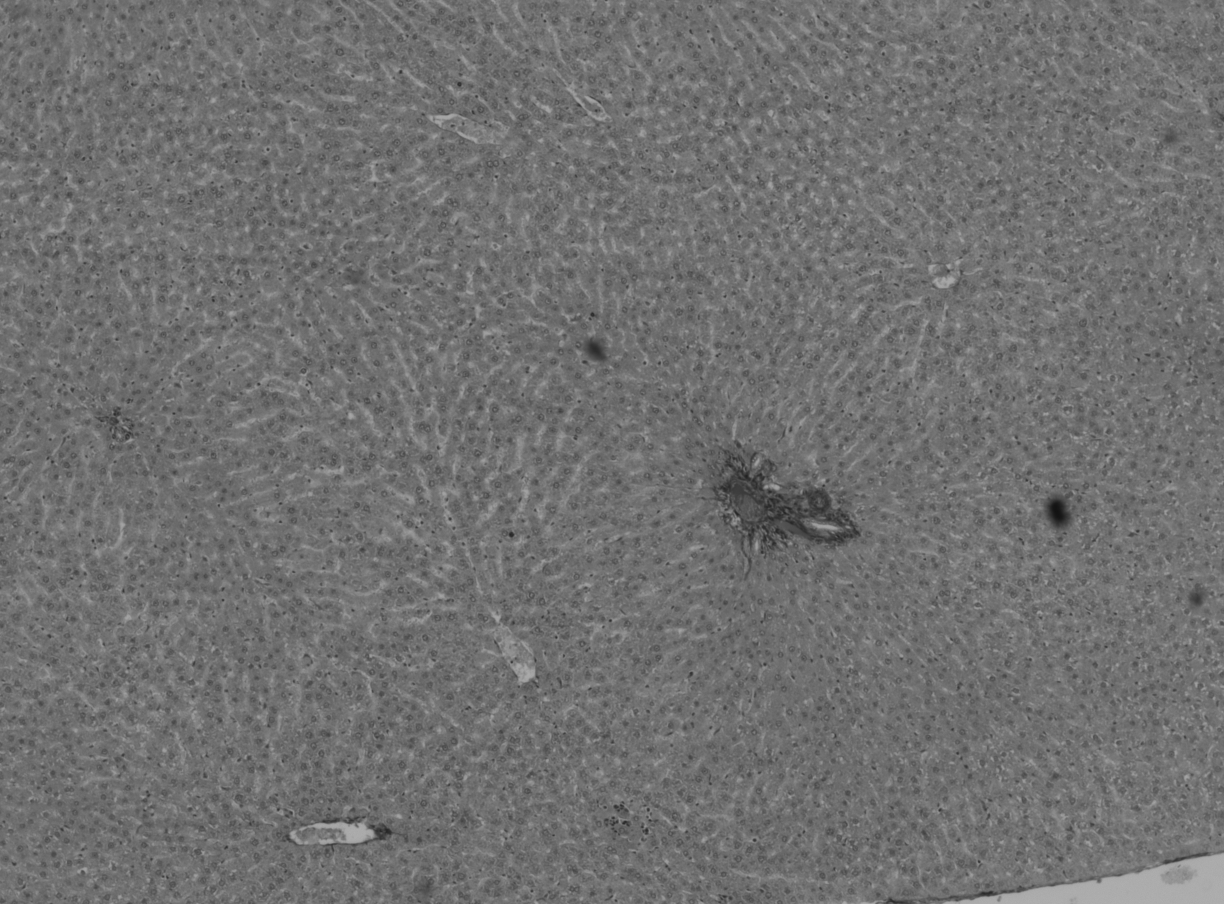

Supplement: S3 File — (ZIP) [file pone.0306020.s003.zip › ccl+fk506 8.tif]

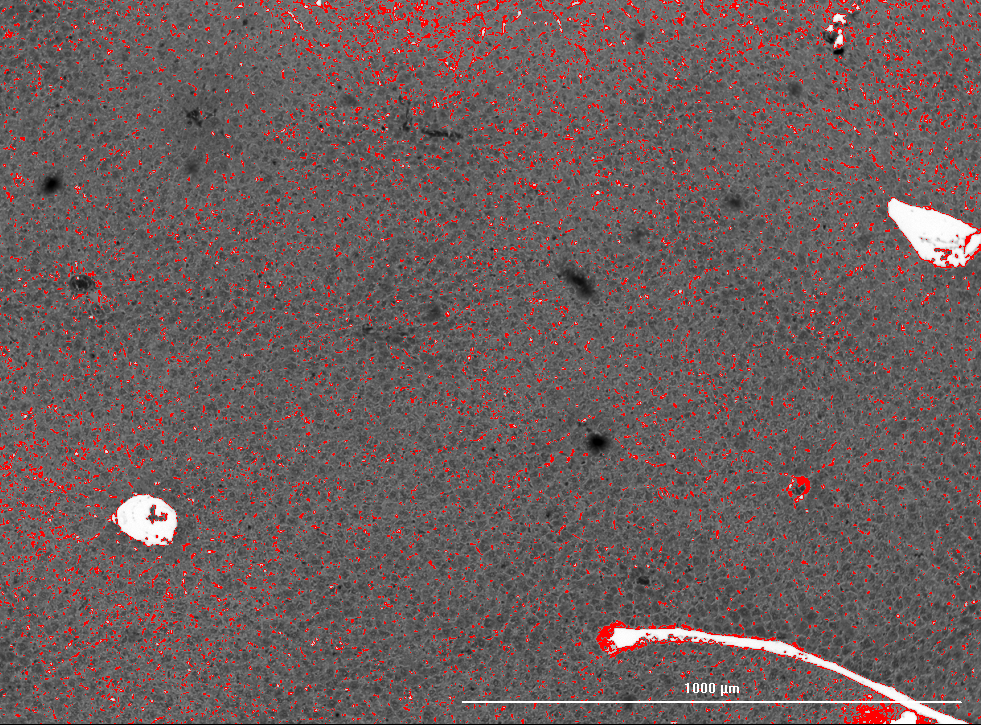

Supplement: S3 File — (ZIP) [file pone.0306020.s003.zip › ccl+fk506_1_bluesaturation.png]

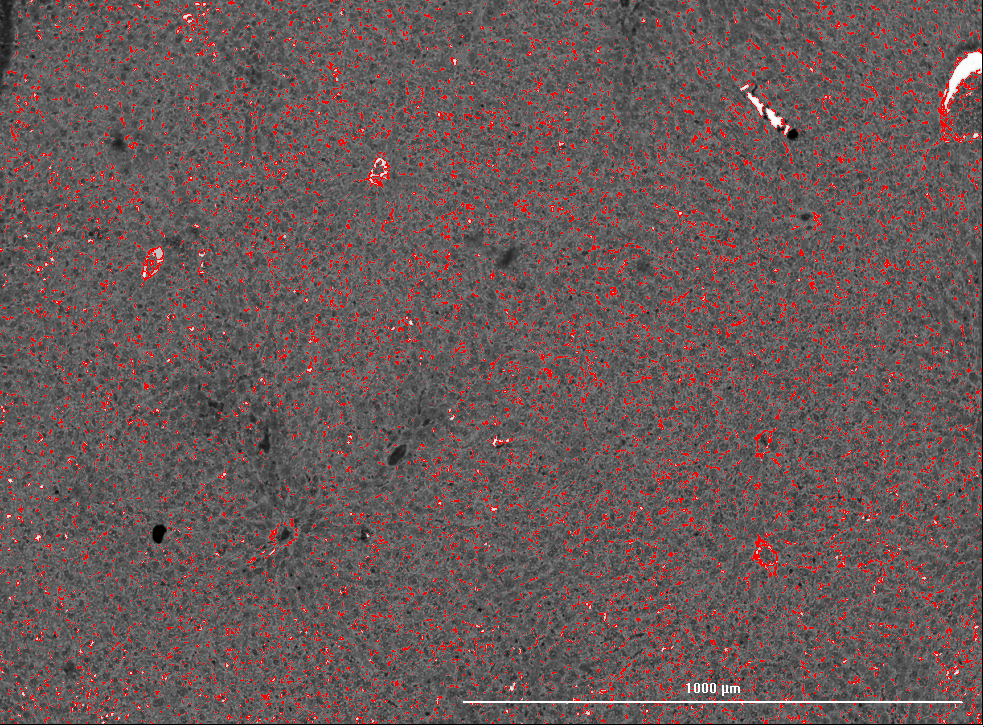

Supplement: S3 File — (ZIP) [file pone.0306020.s003.zip › ccl+fk506_2_bluesaturation.png]

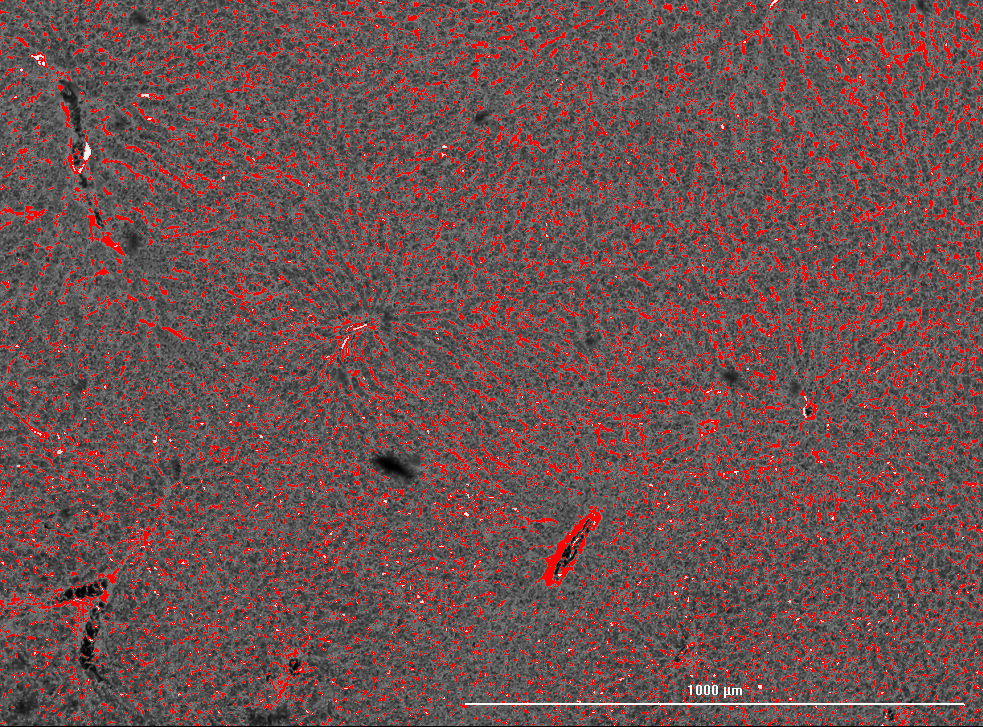

Supplement: S3 File — (ZIP) [file pone.0306020.s003.zip › ccl+fk506_3_bluesaturation.png]

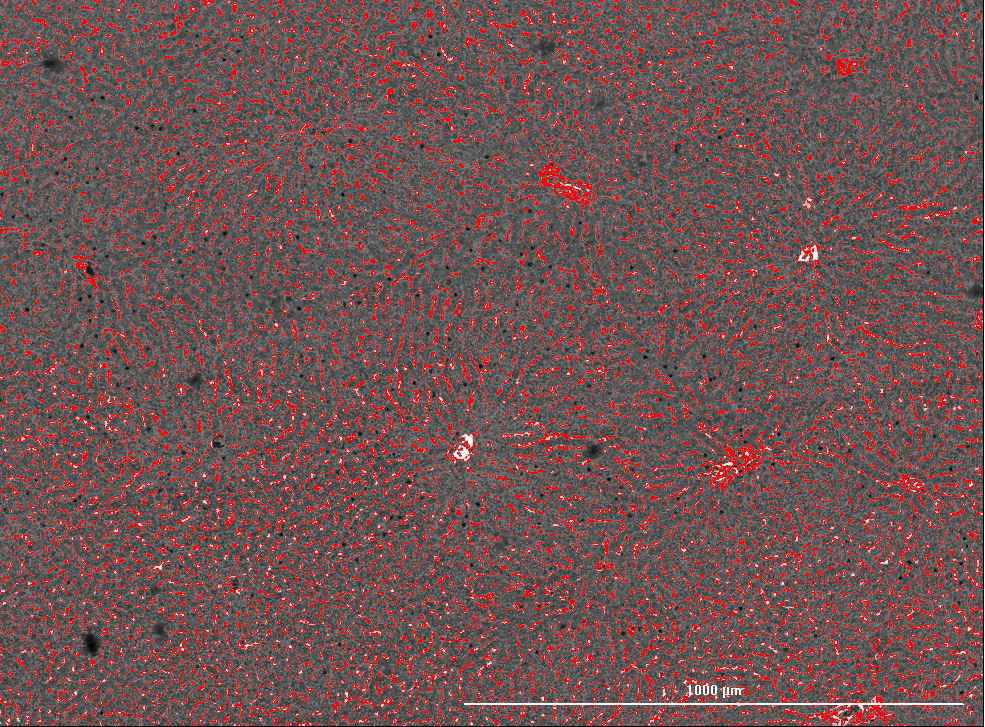

Supplement: S3 File — (ZIP) [file pone.0306020.s003.zip › ccl+fk506_4_bluesaturation.png]

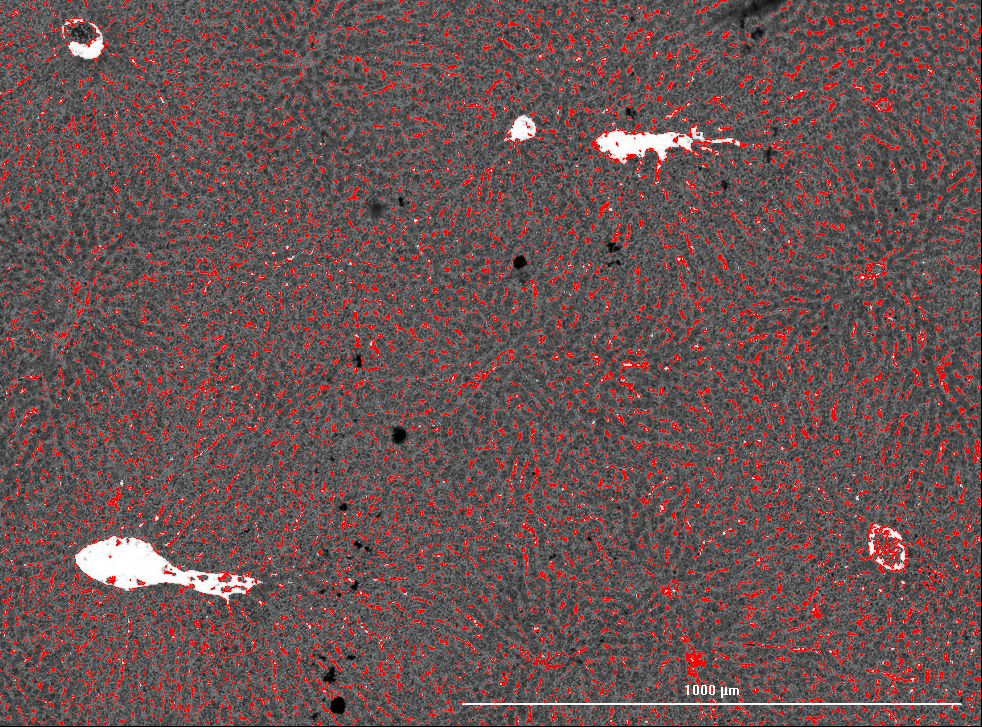

Supplement: S3 File — (ZIP) [file pone.0306020.s003.zip › ccl+fk506_5_bluesaturation.png]

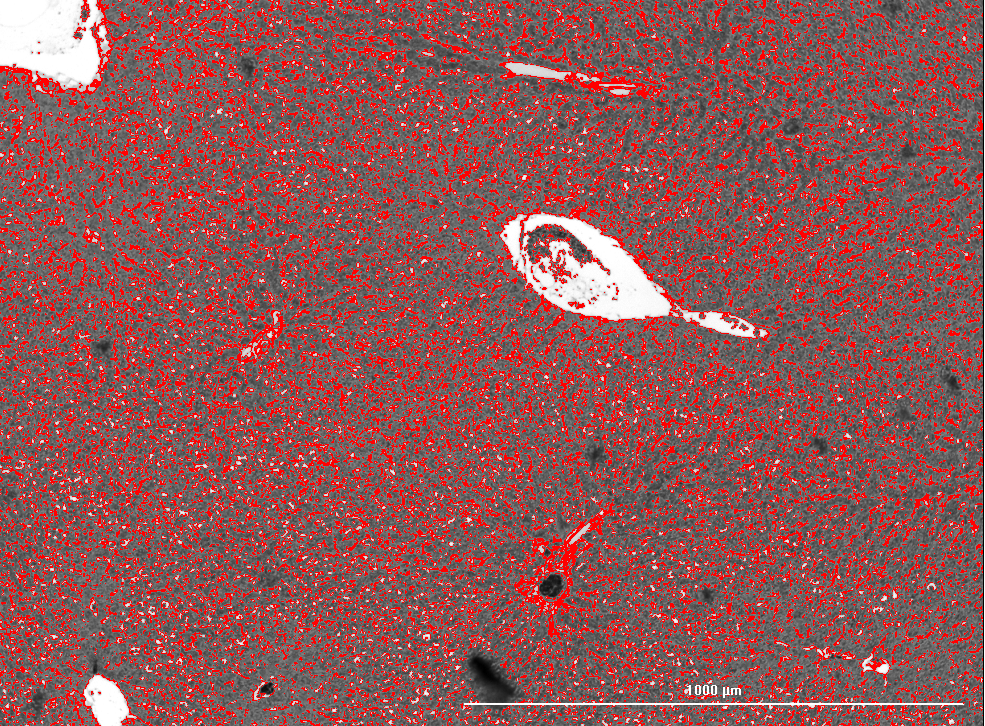

Supplement: S3 File — (ZIP) [file pone.0306020.s003.zip › ccl+fk506_6_bluesaturation.png]

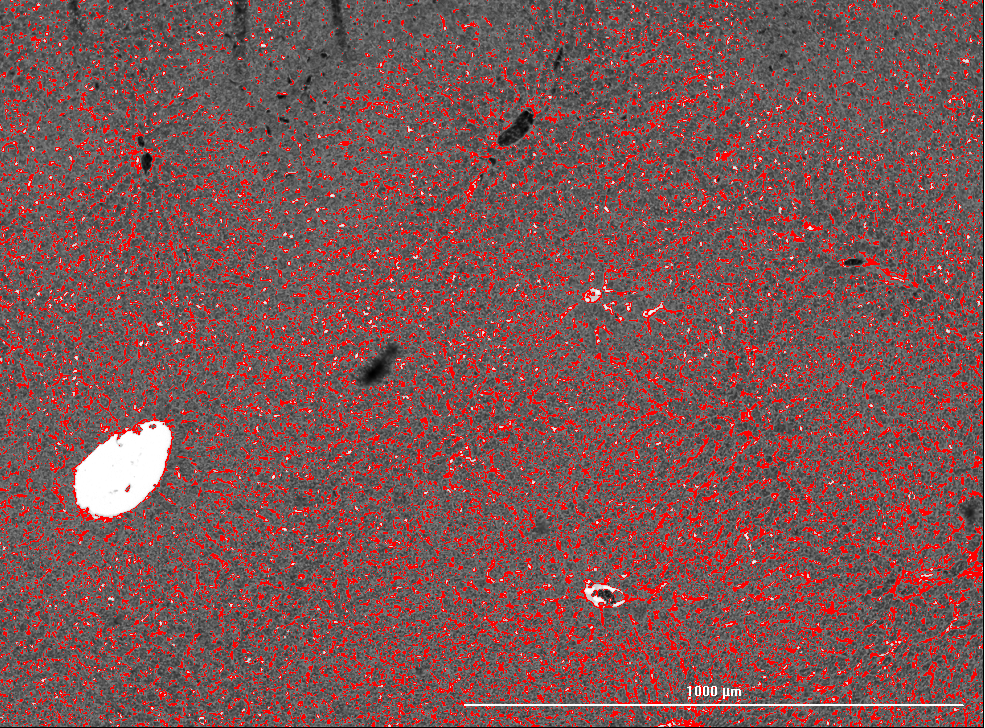

Supplement: S3 File — (ZIP) [file pone.0306020.s003.zip › ccl+fk506_7_bluesaturation.png]

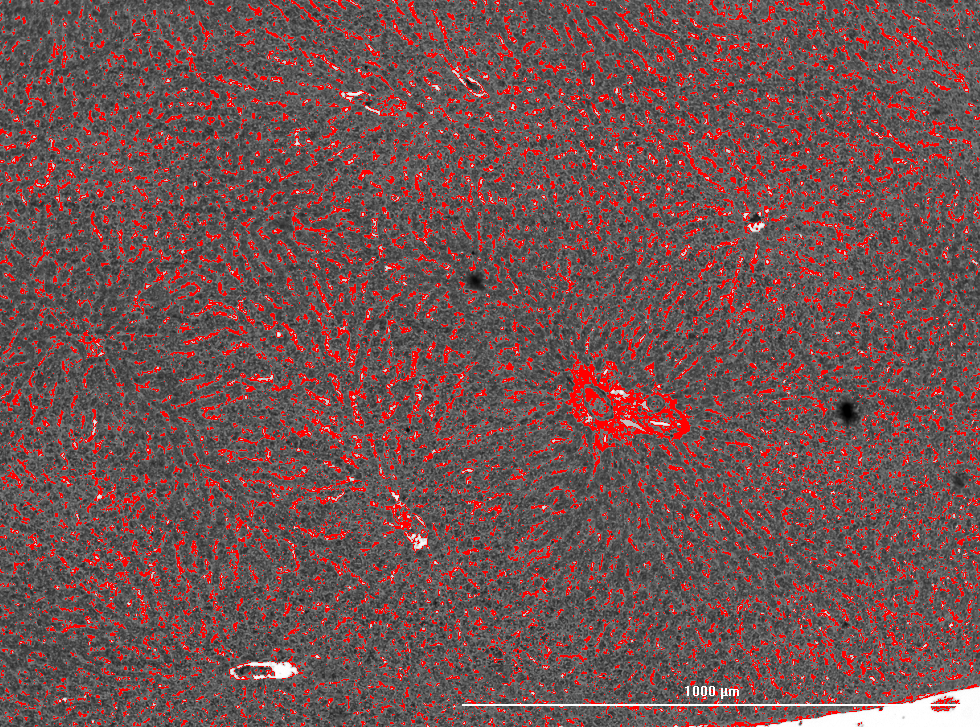

Supplement: S3 File — (ZIP) [file pone.0306020.s003.zip › ccl+fk506_8_bluesaturation.png]

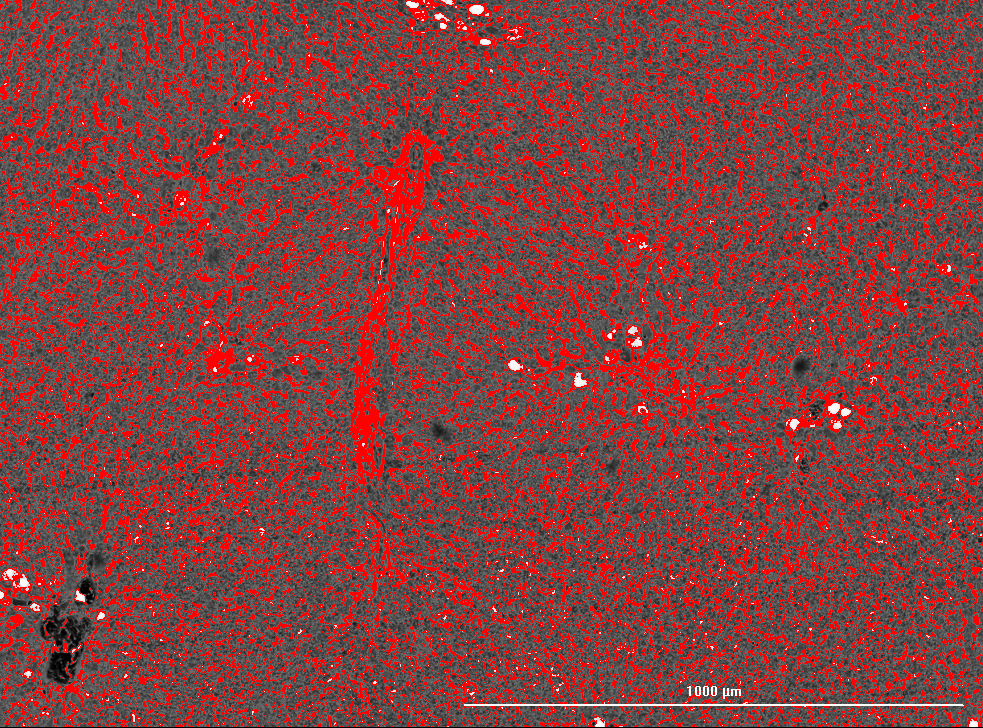

Supplement: S4 File — (ZIP) [file pone.0306020.s004.zip › ccl1_bluesaturation.png]

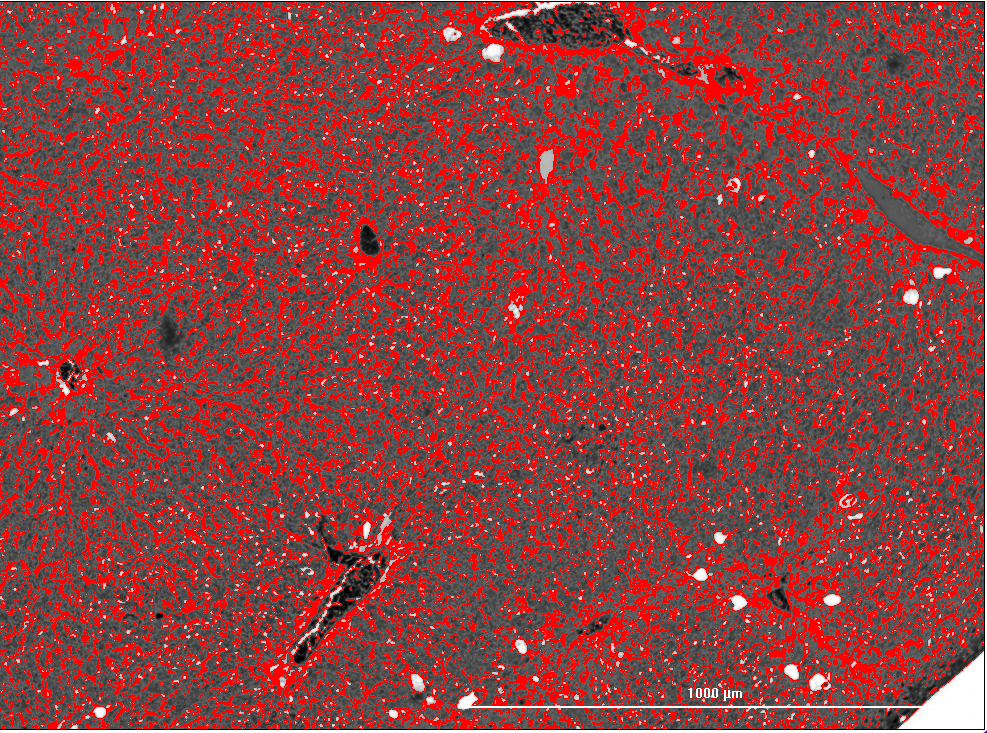

Supplement: S4 File — (ZIP) [file pone.0306020.s004.zip › ccl2_bluesaturation.png]

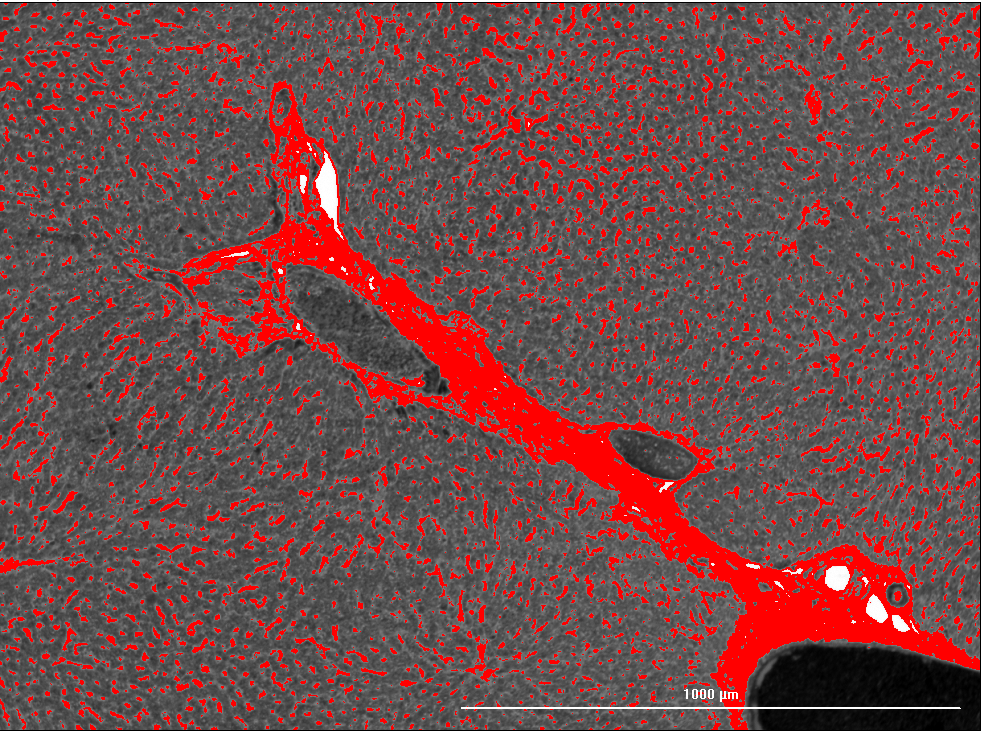

Supplement: S4 File — (ZIP) [file pone.0306020.s004.zip › ccl3_bluesaturation.png]

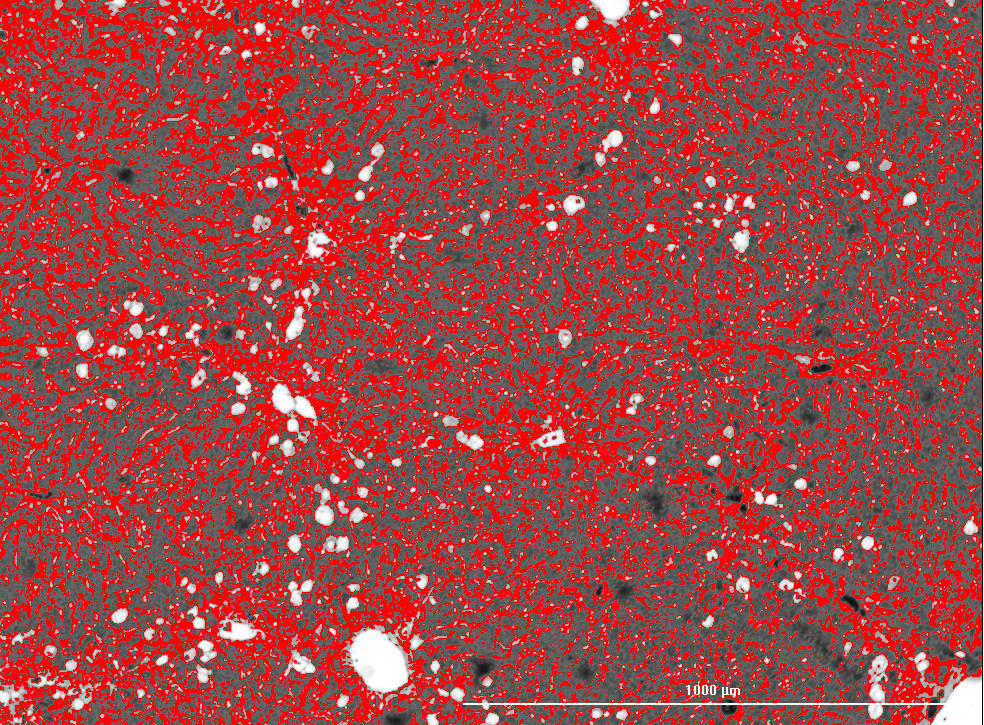

Supplement: S4 File — (ZIP) [file pone.0306020.s004.zip › ccl4_bluesaturation.png]

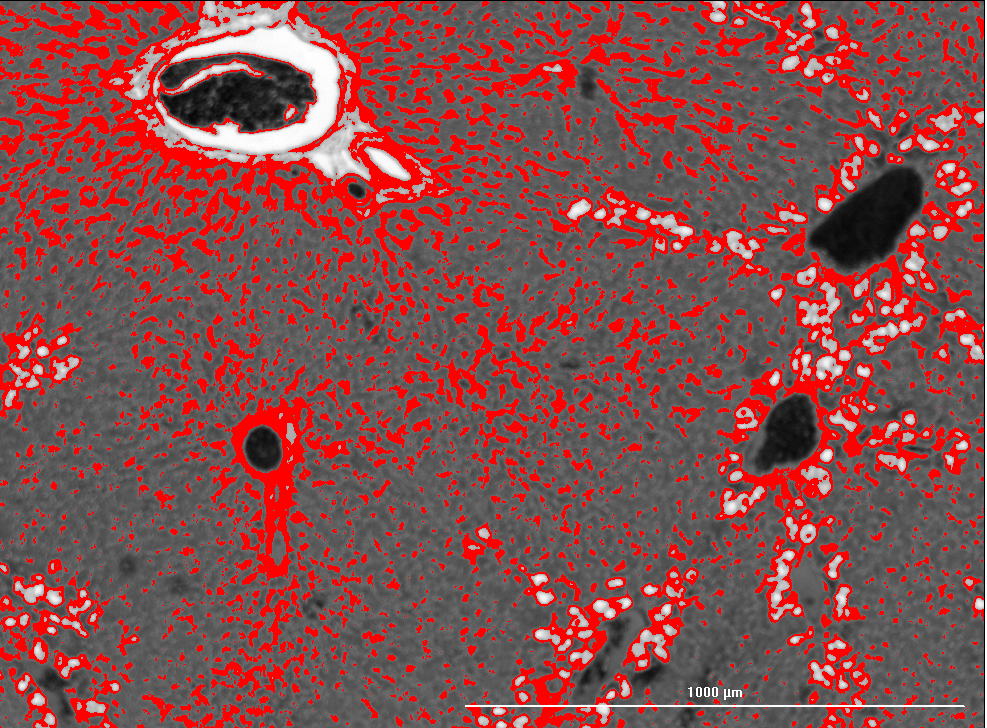

Supplement: S4 File — (ZIP) [file pone.0306020.s004.zip › ccl5_bluesaturation.png]

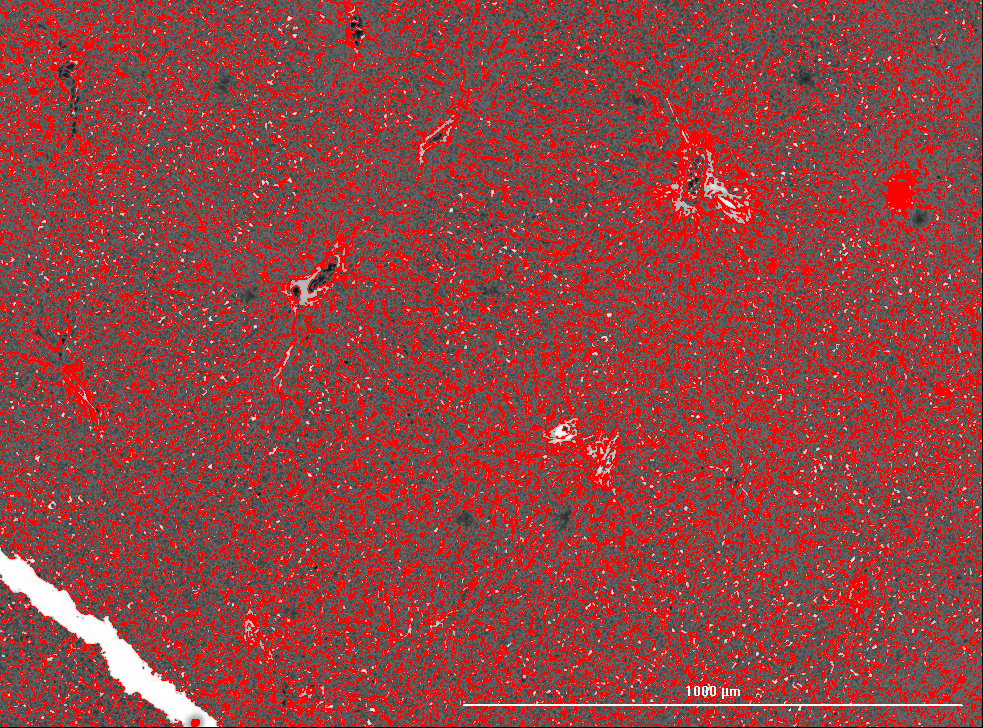

Supplement: S4 File — (ZIP) [file pone.0306020.s004.zip › ccl6_bluesaturation.png]

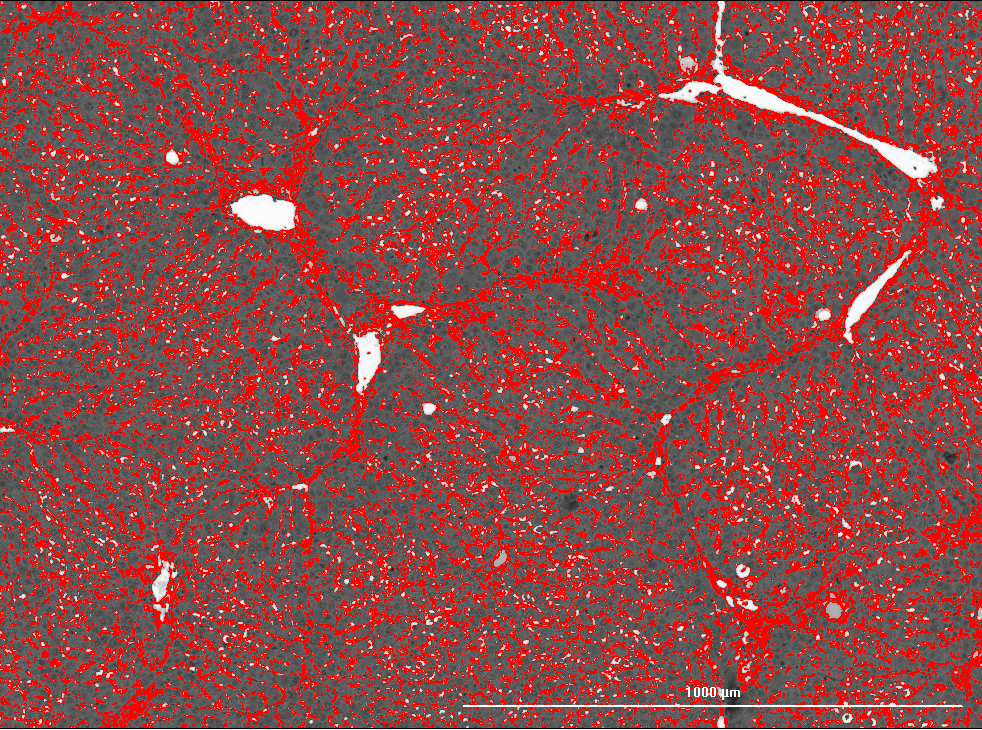

Supplement: S4 File — (ZIP) [file pone.0306020.s004.zip › ccl7_bluesaturation.png]

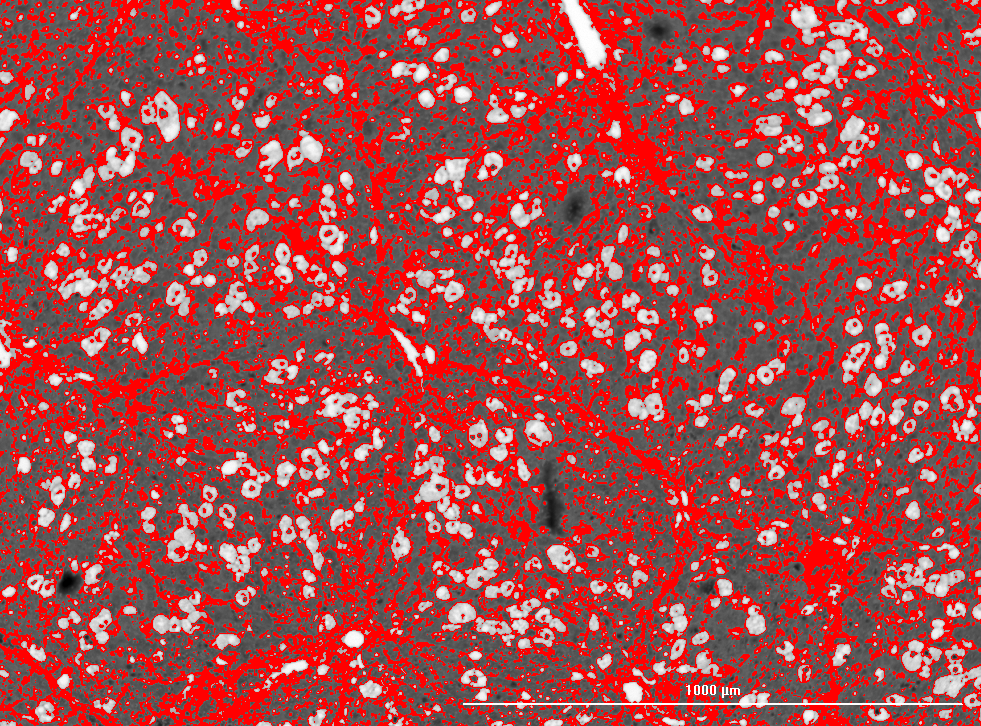

Supplement: S4 File — (ZIP) [file pone.0306020.s004.zip › ccl8_bluesaturation.png]

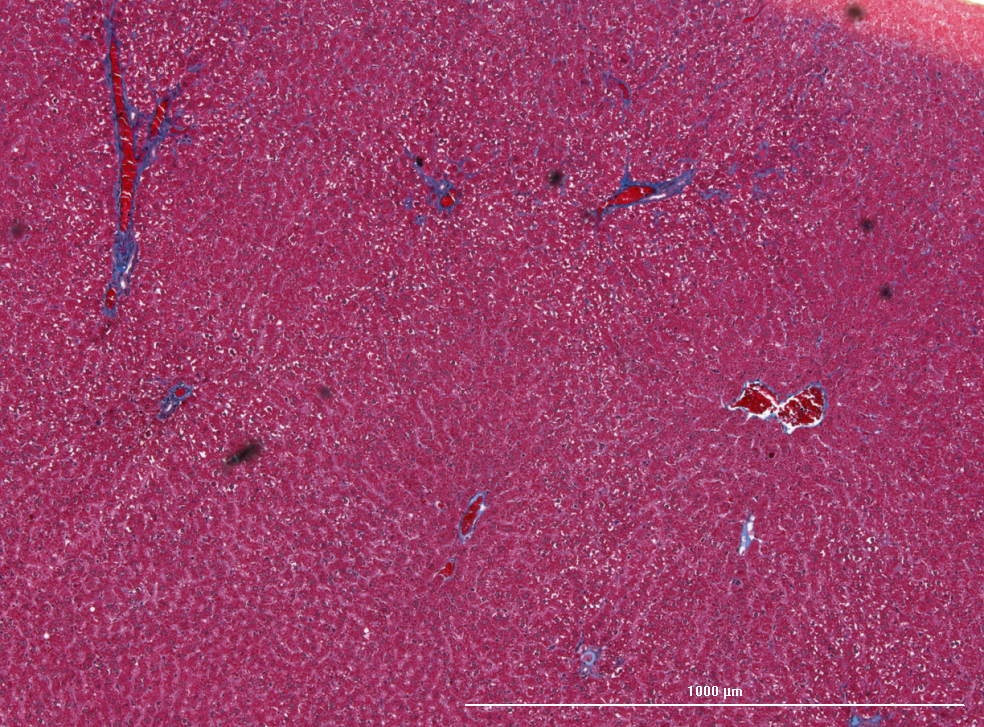

Supplement: S4 File — (ZIP) [file pone.0306020.s004.zip › fk506 1.jpg]

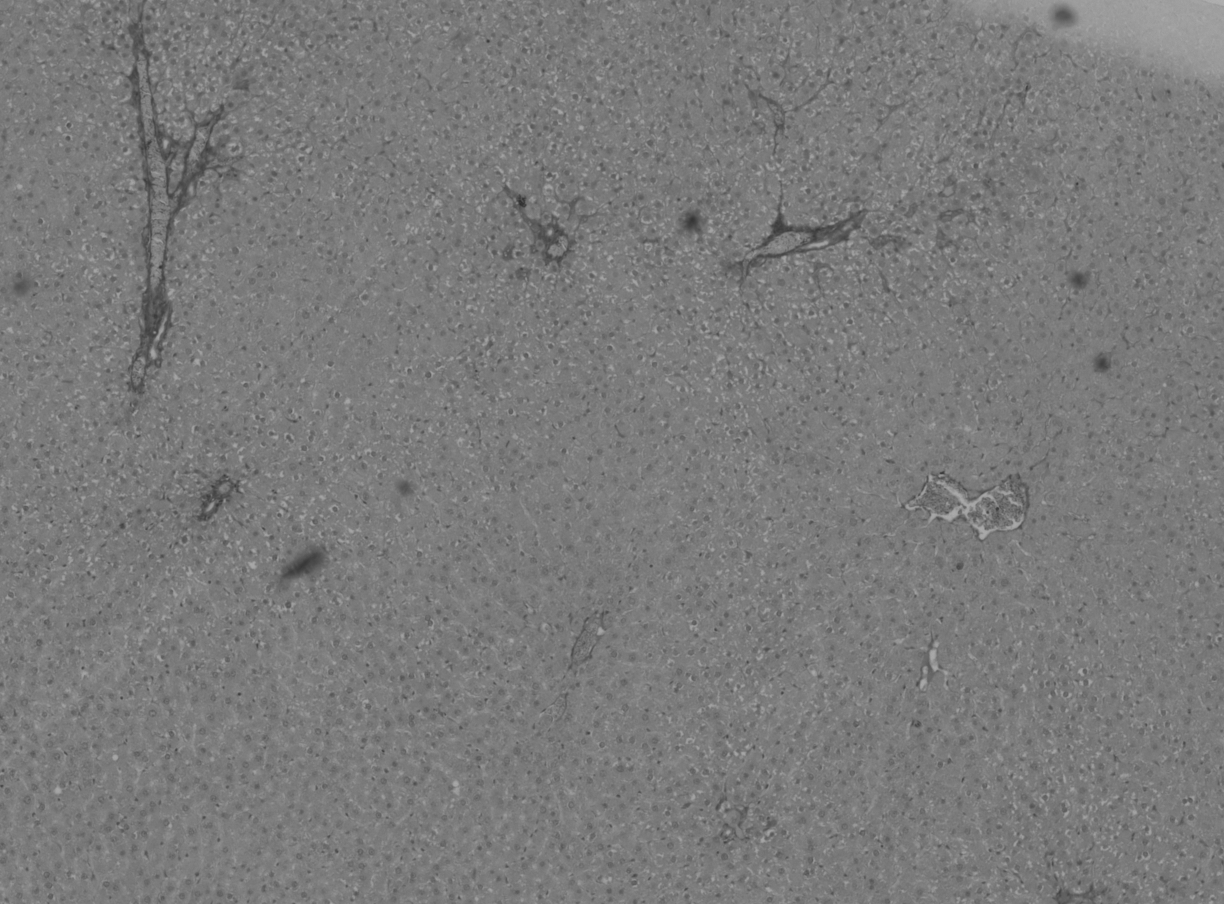

Supplement: S4 File — (ZIP) [file pone.0306020.s004.zip › fk506 1.tif]

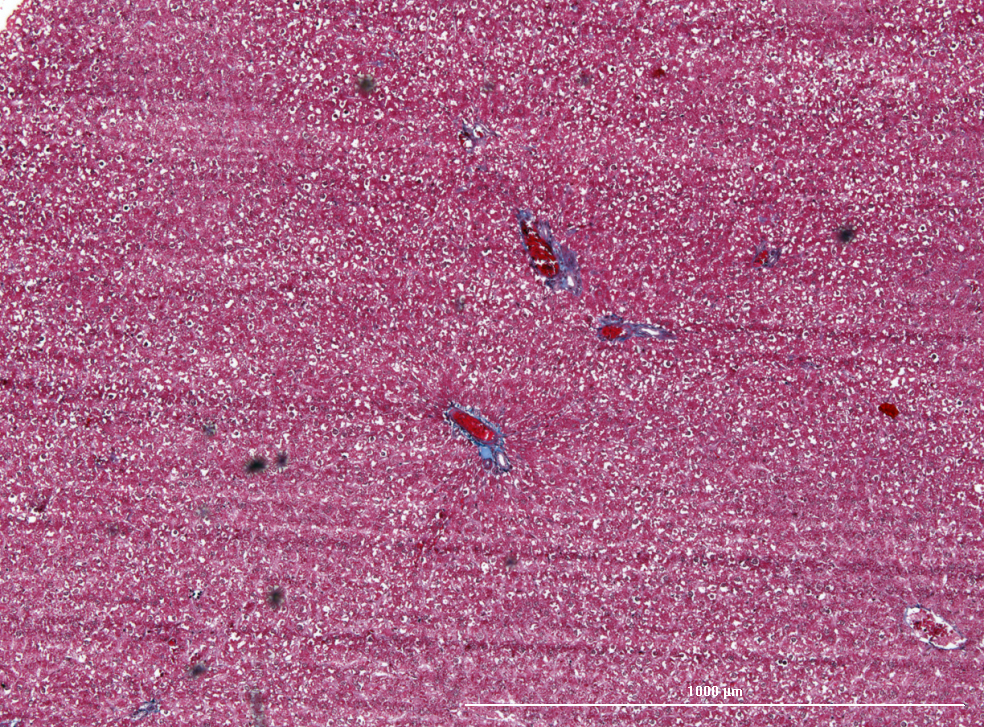

Supplement: S4 File — (ZIP) [file pone.0306020.s004.zip › fk506 2.jpg]

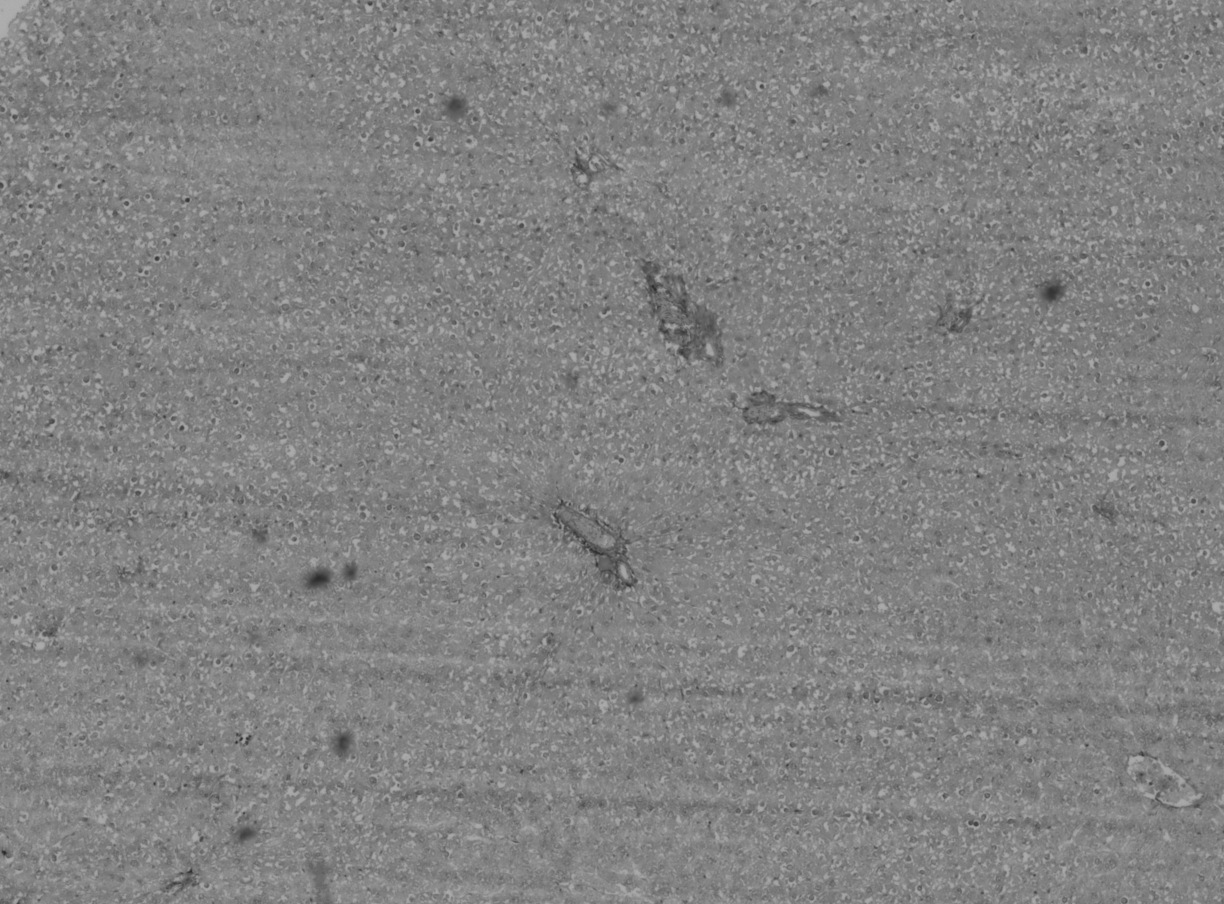

Supplement: S4 File — (ZIP) [file pone.0306020.s004.zip › fk506 2.tif]

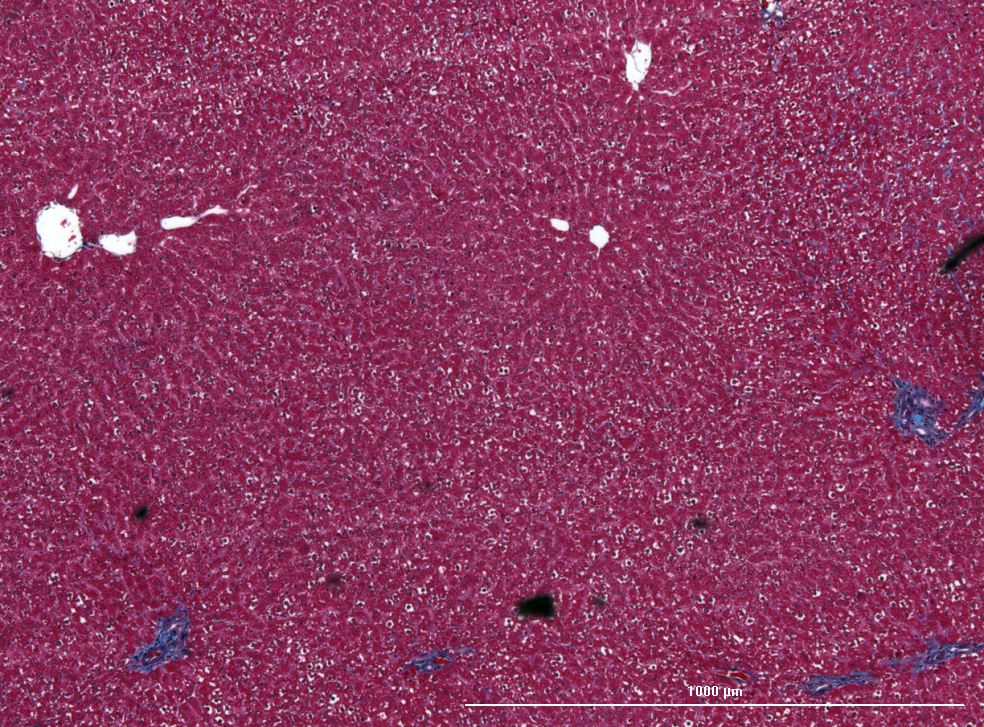

Supplement: S4 File — (ZIP) [file pone.0306020.s004.zip › fk506 3.jpg]

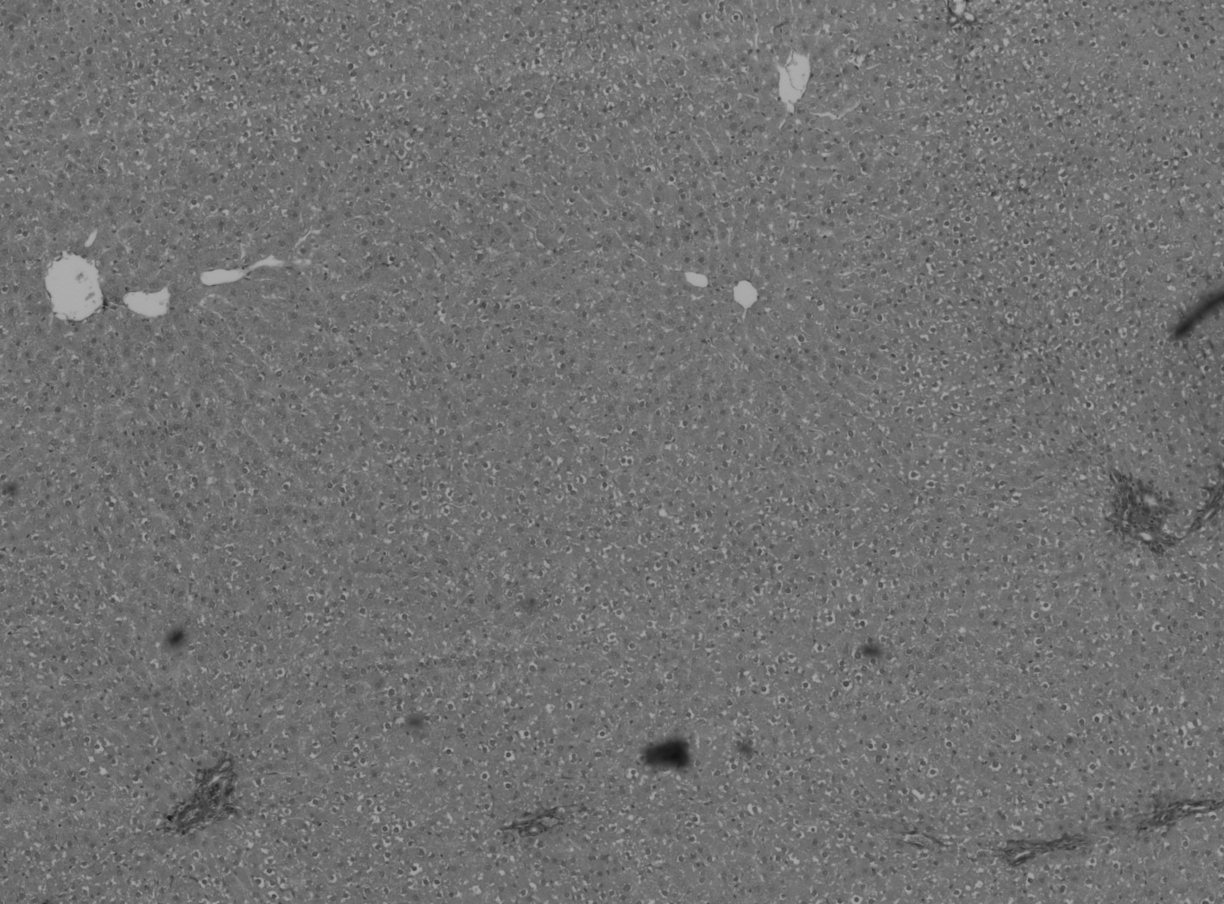

Supplement: S4 File — (ZIP) [file pone.0306020.s004.zip › fk506 3.tif]

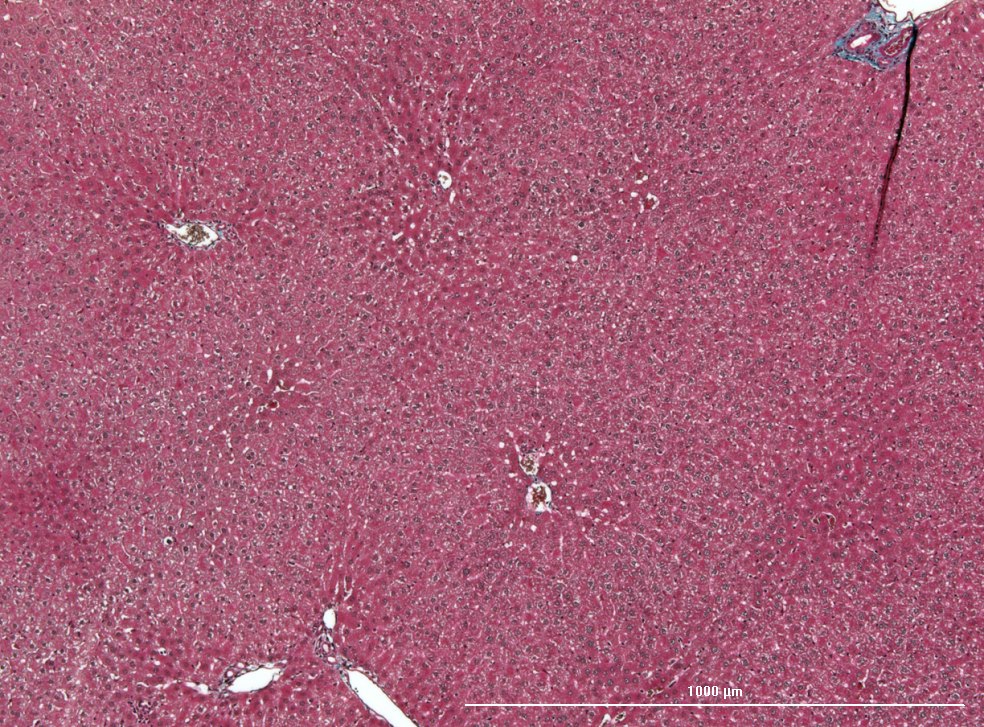

Supplement: S4 File — (ZIP) [file pone.0306020.s004.zip › fk506 4.jpg]

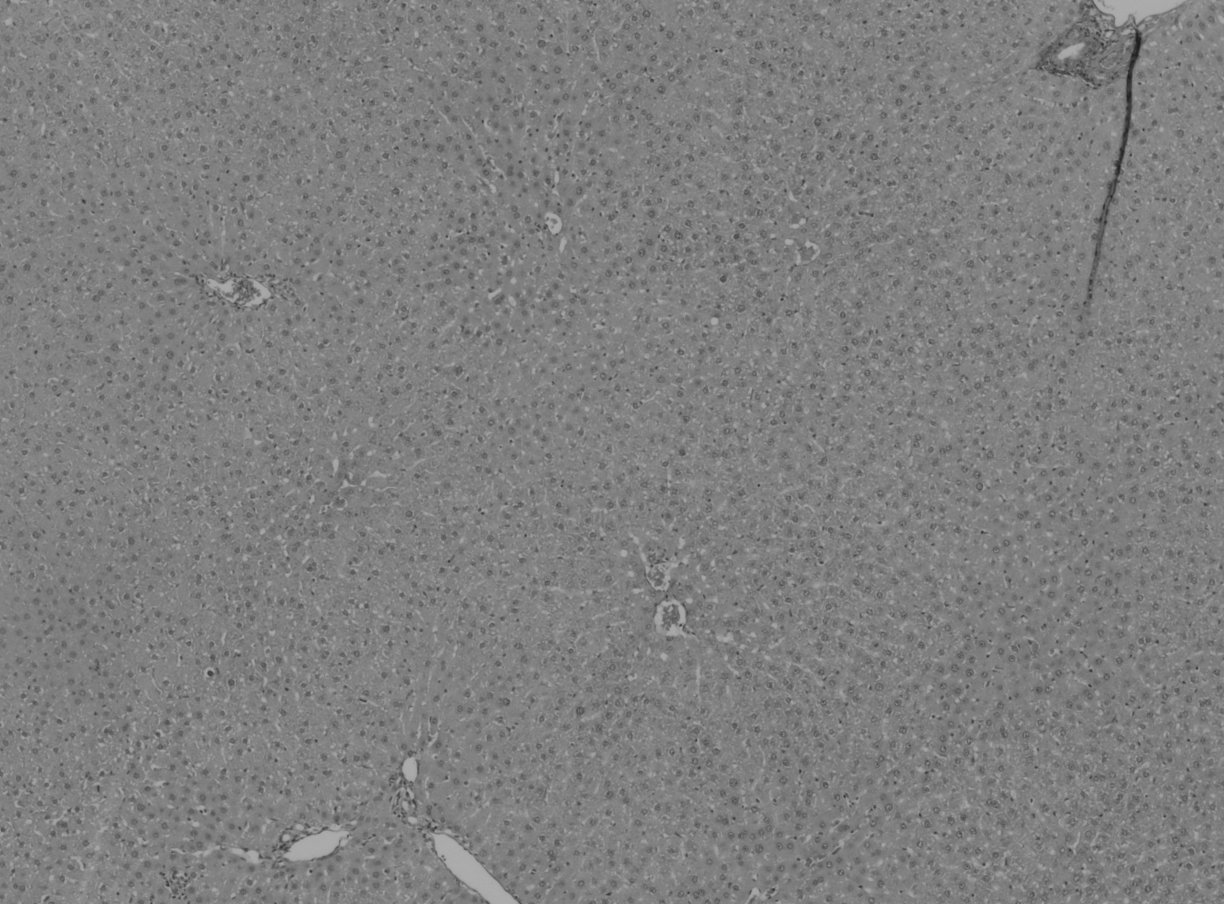

Supplement: S4 File — (ZIP) [file pone.0306020.s004.zip › fk506 4.tif]

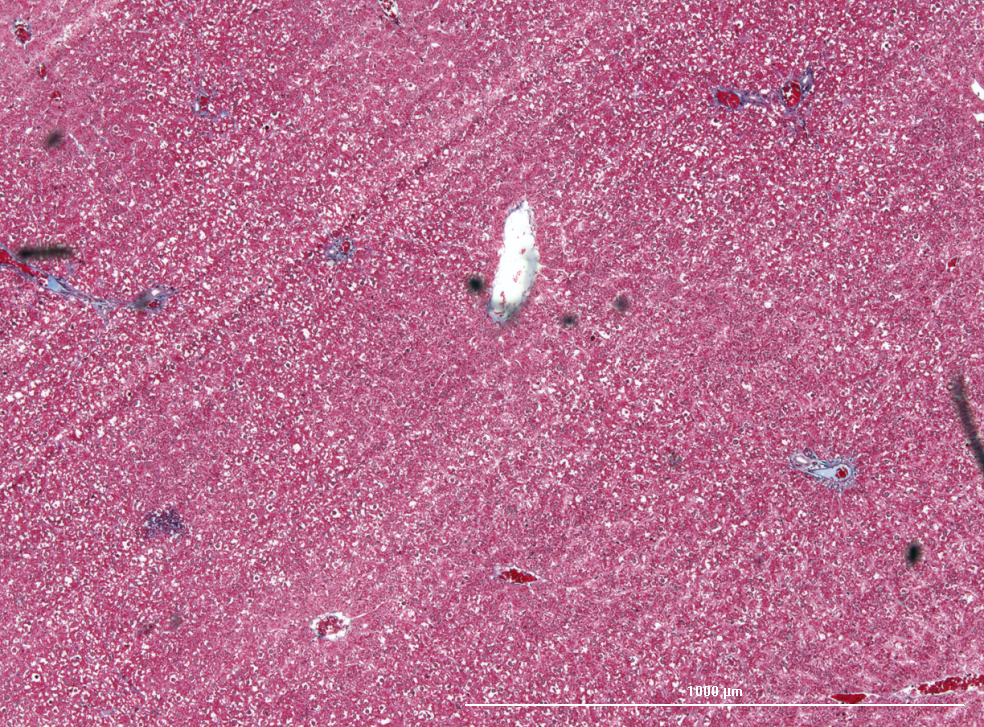

Supplement: S4 File — (ZIP) [file pone.0306020.s004.zip › fk506 5.jpg]

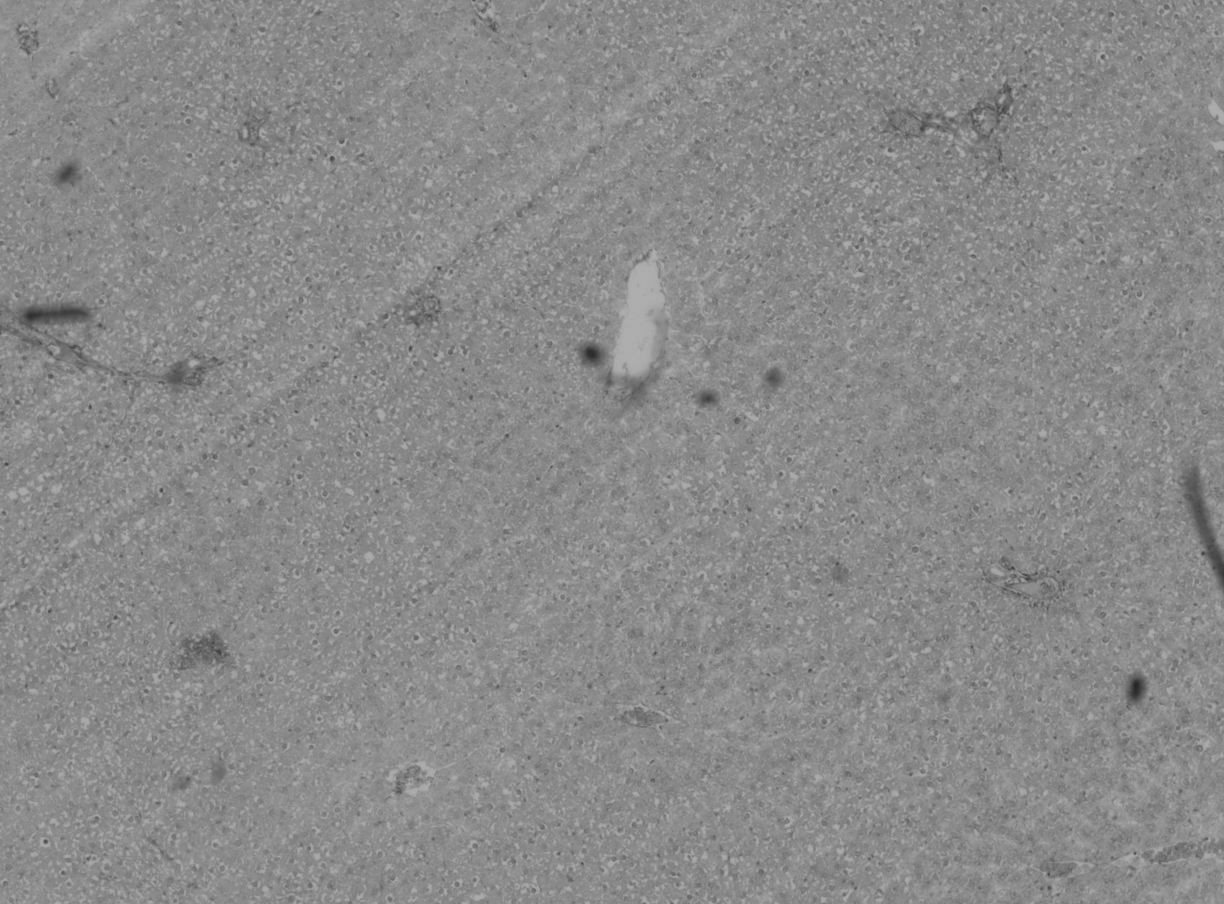

Supplement: S4 File — (ZIP) [file pone.0306020.s004.zip › fk506 5.tif]

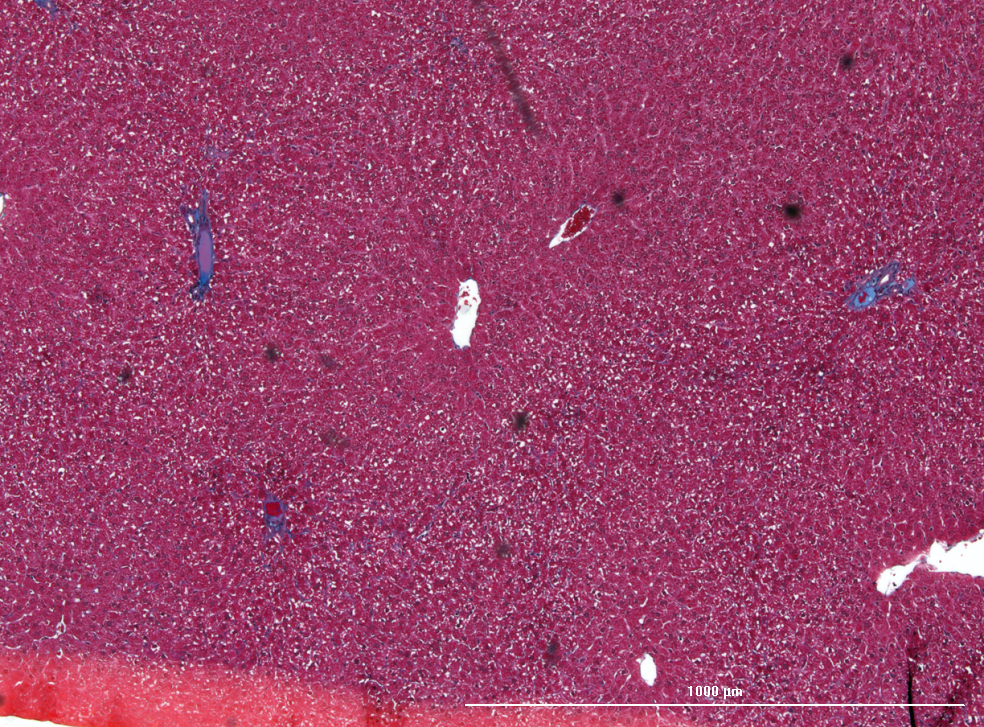

Supplement: S4 File — (ZIP) [file pone.0306020.s004.zip › fk506 6.jpg]

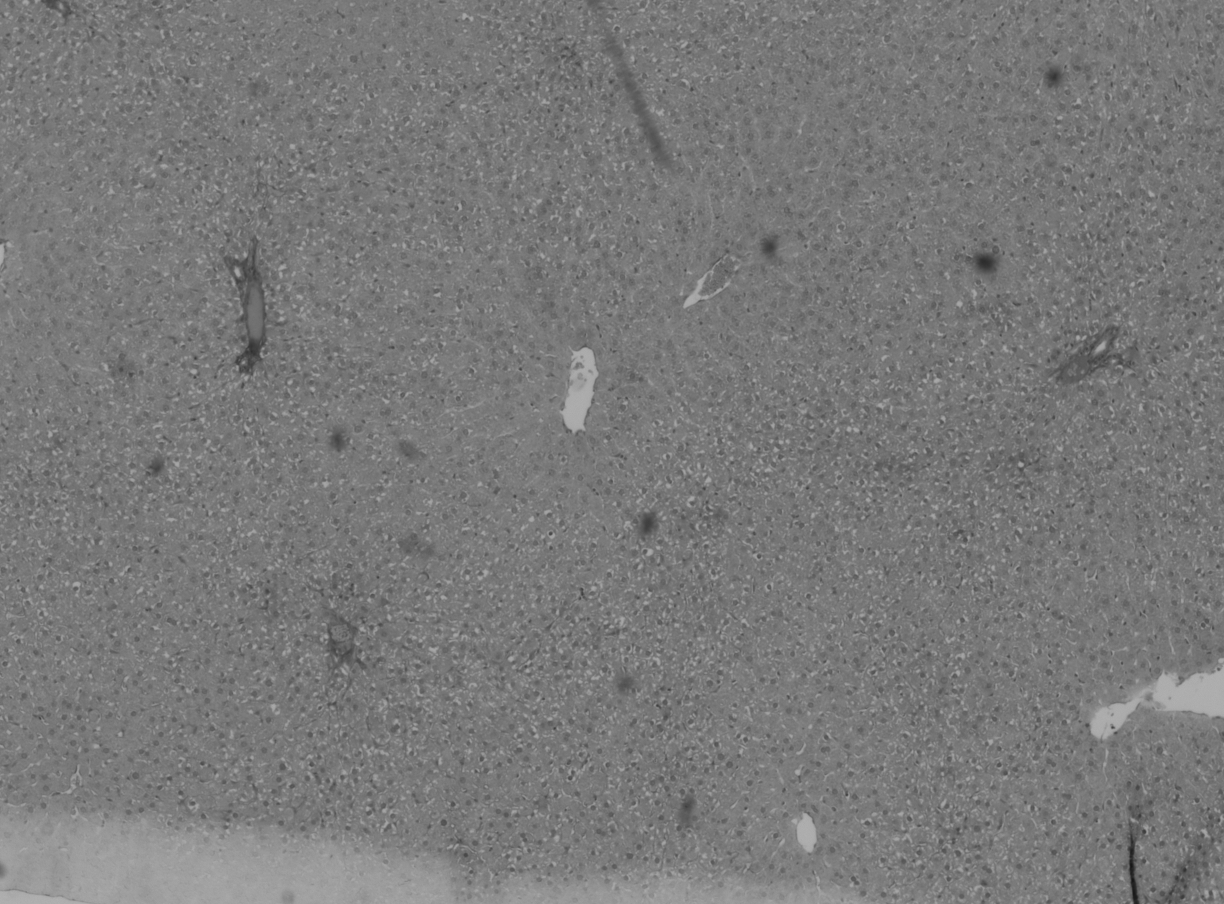

Supplement: S4 File — (ZIP) [file pone.0306020.s004.zip › fk506 6.tif]

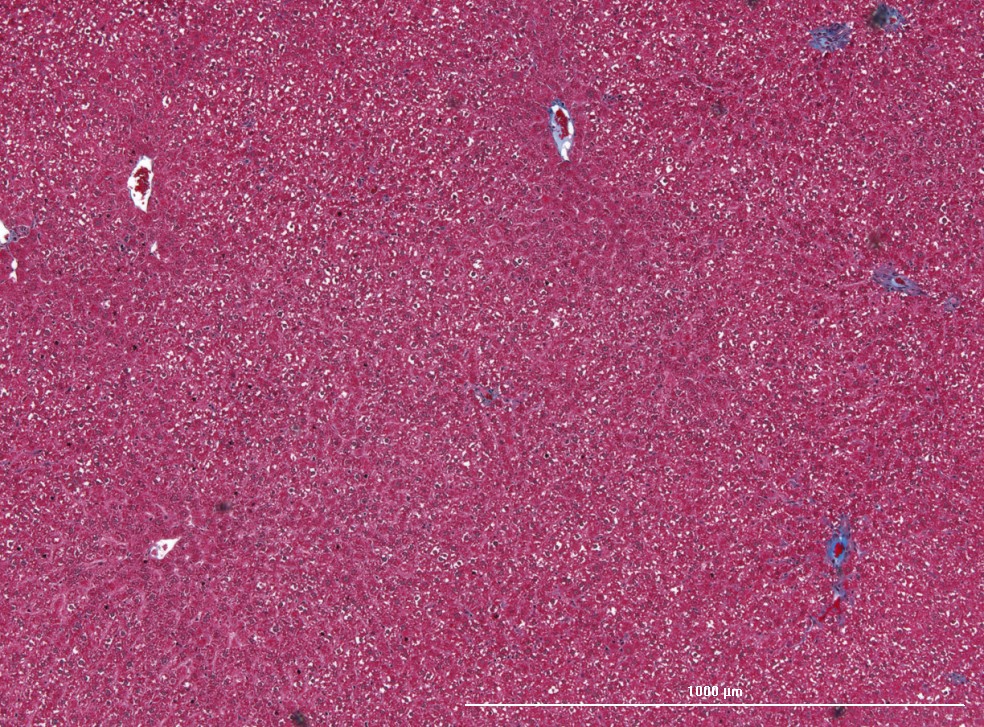

Supplement: S4 File — (ZIP) [file pone.0306020.s004.zip › fk506 7.jpg]

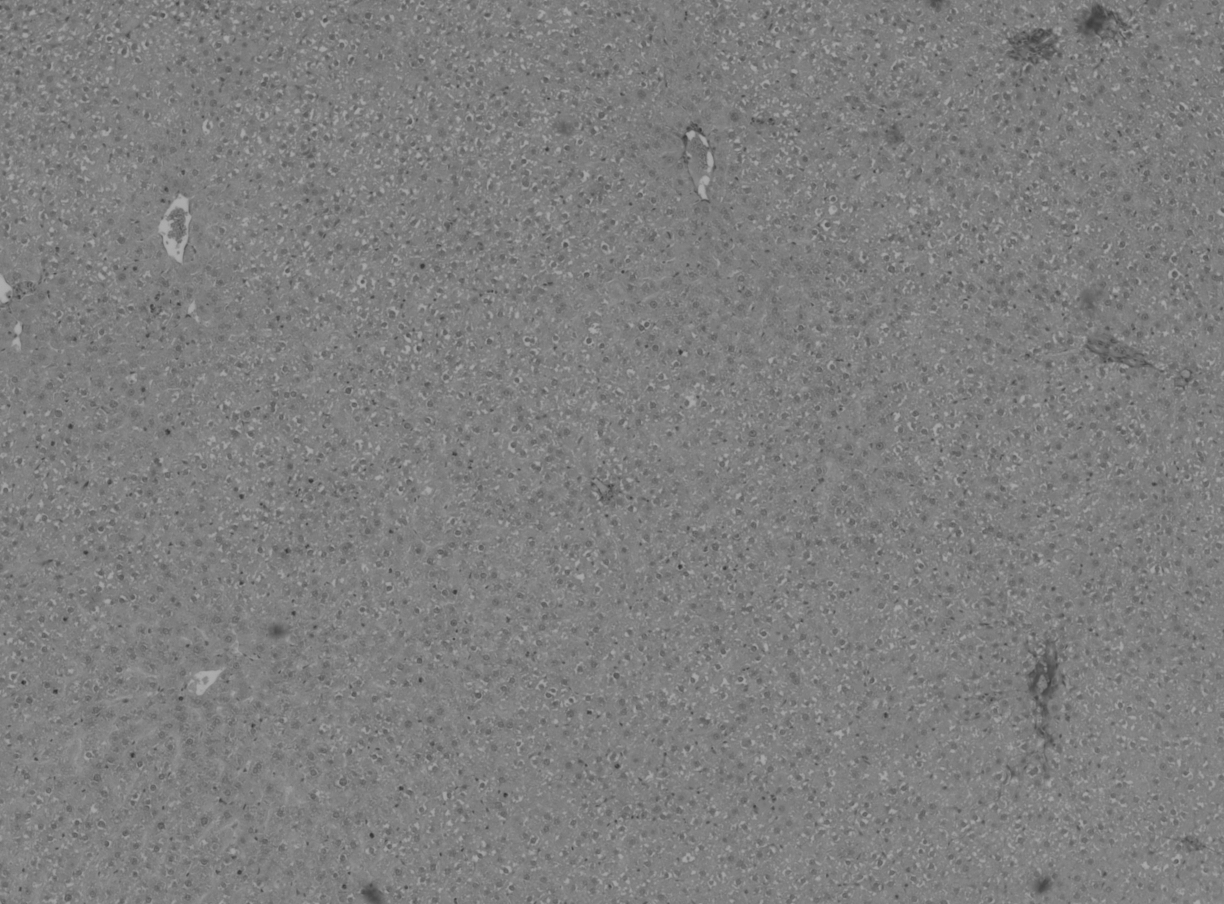

Supplement: S4 File — (ZIP) [file pone.0306020.s004.zip › fk506 7.tif]

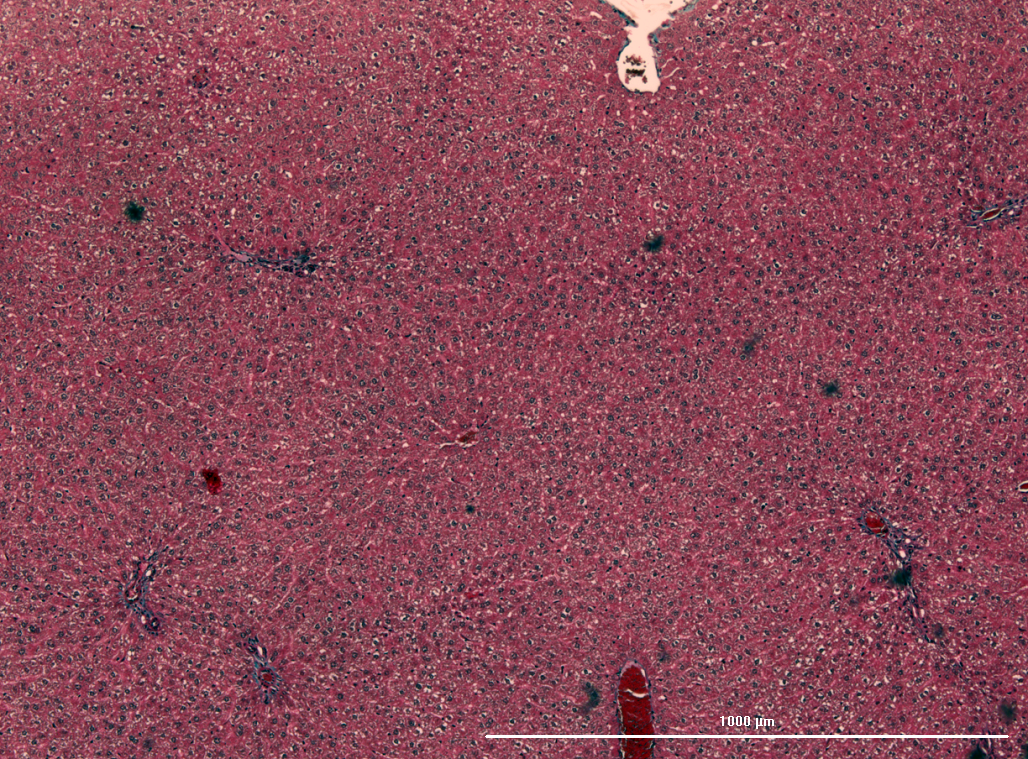

Supplement: S4 File — (ZIP) [file pone.0306020.s004.zip › fk506 8.jpg]

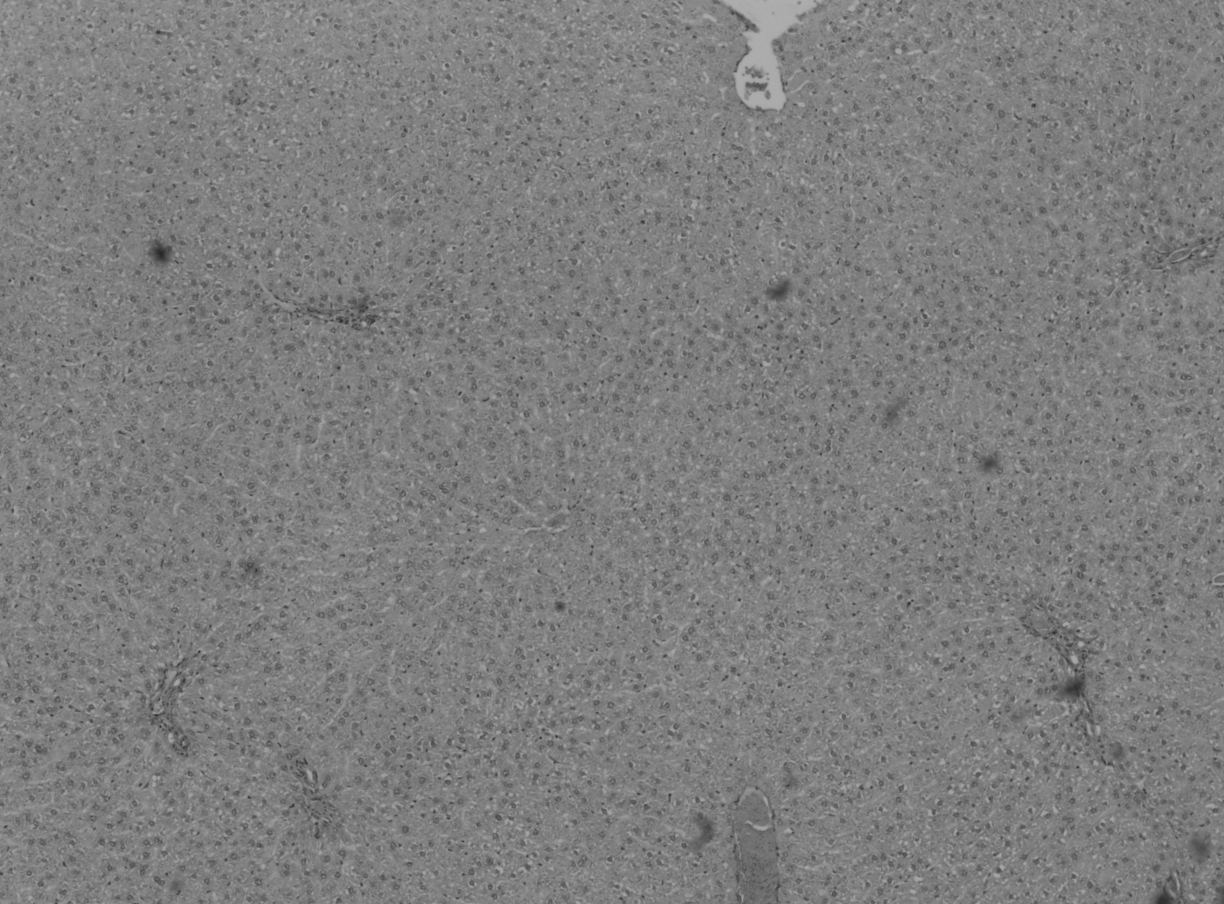

Supplement: S4 File — (ZIP) [file pone.0306020.s004.zip › fk506 8.tif]

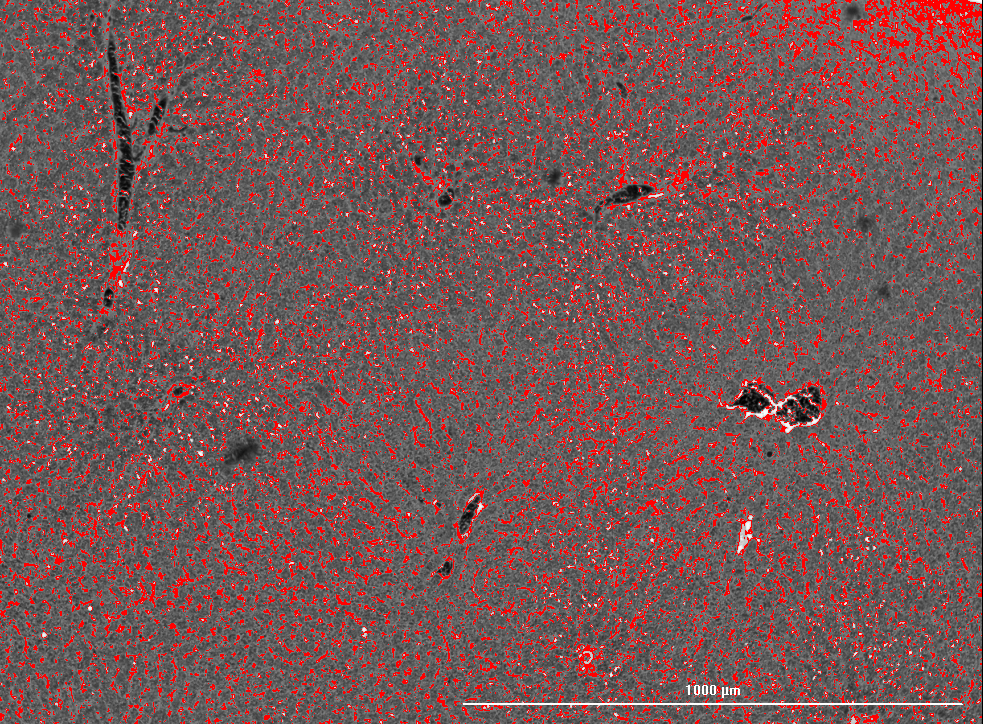

Supplement: S4 File — (ZIP) [file pone.0306020.s004.zip › fk506_1_bluesaturation.png]

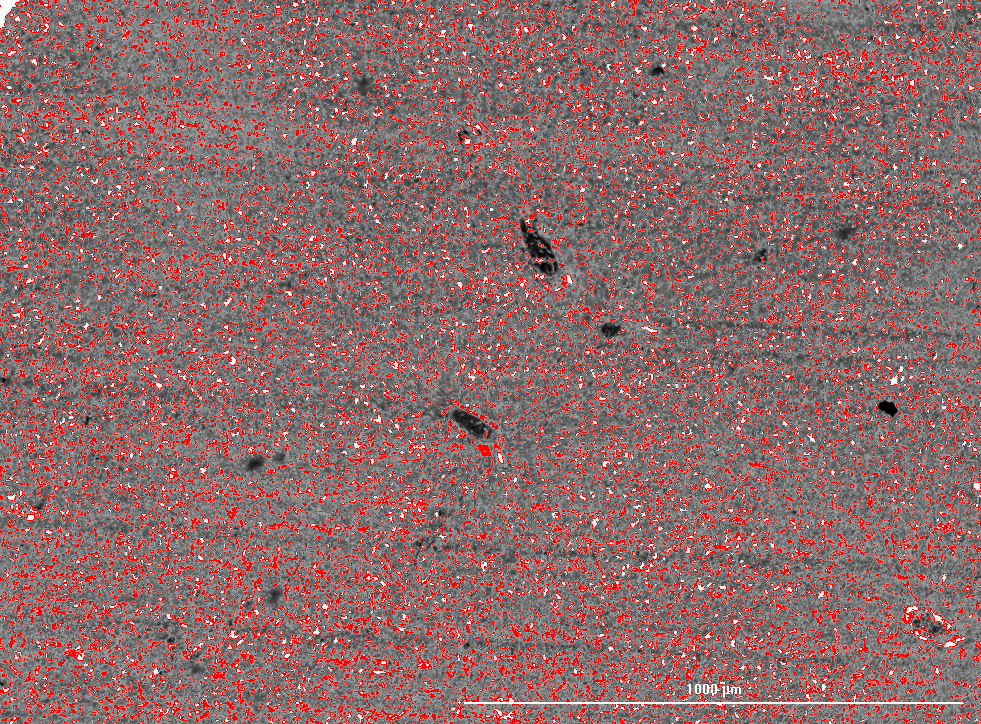

Supplement: S4 File — (ZIP) [file pone.0306020.s004.zip › fk506_2_bluesaturation.png]

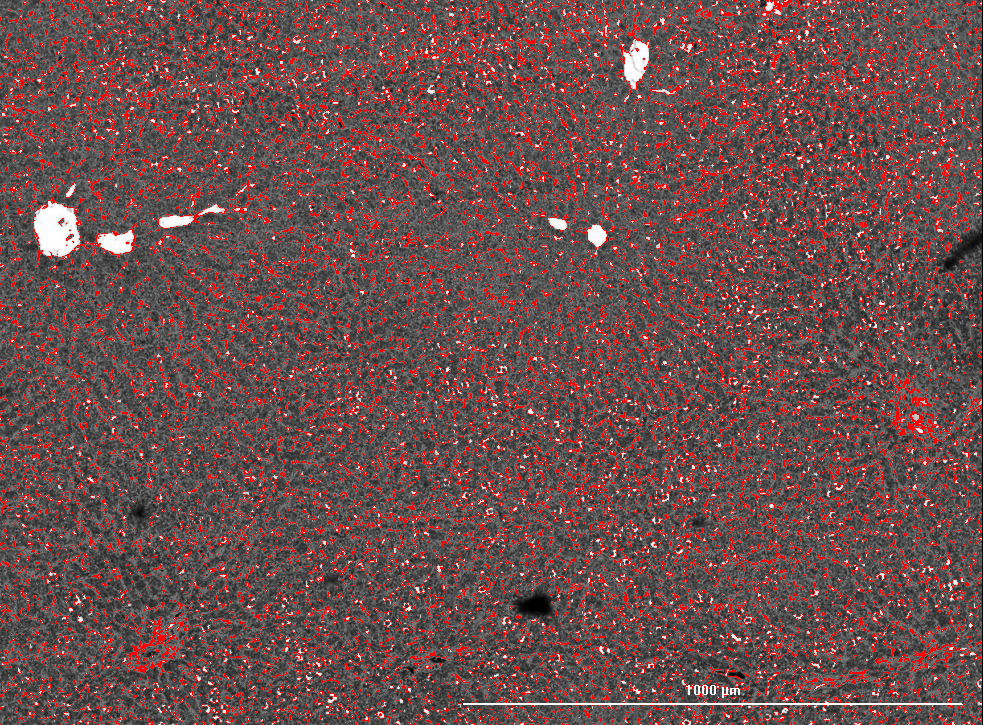

Supplement: S4 File — (ZIP) [file pone.0306020.s004.zip › fk506_3_bluesaturation.png]

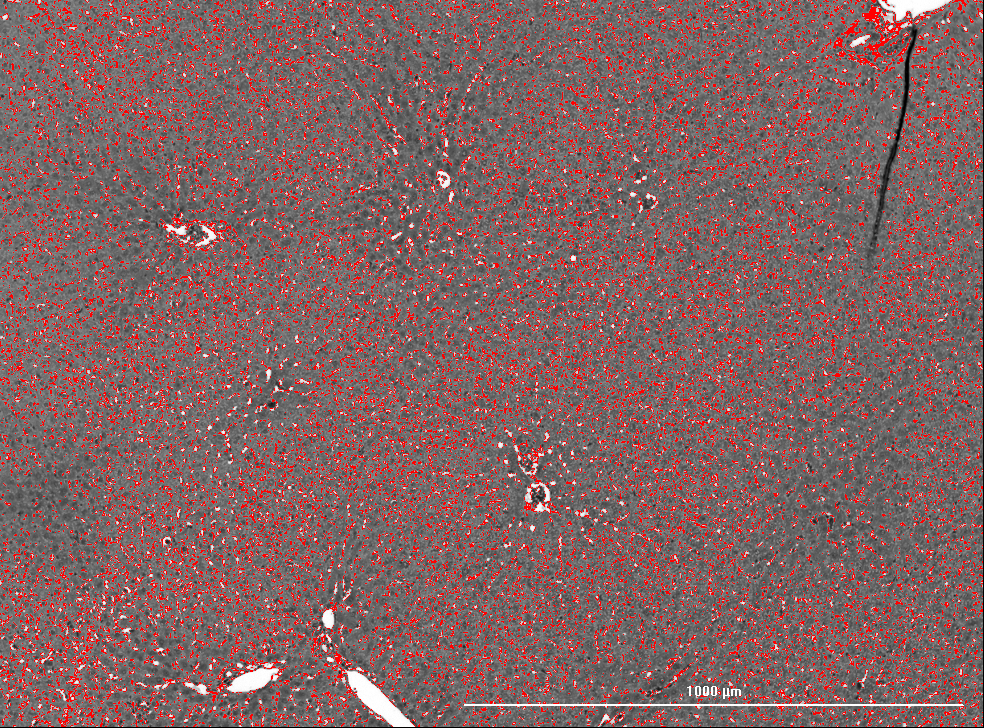

Supplement: S4 File — (ZIP) [file pone.0306020.s004.zip › fk506_4_bluesaturation.png]

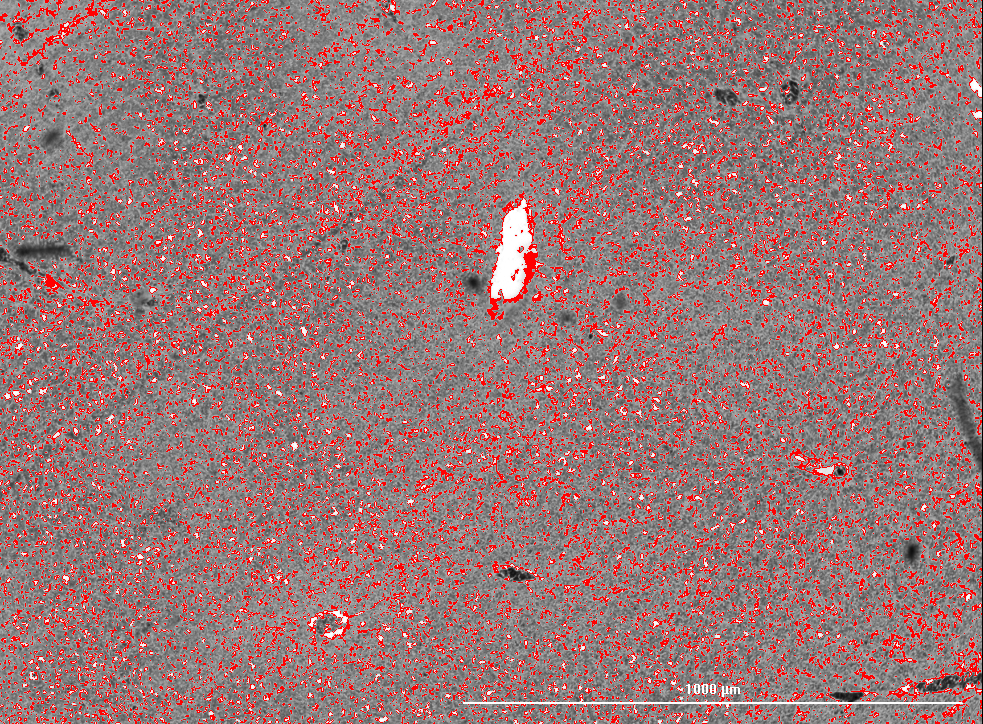

Supplement: S4 File — (ZIP) [file pone.0306020.s004.zip › fk506_5_bluesaturation.png]

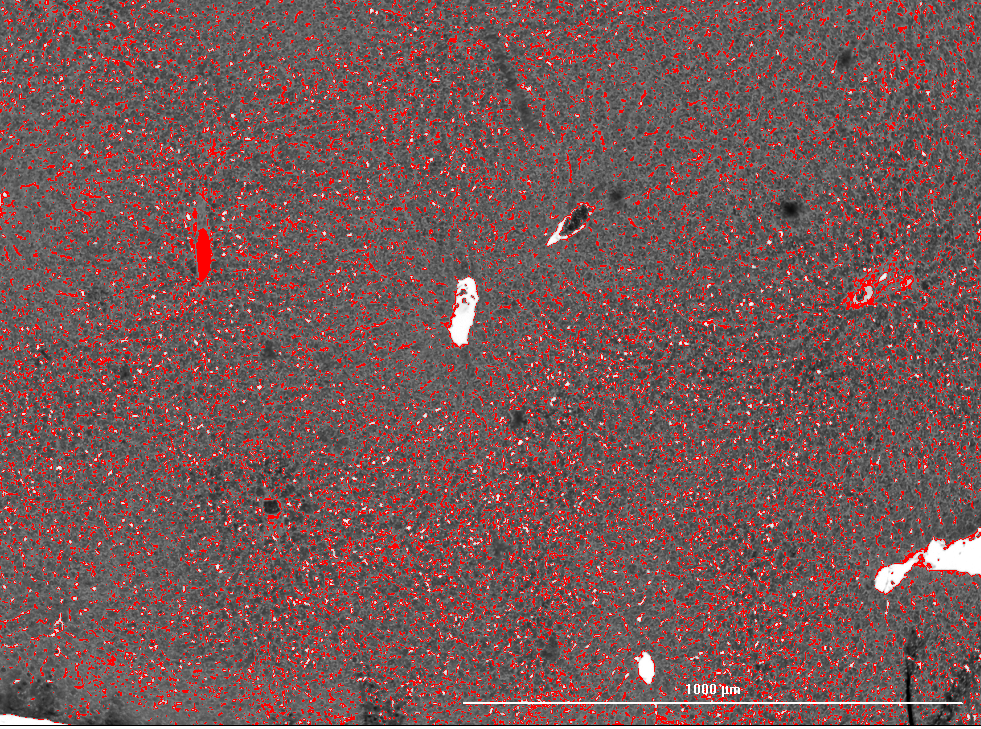

Supplement: S4 File — (ZIP) [file pone.0306020.s004.zip › fk506_6_bluesaturation.png]

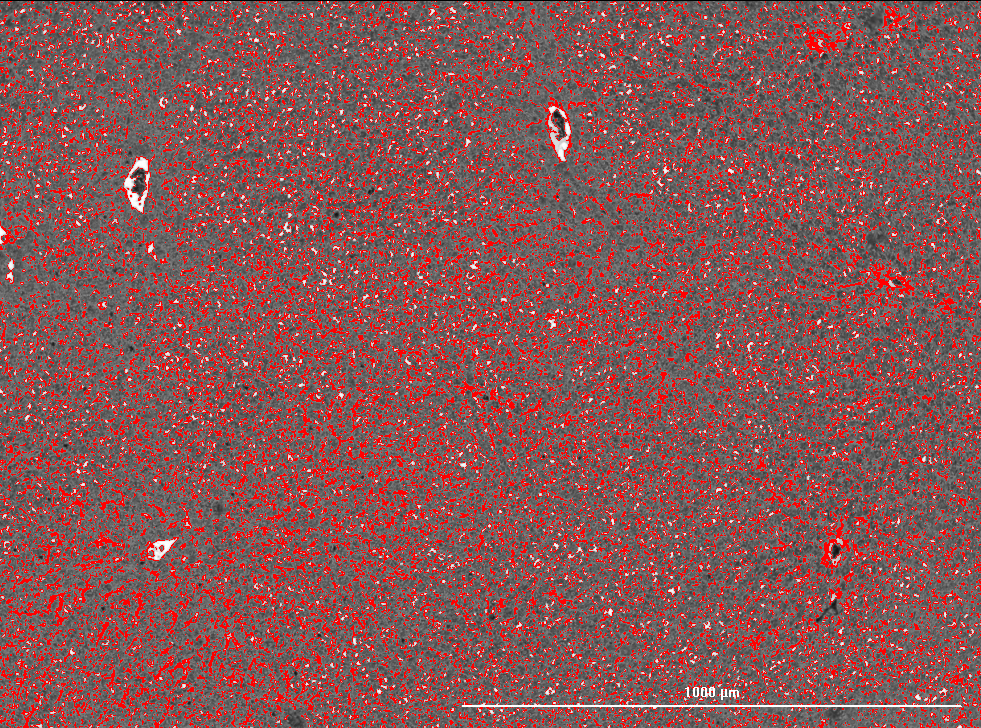

Supplement: S4 File — (ZIP) [file pone.0306020.s004.zip › fk506_7_bluesaturation.png]

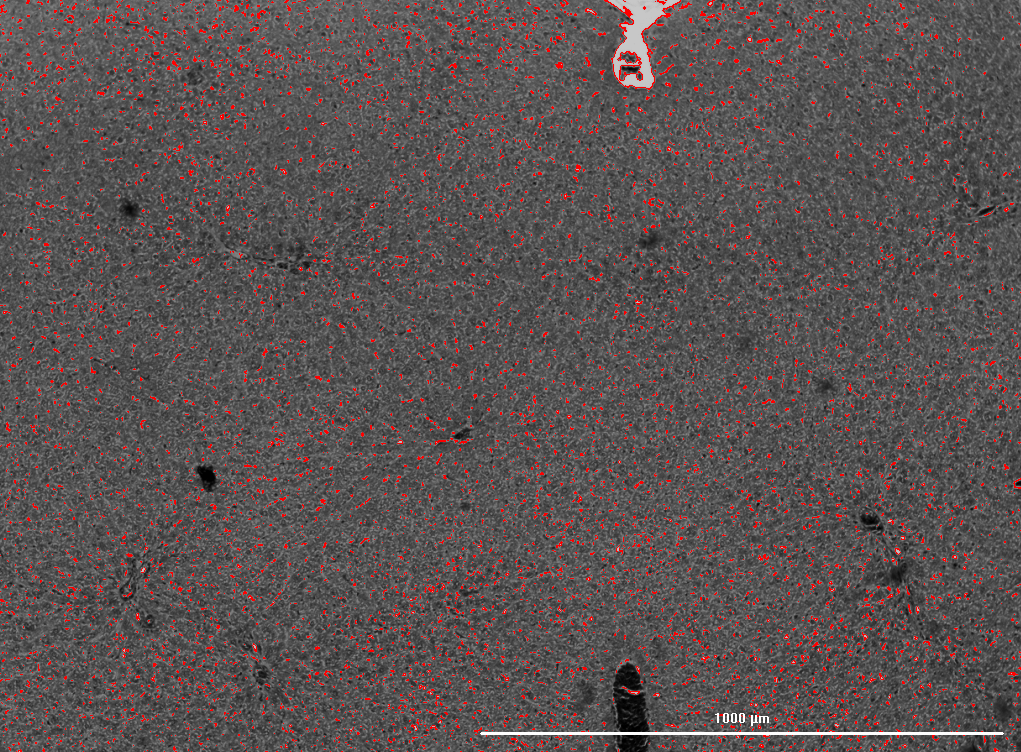

Supplement: S4 File — (ZIP) [file pone.0306020.s004.zip › fk506_8_bluesaturation.png]

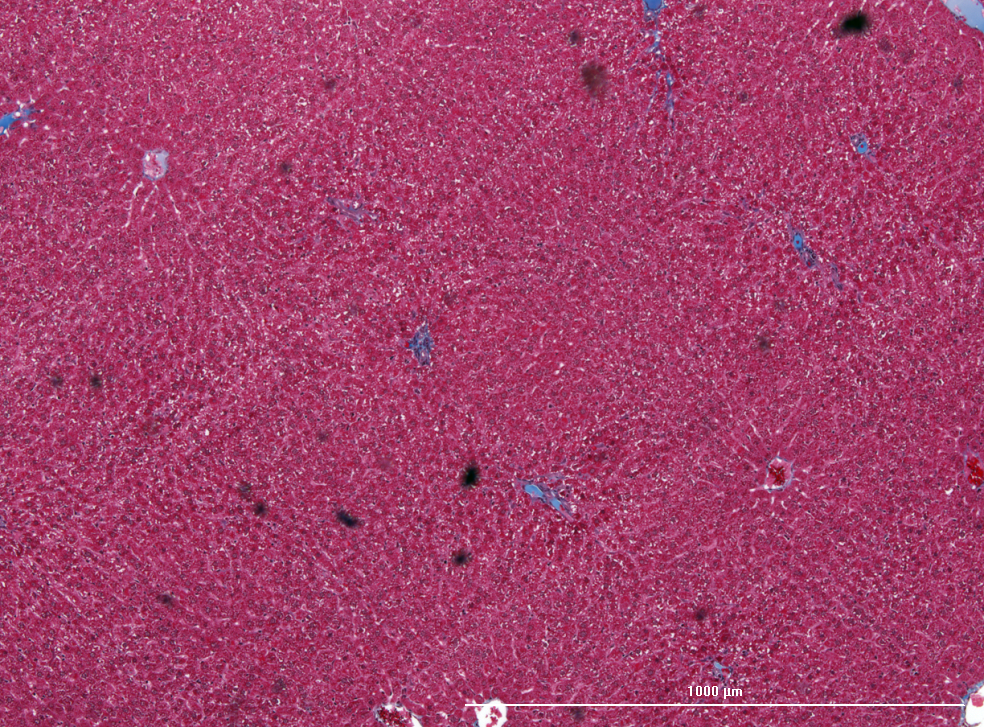

Supplement: S5 File — . (ZIP) [file pone.0306020.s005.zip › veh 1.jpg]

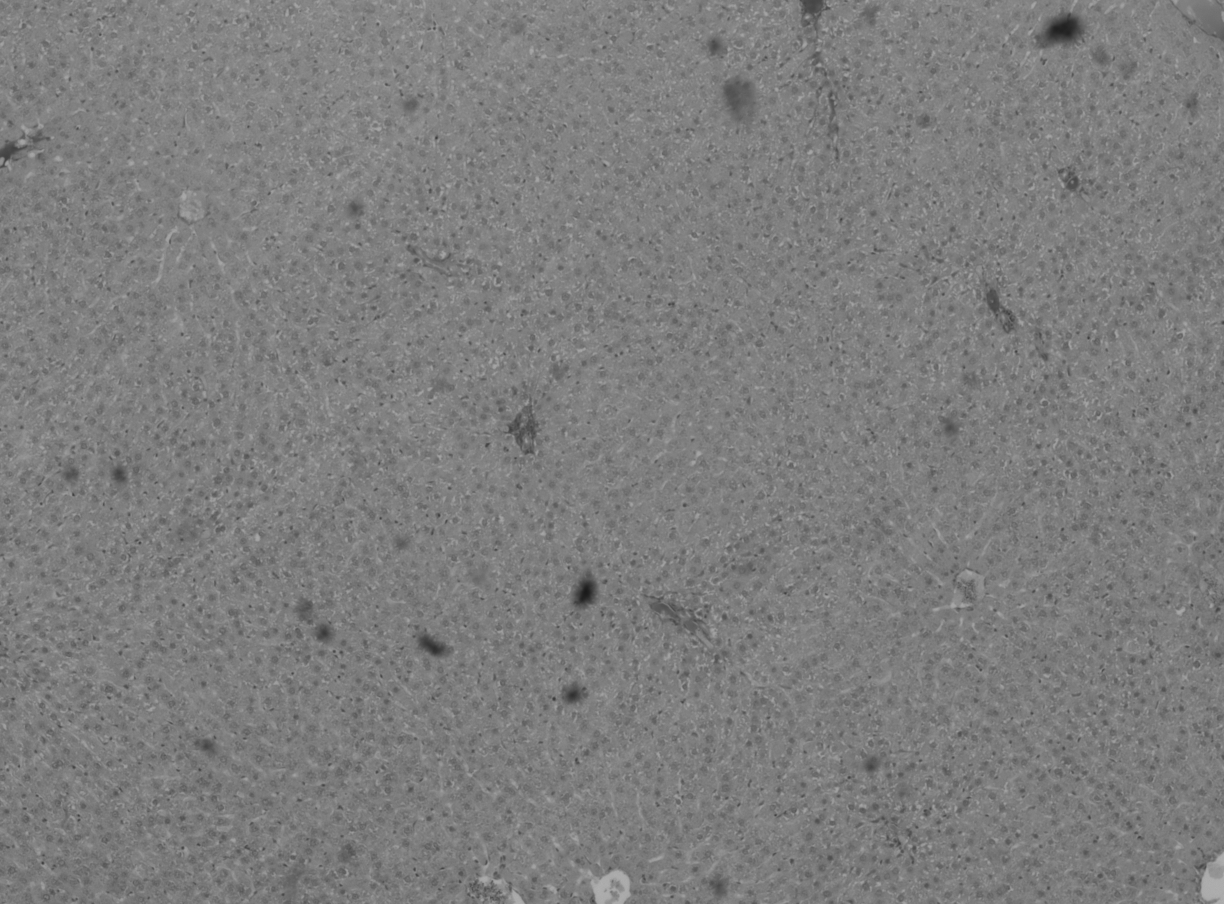

Supplement: S5 File — . (ZIP) [file pone.0306020.s005.zip › veh 1.tif]

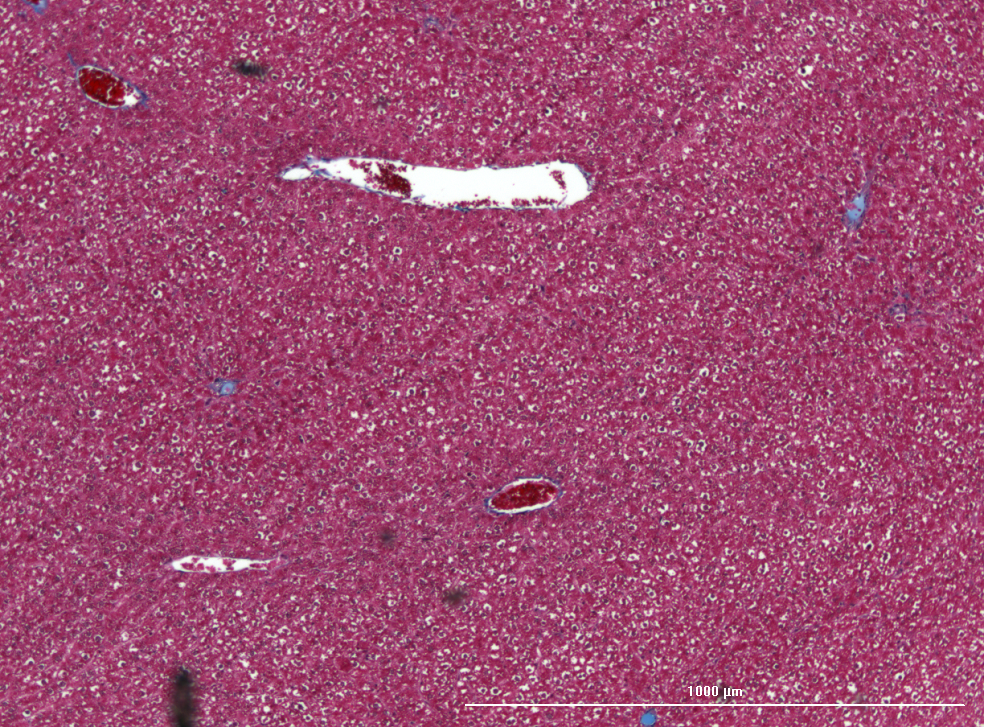

Supplement: S5 File — . (ZIP) [file pone.0306020.s005.zip › veh 2.jpg]

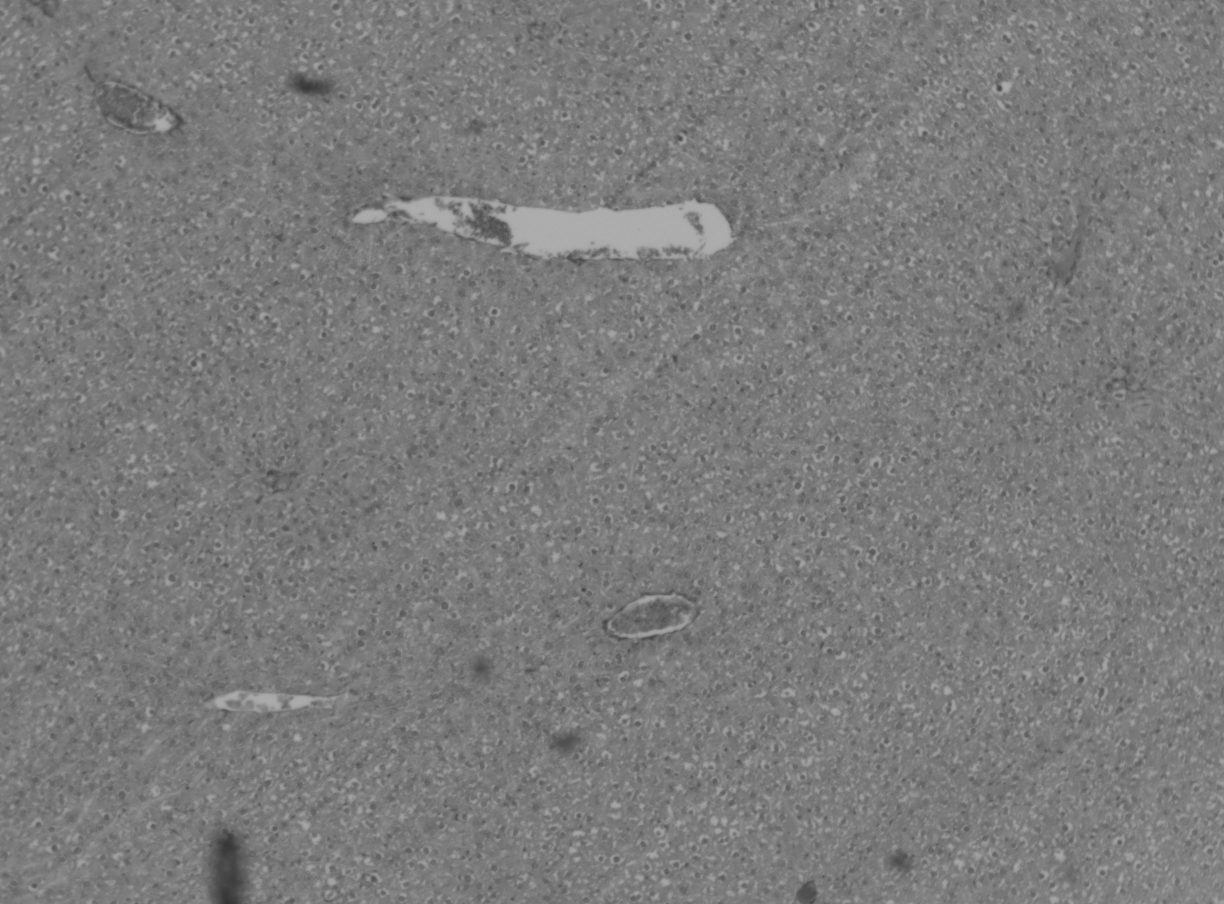

Supplement: S5 File — . (ZIP) [file pone.0306020.s005.zip › veh 2.tif]

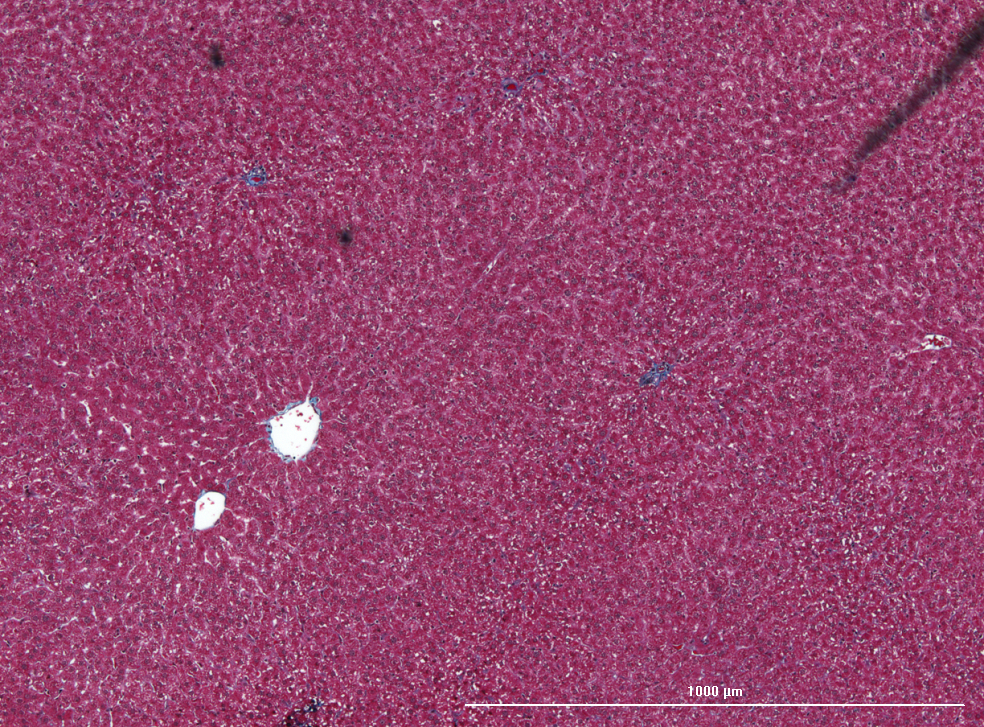

Supplement: S5 File — . (ZIP) [file pone.0306020.s005.zip › veh 3.jpg]

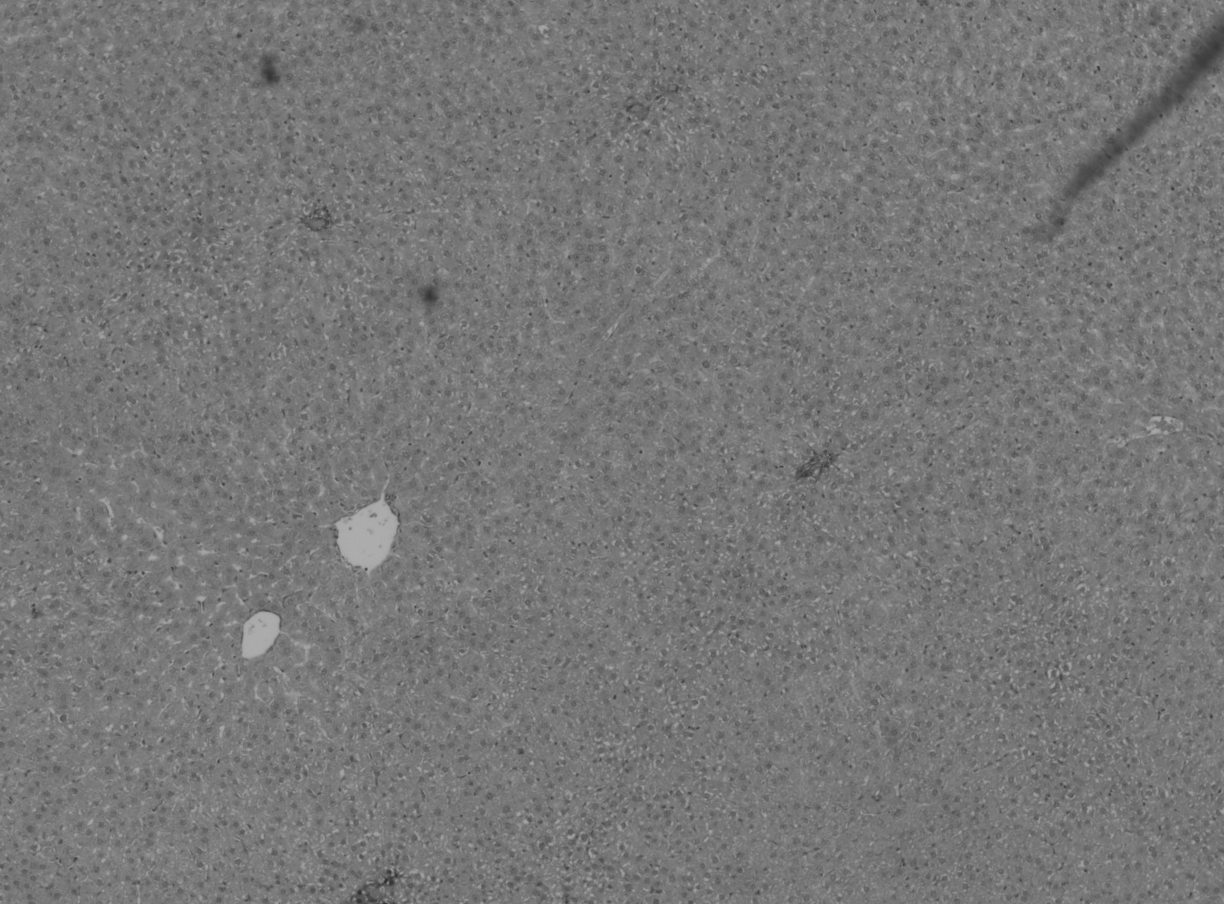

Supplement: S5 File — . (ZIP) [file pone.0306020.s005.zip › veh 3.tif]

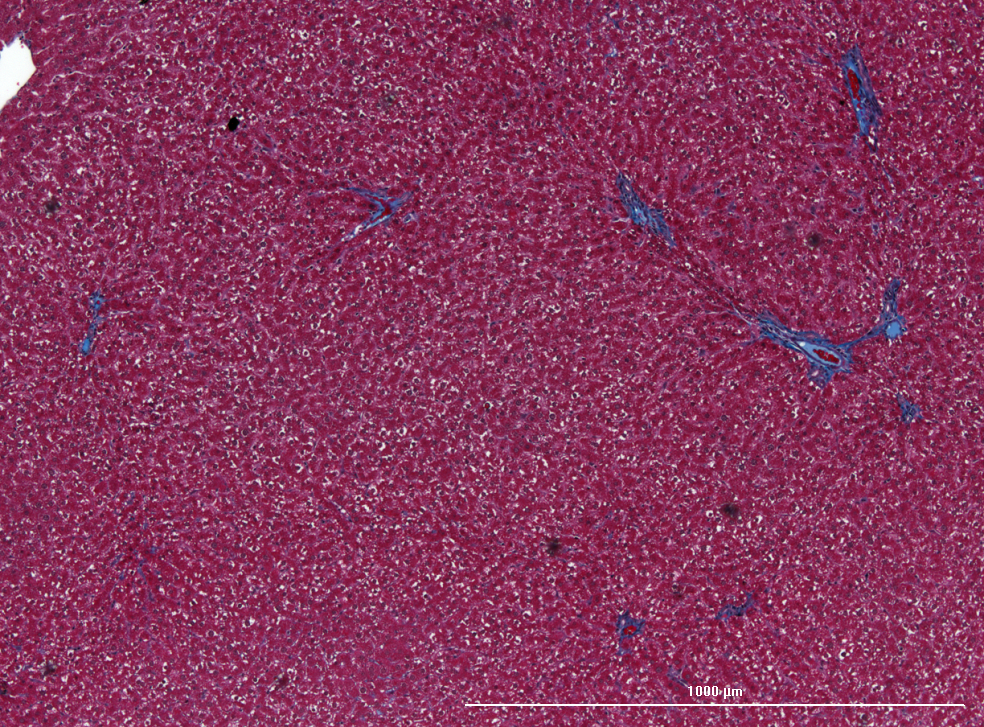

Supplement: S5 File — . (ZIP) [file pone.0306020.s005.zip › veh 4.jpg]

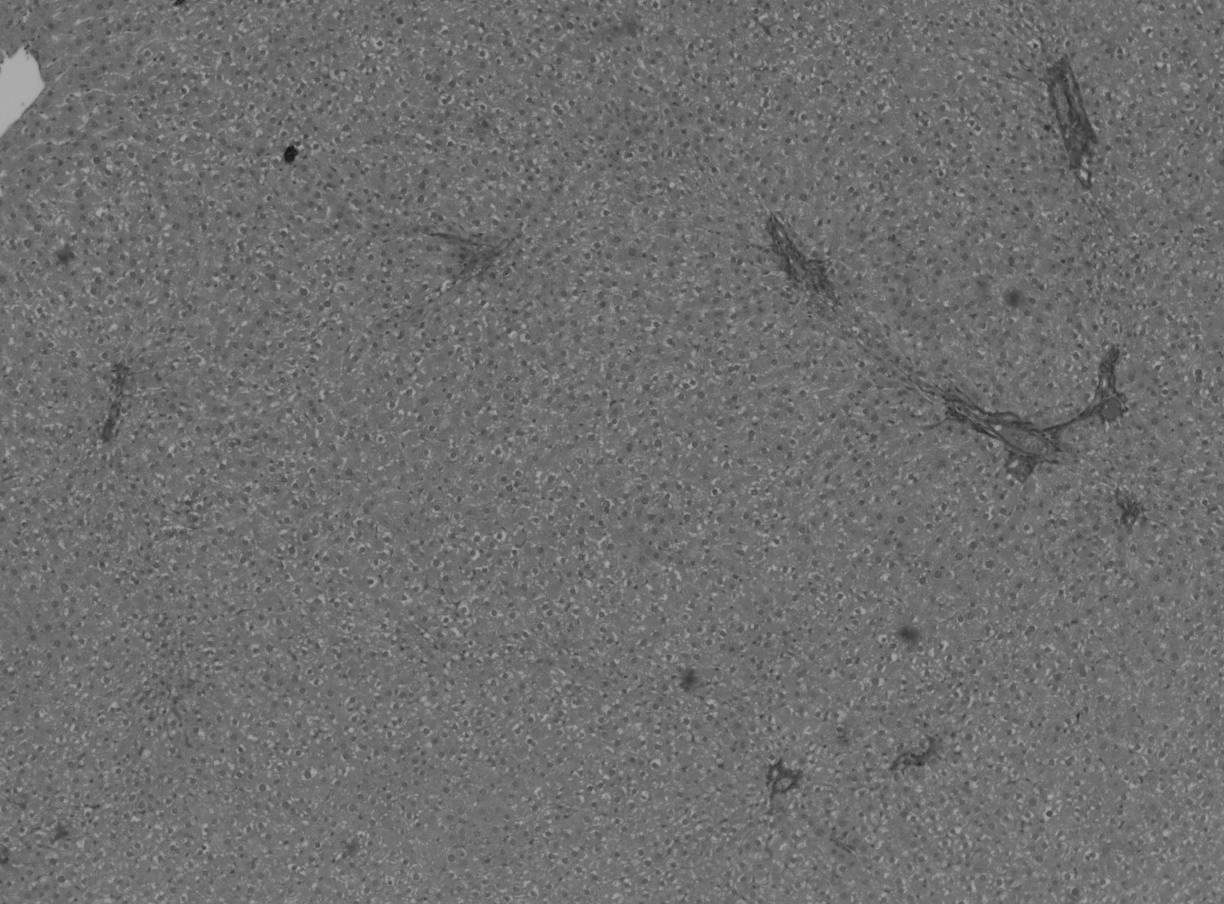

Supplement: S5 File — . (ZIP) [file pone.0306020.s005.zip › veh 4.tif]

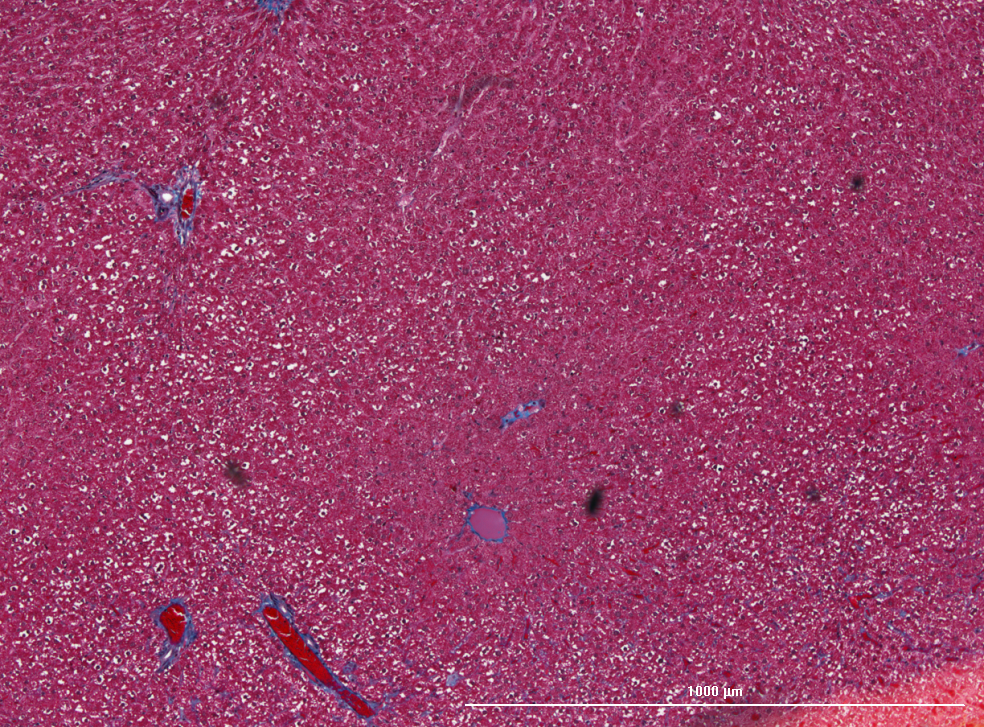

Supplement: S5 File — . (ZIP) [file pone.0306020.s005.zip › veh 5.jpg]

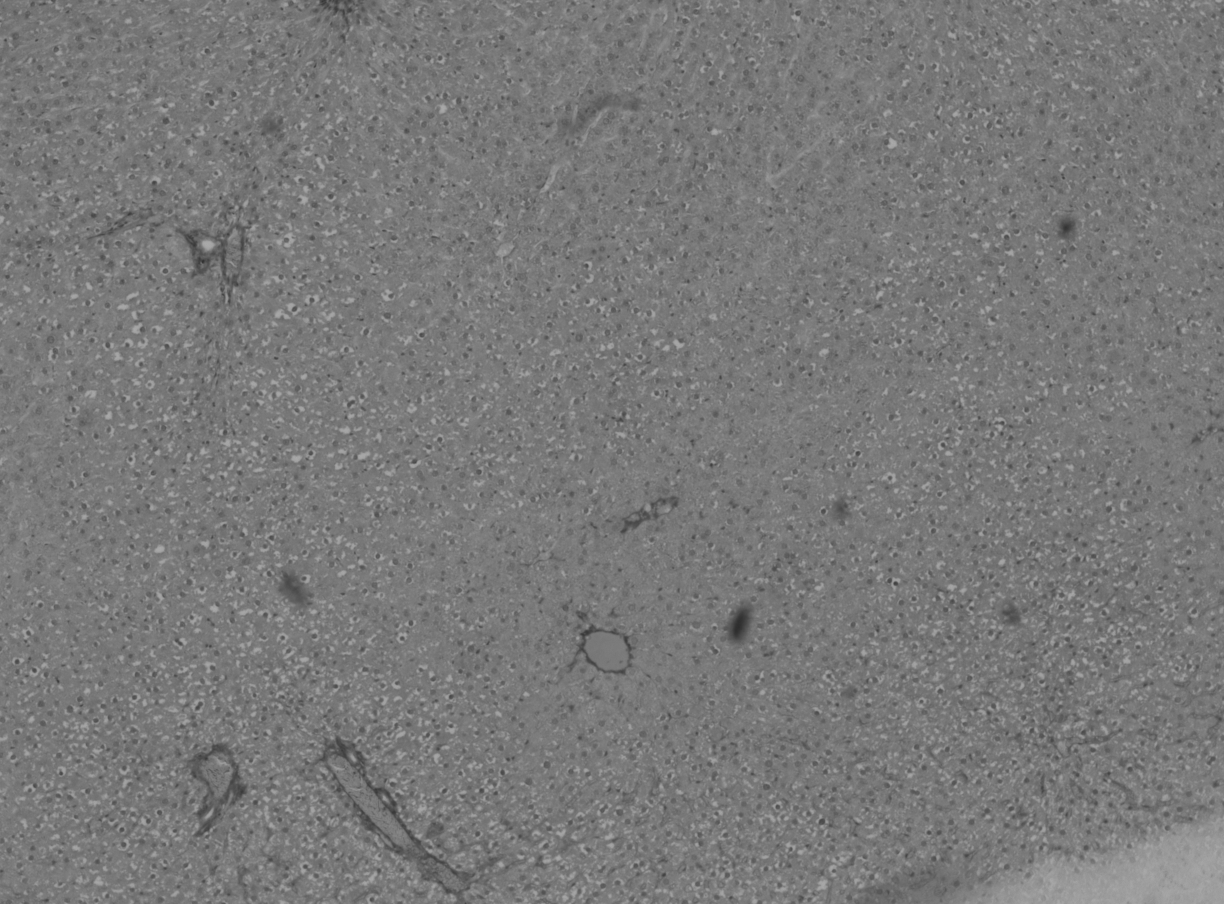

Supplement: S5 File — . (ZIP) [file pone.0306020.s005.zip › veh 5.tif]

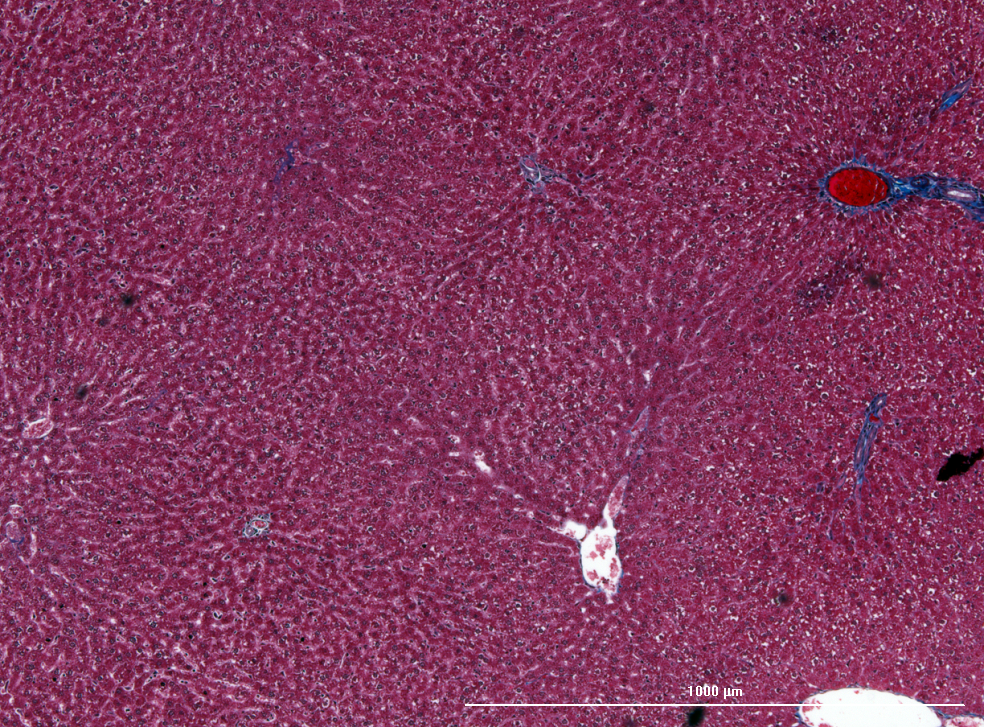

Supplement: S5 File — . (ZIP) [file pone.0306020.s005.zip › veh 6.jpg]

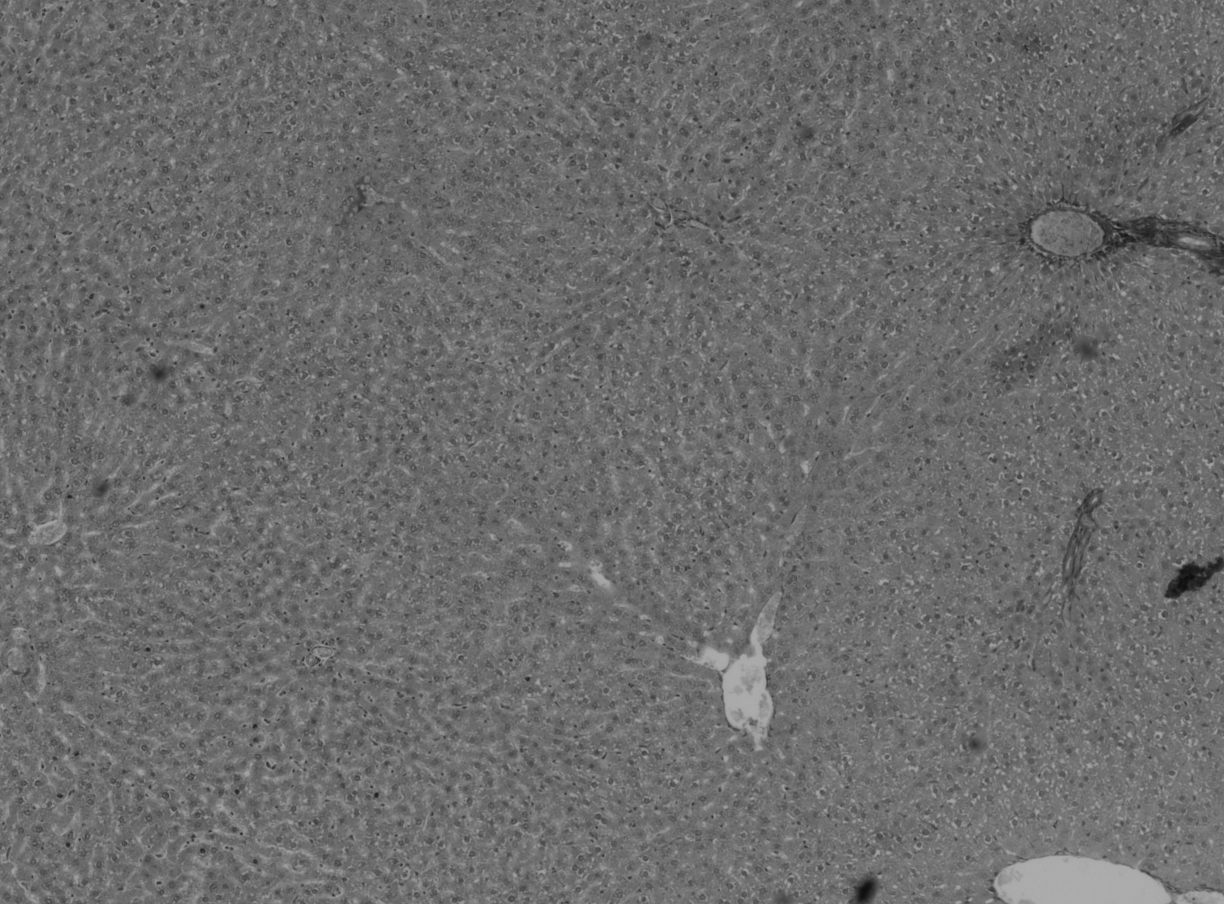

Supplement: S5 File — . (ZIP) [file pone.0306020.s005.zip › veh 6.tif]

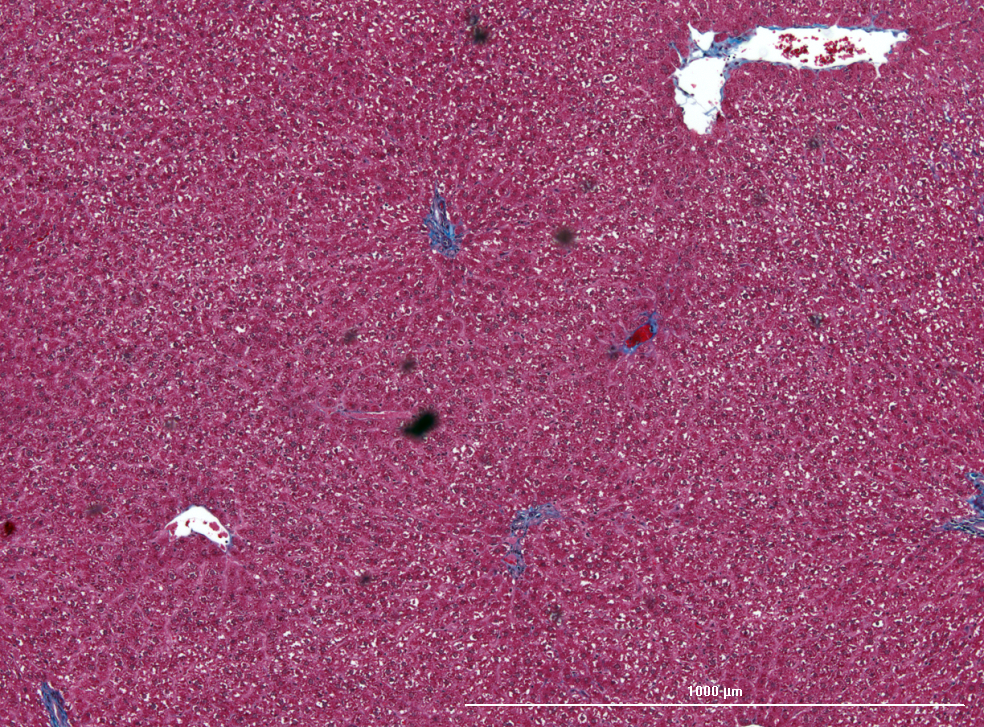

Supplement: S5 File — . (ZIP) [file pone.0306020.s005.zip › veh 7.jpg]

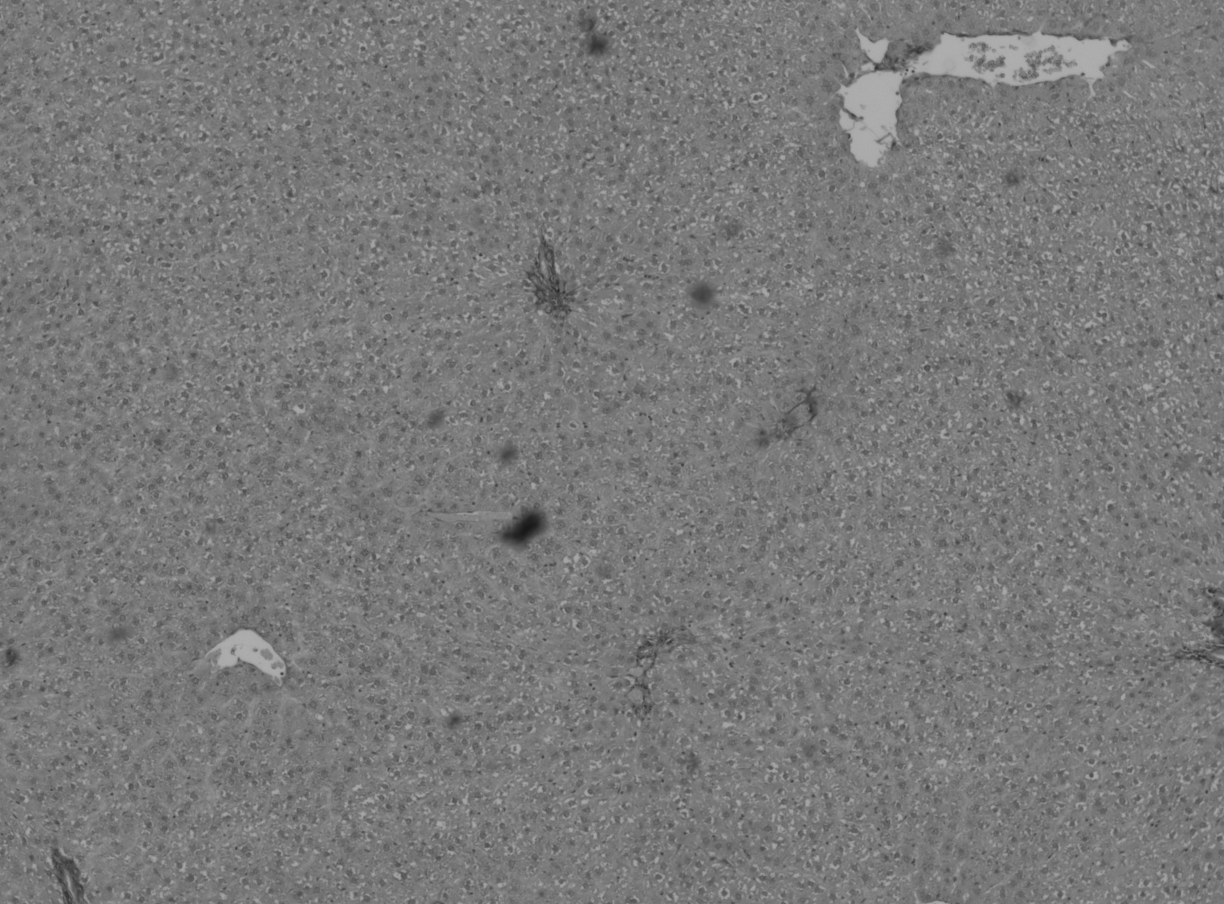

Supplement: S5 File — . (ZIP) [file pone.0306020.s005.zip › veh 7.tif]

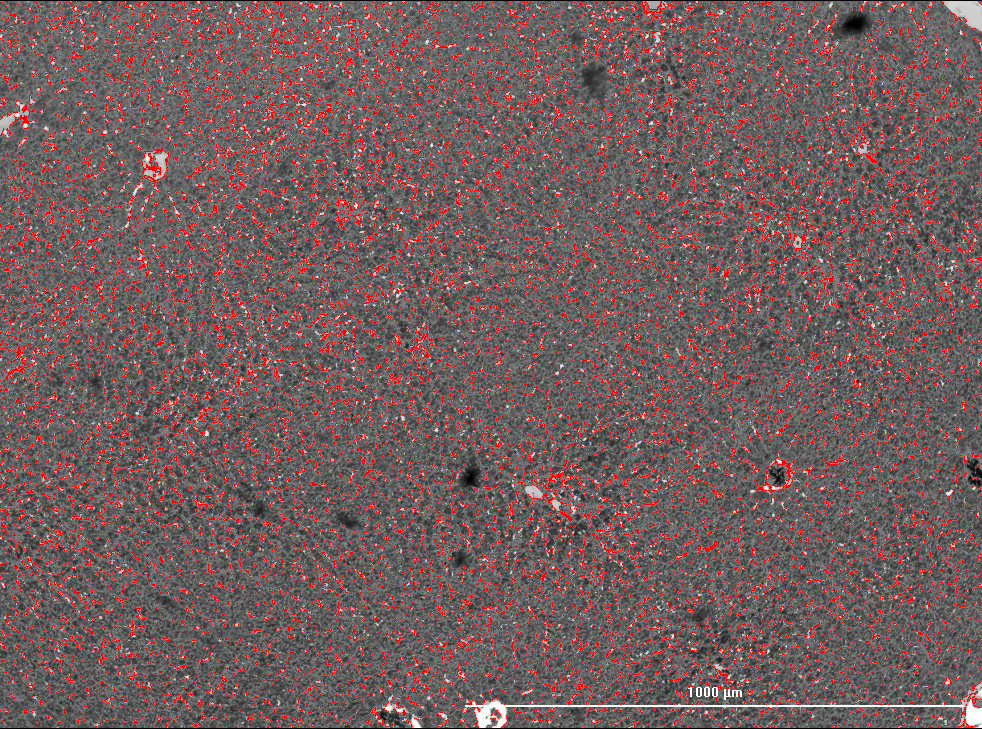

Supplement: S5 File — . (ZIP) [file pone.0306020.s005.zip › veh1_bluesaturation.png]

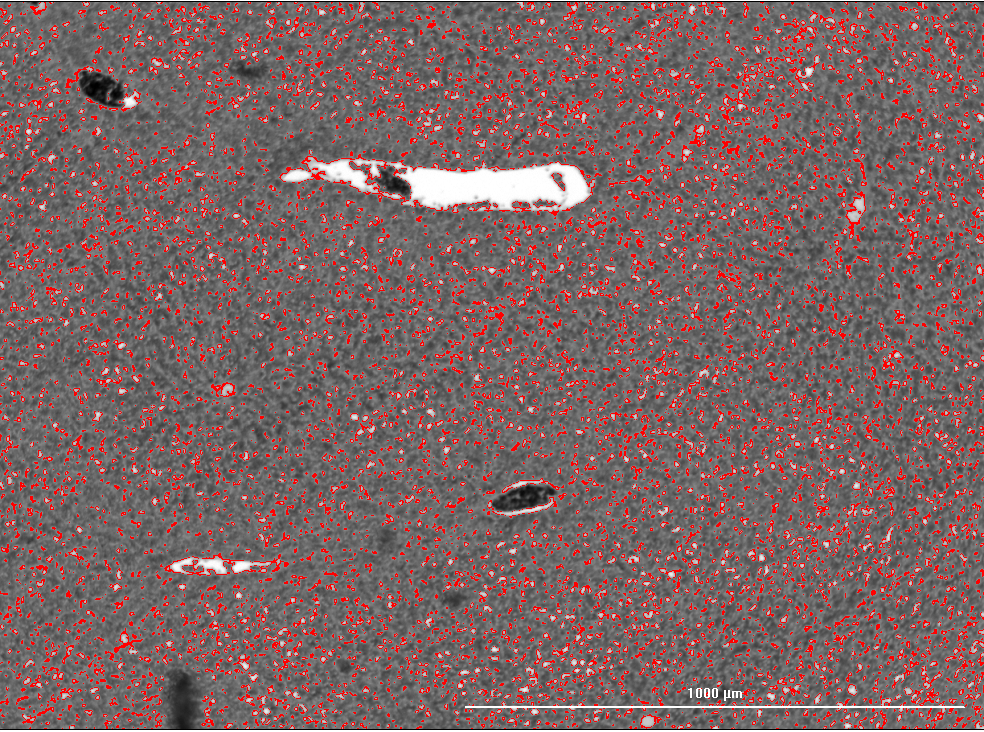

Supplement: S5 File — . (ZIP) [file pone.0306020.s005.zip › veh2_bluesaturation.png]

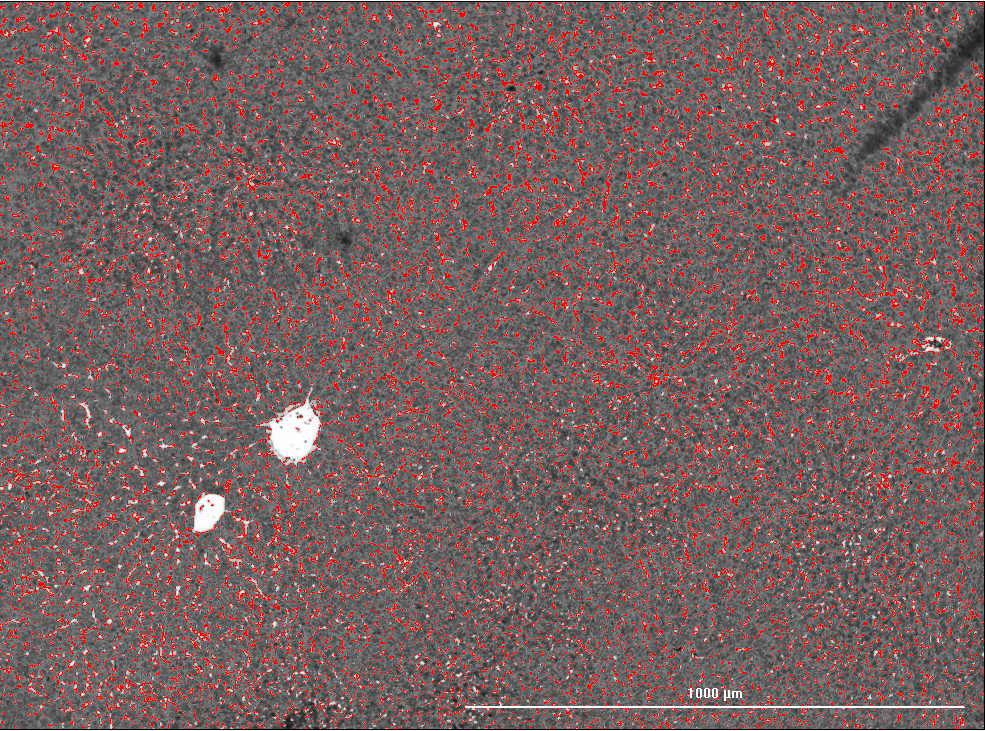

Supplement: S5 File — . (ZIP) [file pone.0306020.s005.zip › veh3_bluesaturation.png]

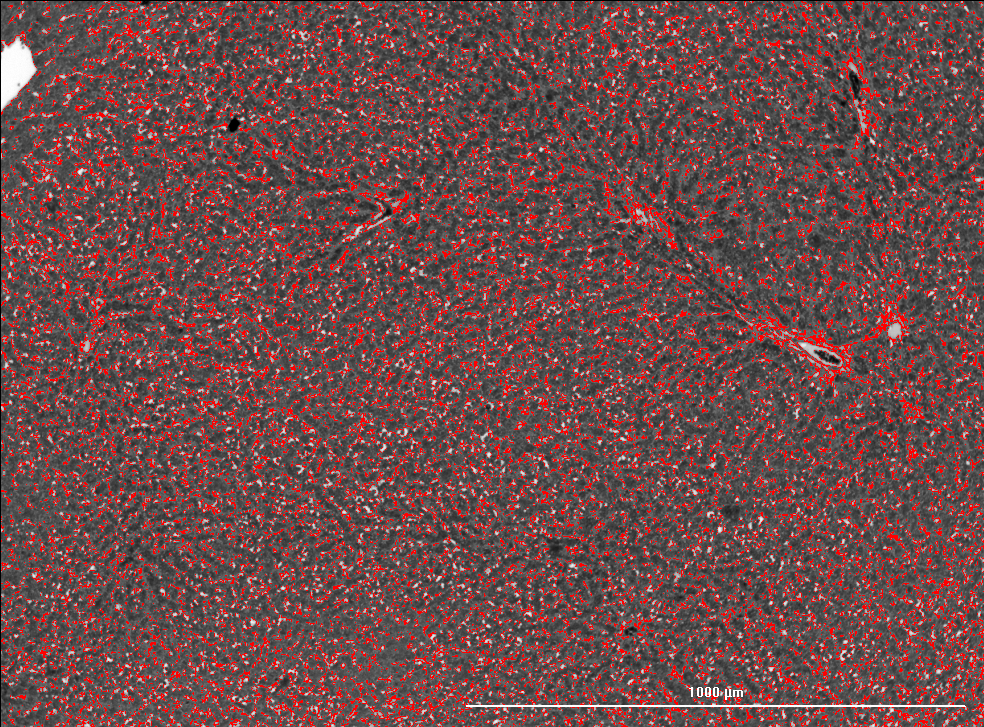

Supplement: S5 File — . (ZIP) [file pone.0306020.s005.zip › veh4_bluesaturation.png]

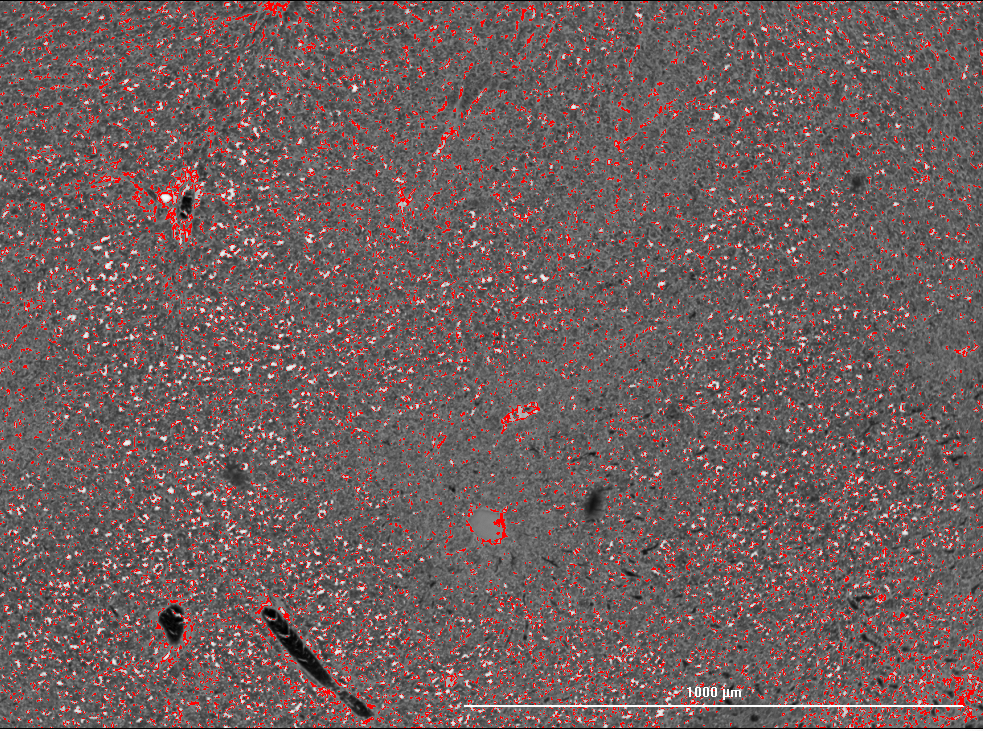

Supplement: S5 File — . (ZIP) [file pone.0306020.s005.zip › veh5_bluesaturation.png]

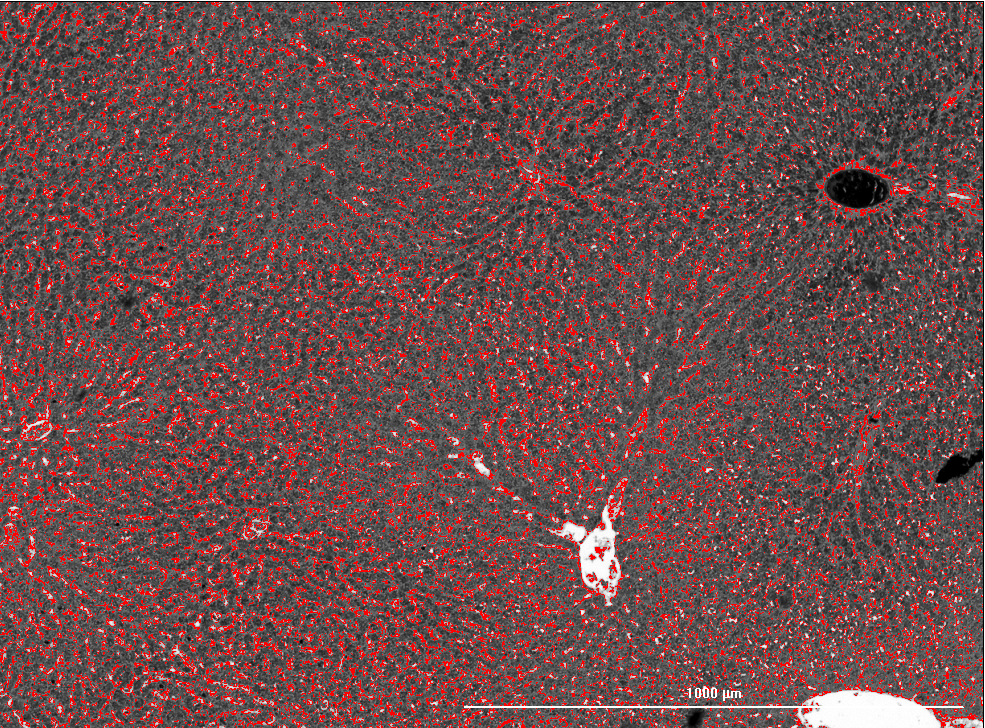

Supplement: S5 File — . (ZIP) [file pone.0306020.s005.zip › veh6_bluesaturation.png]

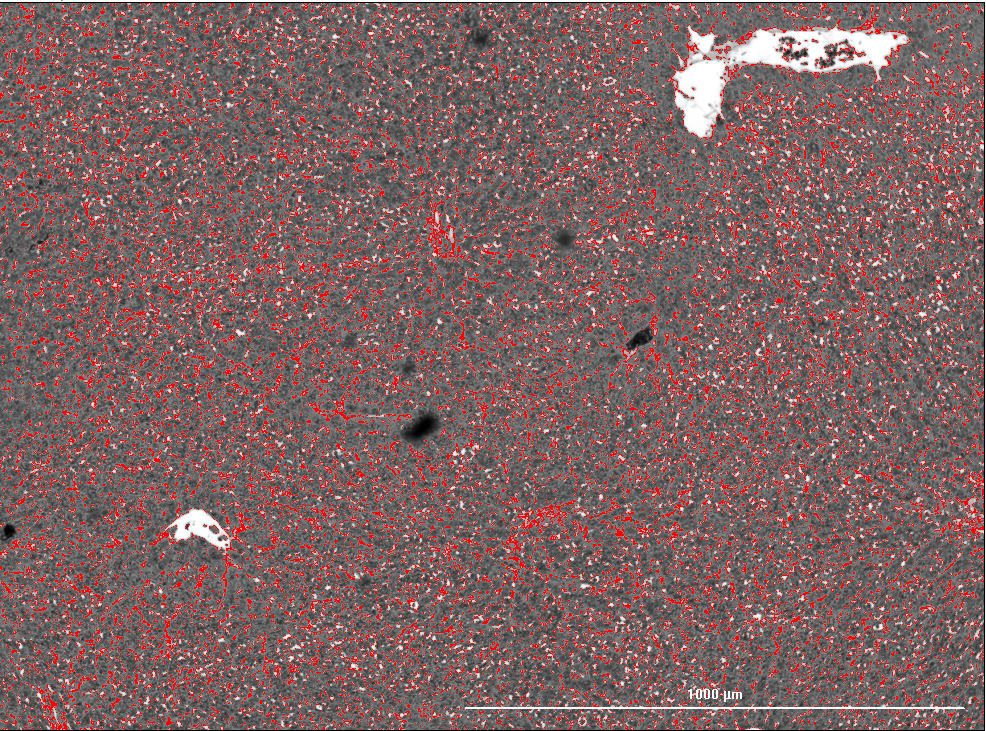

Supplement: S5 File — . (ZIP) [file pone.0306020.s005.zip › veh7_bluesaturation.png]
