# Supplementary material for: Correction: Tacrolimus (FK506) Prevents Early Stages of Ethanol Induced Hepatic Fibrosis by Targeting LARP6 Dependent Mechanism of Collagen Synthesis
Source: PLoS One. 2024 Jun 20;19(6):e0306020. doi: 10.1371/journal.pone.0306020 (PMC11189226; doi:10.1371/journal.pone.0306020)
Supplement: S5 File — . (ZIP) [file pone.0306020.s005.zip › method.docx]

I have added following files:

1. newly taken images of histology slides as tif and png images.

2. new fig. 3c which contains the plot and calculations based on new images

3. ImageJ excel file which contains raw data

4. bluesaturation.png images (for each image we have generate a blue saturation representation (in red))

Description of the Image J process:

1. I had to convert all TIF into JPG

2. Open single image in ImageJ then go to ImageColorSplit Channels (this will allow you to split the color channels)

3. Remove green and red color splits

4. In ImageJ select ImageAdjustThreshold (thresholds used are shown in the excel table as threshold1 and Threshold2)

5. In ImageJ select AnalysisMeasure

*note that some images had a lot of variation in total contrast so the thresholds had to be slightly adjusted to match the blue Hue.

For statistics I performed the same ordinary ANOVA with Turkey’s multiple comparisons test.
