# Supplementary material for: Correction: Tacrolimus (FK506) Prevents Early Stages of Ethanol Induced Hepatic Fibrosis by Targeting LARP6 Dependent Mechanism of Collagen Synthesis
Source: PLoS One. 2024 Jun 20;19(6):e0306020. doi: 10.1371/journal.pone.0306020 (PMC11189226; doi:10.1371/journal.pone.0306020)
Supplement: S5 File — . (ZIP) [file pone.0306020.s005.zip › new fig 3c.pptx]

## Slide 1
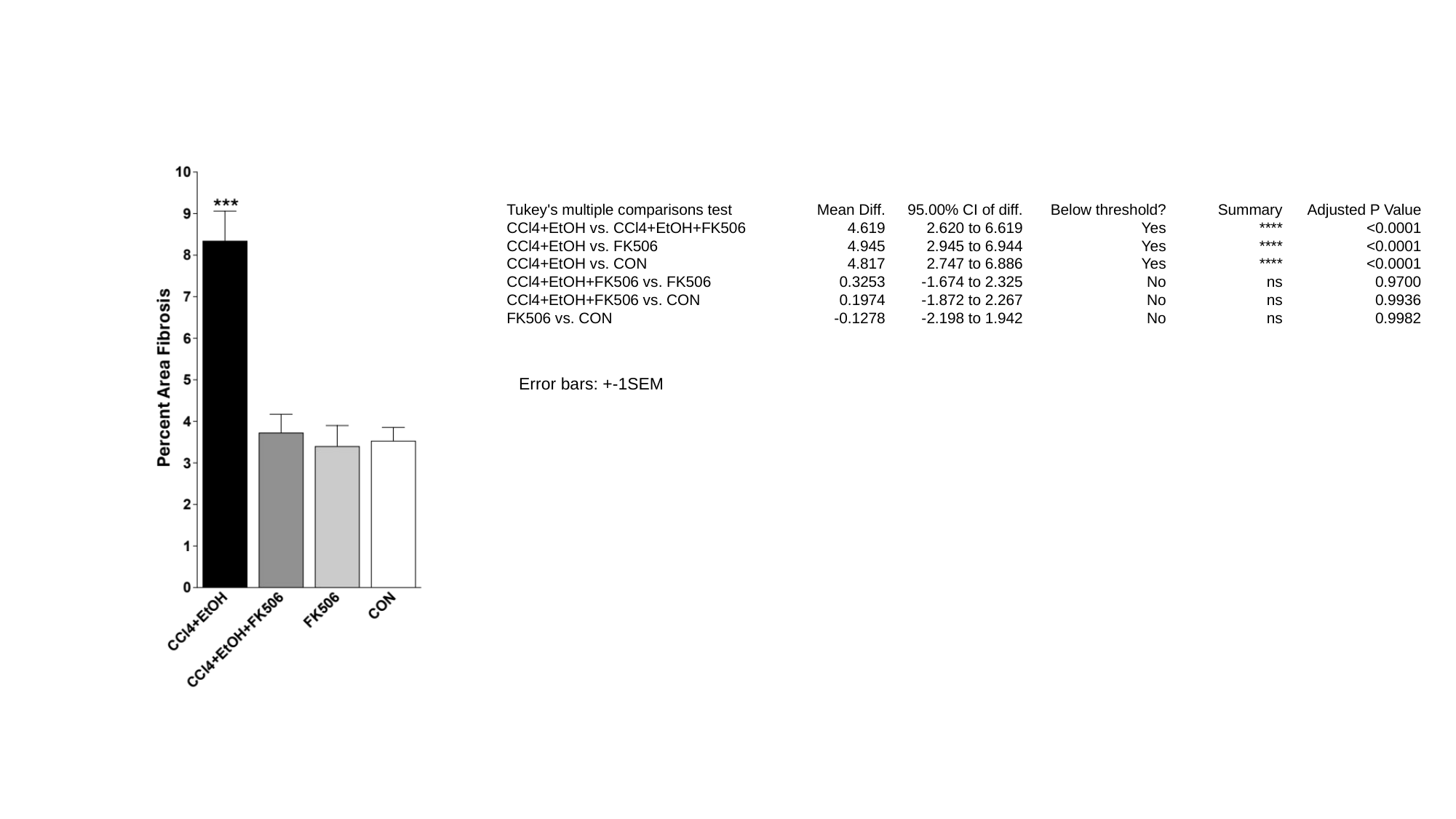

| Tukey's multiple comparisons test | Mean Diff. | 95.00% CI of diff. | Below threshold? | Summary | Adjusted P Value |
| --- | --- | --- | --- | --- | --- |
| CCl4+EtOH vs. CCl4+EtOH+FK506 | 4.619 | 2.620 to 6.619 | Yes | \*\*\*\* | <0.0001 |
| CCl4+EtOH vs. FK506 | 4.945 | 2.945 to 6.944 | Yes | \*\*\*\* | <0.0001 |
| CCl4+EtOH vs. CON | 4.817 | 2.747 to 6.886 | Yes | \*\*\*\* | <0.0001 |
| CCl4+EtOH+FK506 vs. FK506 | 0.3253 | -1.674 to 2.325 | No | ns | 0.9700 |
| CCl4+EtOH+FK506 vs. CON | 0.1974 | -1.872 to 2.267 | No | ns | 0.9936 |
| FK506 vs. CON | -0.1278 | -2.198 to 1.942 | No | ns | 0.9982 |
Error bars: +-1SEM
